# Supplementary material for: Suppressing quantum errors by scaling a surface code logical qubit
Source: Nature. 2023 Feb 22;614(7949):676–81. doi: 10.1038/s41586-022-05434-1 (PMC9946823; doi:10.1038/s41586-022-05434-1)
Supplement: Supplementary file 1 — This file contains Supplementary Sections 13–21, Figs. 1–34, a full list of members of Google Quantum AI and References. [file 41586_2022_5434_MOESM1_ESM.pdf]

nature portfolio

https://doi.org/10.1038/s41586-022-05434-1

Supplementary information

Suppressing quantum errors by scaling a surface code logical qubit

In the format provided by the authors and unedited

Supplemental materials for *Suppressing quantum errors by scaling a surface code logical qubit*

Google Quantum AI
(Dated: October 7, 2022)

CONTENTS

I. Experimental details 1
- A. Idling errors 1
- B. Optimizing gate parameters 2
- C. Single qubit gates 2
- D. Two qubit gates 2
- E. Measurement and reset 4
- F. Microwave crosstalk induced leakage mitigation 4
- G. Surface code experimental details 4
  - 1. Surface code circuits 4
  - 2. Choice of distance-3 grids 6
  - 3. Order of experiments 6
  - 4. Initial states 7
- H. Repetition code experiment 7

II. Decoding 8
- A. Setting the prior distribution 8
- B. Correlated minimum-weight perfect matching 11
- C. Belief-matching 11
- D. Tensor network decoding 12

III. Fitting error versus cycles to extract logical error per cycle 13

IV. Simulation of logical memory experiment 13
- A. Noise models 15
  - 1. Decay, dephasing, and leakage heating 15
  - 2. Readout and reset error 15
  - 3. Leakage 16
  - 4. Crosstalk 16
  - 5. Depolarizing errors 17
- B. Simulator details 17
  - 1. kraus_sim - noisy circuit compiler and quantum simulator 17
  - 2. Pauli+ simulator implementation 17
  - 3. Generalized twirling approximation 20
- C. Comparison of Pauli+ and quantum simulations 21

V. Sensitivity of logical error per cycle to physical errors 22
- A. Experimental operation point 23
- B. Logical error per cycle sensitivities for distance-3/5 surface codes 24
- C. Logical error per cycle sensitivity vs $1/\Lambda_{3/5}$ sensitivity 24

VI. Diagnostics using p_{ij} of surface codes 24
- A. Experiment *vs.* Pauli+ p_{ij} correlation matrices 26
- B. Probabilities in p_{ij} of main error edges in the surface code 26
  - 1. Timelike edges 27
  - 2. Spacelike edges 27
  - 3. Spacetimelike edges 28
- C. Probability using generalized p_{ij} of clusters of detection events 30

VII. Surface code $1/\Lambda_{3/5}$ error budget 31

VIII. Overview of error correction experiments 32

IX. Additional plots of surface code data 32

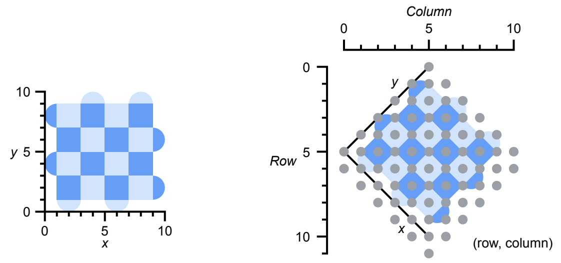

FIG. S1. **Qubit coordinates.** Left: Distance-5 surface code as presented in the main text, with an (x, y) coordinate system indicated. Right: Rotated coordinate system used for heatmap plots in the supplement, using (row, column).

I. EXPERIMENTAL DETAILS

A. Idling errors

A significant contribution to the logical error budget is data qubit decoherence during the readout and reset of the measure qubits. The primary decoherence mechanism is dephasing induced by low frequency flux noise. We mitigate dephasing through dynamical decoupling with XY-4 phase cycling [61], which compensates first-order pulse errors and protects arbitrary quantum states.

The specific dynamical decoupling sequence is chosen on a per-qubit basis in order to tailor the sequence filter function to each qubit's unique noise environment. In particular, we allow the number of pulses, and hence the inter-pulse spacing, to vary for each qubit addressed.

B. Optimizing gate parameters

Our quantum processor employs frequency-tunable qubits on a two-dimensional lattice with coupler-mediated nearest-neighbor coupling and dispersive readout. Quantum logic is implemented via single-qubit (SQ), two-qubit (CZ), and readout (RO) gates. SQ gates are implemented via resonant microwave pulses at each qubit's respective $f_{0\to1}$ idle frequency. CZ gates are implemented by frequency tuning qubit pairs into $f_{0\to1}/f_{1\to2}$ resonance at respective *interaction* frequencies and actuating respective couplers. RO gates are implemented by sweeping qubits' $f_{0\to1}$ frequencies to respective *readout* frequencies and measuring them via variable-amplitude and -length readout pulses. Most error mechanisms depend strongly on gate frequencies and readout-pulse parameters, which we refer to collectively as *gate parameters*. Optimizing gate parameters is a critical error mitigation strategy necessary for state-of-the-art surface-code performance [31, 44].

To optimize gate parameters, we developed a surface-code objective through benchmarking and machine learning. It includes error contributions from relaxation, dephasing, crosstalk, and pulse distortion, along with various heuristics. It embeds the surface code circuit and it's mapping onto our processor. Furthermore, it is trained on parallel cross-entropy benchmarks with CZ gates operating according to the surface code circuit. To offer a sense of scale, the distance-5 objective incorporates $O(10^4)$ error terms and is defined over 49 idle, 49 readout, and 80 interaction frequencies, and 49 readout pulse amplitudes and 49 lengths. It is noisy, non-convex, and all parameters are explicitly or implicitly intertwined due to engineered interactions and/or crosstalk. Furthermore, since each parameter is constrained to $\sim 10^2$ values by the control electronics, processor circuit, and gate parameters, the search-space is $\sim 10^{552}$. This space is intractable to search exhaustively and traditional global optimizers do not perform well on the objective. Therefore, we invented the Snake optimizer to address it [62].

The Snake leverages concepts in graph optimization and dynamic programming to split complex high-dimensional optimization problems into simpler lower-dimensional subproblems. One key hyperparameter of the Snake is the subproblem dimension, which enables us to trade optimization complexity for accessible solutions. We operate the Snake in an intermediate dimensional regime that benefits from the speed of local search and non-locality of global search. This strategy outperforms local and global optimization in convergence rate and error on the distance-5 objective. Furthermore, we believe it will scale towards fault-tolerant processors.

To illustrate how the Snake trades between error mechanisms, we plot optimized idle and interaction frequencies as error mechanisms are progressively enabled in Fig. S2. Readout parameters experience similar tradeoffs but are not shown. After optimization, some gates unexpectedly experience uncharacteristically large error rates. These events happen randomly and are often due to spurious resonances, for example due to two-level-system defects, moving into the path of SQ and/or CZ gates [63]. Reoptimizing all gates after such failures is not scalable due to several considerations, including calibration runtime. Instead, we use the Snake to locally reoptimize failing gates and stitch solutions. To improve the quality and longevity of our solutions, we employ higher dimensional optimization and embed historical data into our objective.

C. Single qubit gates

As discussed in the previous section, single qubit gates are implemented through a combination of microwave XY rotations and virtual Z rotations. All microwave pulses are 25 ns in length, and utilize DRAG pulse shaping to mitigate leakage from off-resonant driving of the $|1\rangle \leftrightarrow |2\rangle$ transition, as detailed in [44]. We assess our single qubit gate performance through randomized benchmarking, acting on all qubits simultaneously. A summary of our single qubit device parameters, including operating frequencies, coherence times, and gate errors can be found in Fig. S3. We note all coherence times are measured at the qubit idle frequencies. Furthermore, the values of T_1 and T_2^{CPMG} fluctuate in time, resulting in cases where T_2^{CPMG} appears to exceed the known $2T_1$ limit. We also benchmark leakage out of the computational subspace, and through a combination of DRAG and microwave crosstalk compensation results, we measure a mean leakage rate of 5×10^{-5} per gate.

D. Two qubit gates

The two qubit gate employed in this work is the controlled-Z (CZ) gate. CZ gates in the Sycamore architecture are implemented by bringing the states $|11\rangle$ and $|02\rangle$ on resonance, then turning on the coupling such that the joint state swaps to $|02\rangle$ then comes back, accumulating a π phase shift in the process [16]. The gate takes 34 ns to complete. We assess our CZ gate performance by performing cross-entropy benchmarking (XEB). In a single cycle of error correction in the surface code, CZ gates are applied in layers which correspond to their position in a given stabilizer. Therefore, to more accurately represent the CZ performance in the surface code, we perform simultaneous XEB on pairs using their respective CZ layers. We summarize our CZ gate parameters and measured errors in Fig. S4. We also benchmark leak-

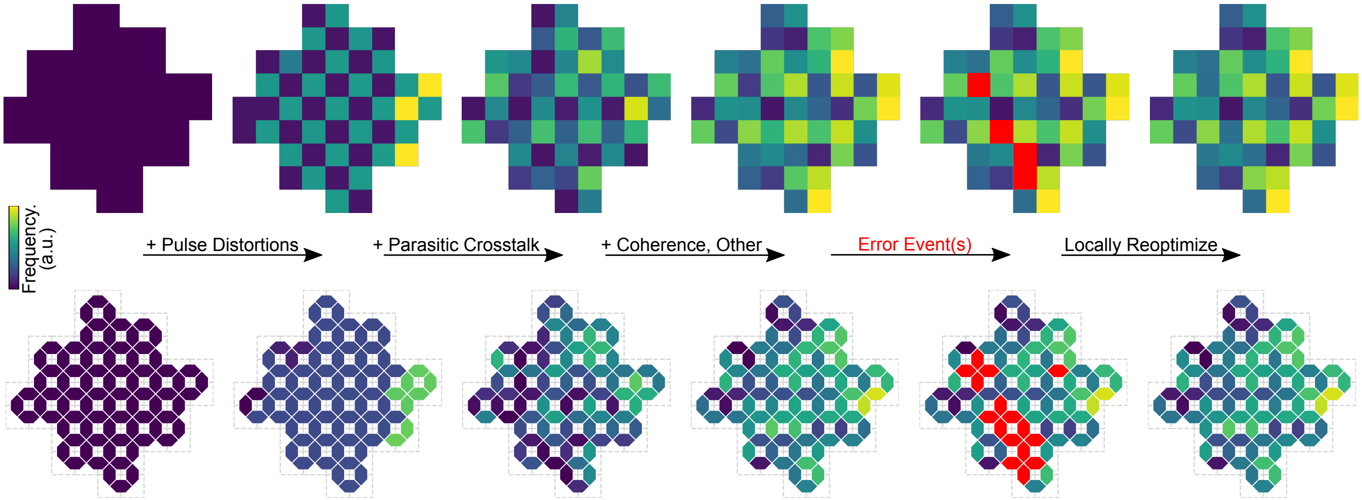

FIG. S2. Idle (top) and interaction (bottom) frequencies found by our Snake optimizer as error mechanisms are progressively enabled. Readout parameters are not shown. Starting from an unconstrained processor, enabling pulse distortions results in an idle checkerboard with nearly-degenerate interactions. This configuration minimizes frequency-sweeps during *CZ* gates. Enabling crosstalk results in idle and interaction patterns that resemble multi-layer checkerboards. This configuration minimizes frequency collisions over the surface-code. Finally, enabling dephasing, relaxation, and other error mechanisms leads to frequency configurations with no obvious structure. After optimization, random error events can render arbitrary gates unusable (red). In such scenarios, we locally reoptimize parameters and stitch solutions. Color scales were chosen to maximize contrast.

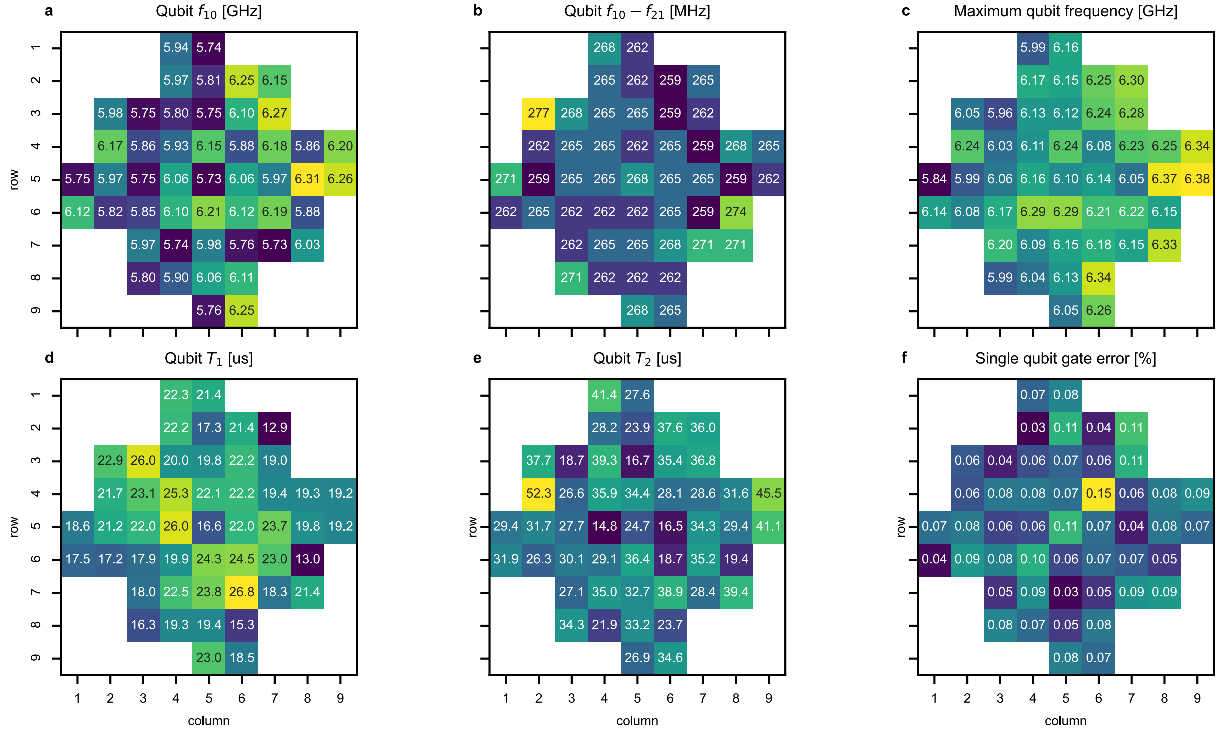

FIG. S3. **Heatmaps of various single qubit parameters. a,** Qubit transition frequency (ground to first excited state). **b,** Qubit anharmonicity at the operating frequency. **c,** Maximum achievable qubit frequencies. **d,** Qubit relaxation time. **e,** Qubit decoherence time as measured by CPMG ($T_{2,\mathrm{CPMG}}$ in the main text). **f,** Simultaneous single-qubit gate error.

age out of the computational subspace in our gates, and measure a mean leakage rate of 1×10^{-4} per gate.

E. Measurement and reset

The Sycamore processor uses frequency multiplexed dispersive readout. The chip has twelve readout lines, each of which includes six qubit-resonator pairs sharing a common bandpass Purcell filter, custom impedance matched parametric amplifier (IMPA) [64], and HEMT amplifier. The on- and off-chip hardware configuration is similar to that used in Ref. [31] with an important difference being the removal of the IR filter between the circulators and chip, which was done to improve the impedance match. The off-chip configuration for a single readout line is shown in Fig. S5. Each readout line has four ports: signal in, signal out, paramp pump, and paramp flux bias. The readout resonator is also used for qubit reset as described in Refs. [31, 44, 50]. In Fig. S6 (a-d) we summarize various parameters related to the resonators and the measurement chain. Feeding those parameters into models for different readout and reset error mechanisms, we optimize three readout parameters (qubit frequenecy during readout, readout pulse length, and readout pulse power) individually for each qubit. The total readout time is kept at 500 ns for each qubit; however for measure qubits we need the respective resonators to be empty before reset starts so we include time (equal to 500 ns minus the readout pulse length) for the resonators to ring down. This optimization is done using the Snake optimizer, see Sec. I B. For instance, we model errors due to finite signal-to-noise ratio, qubit relaxation during readout, $|10\rangle/|01\rangle$ and $|11\rangle/|02\rangle$ swapping between neighboring qubits, and qubit state transitions due to the resonator drive [65]. The optimized values are found in Fig. S6 (e-g). In the end, we benchmark readout by preparing and measuring a random set of qubit states, summarized in Fig. S6 (h). We measure an average error of 1.0% for $|0\rangle$, and 2.9% for $|1\rangle$.

F. Microwave crosstalk induced leakage mitigation

Leakage out of the computational subspace is an important error as it can lead to correlated errors during syndrome extraction. Microwave crosstalk is one mechanism that causes leakage during single qubit gates. In such a process, a small amount of the microwave signal from a "source" qubit couples to a "receiver" qubit which can then experience a crosstalk error. In particular, if the drive frequency of a source qubit is near $f_{1\to2}$ of a receiver qubit, a parasitic $|1\rangle \to |2\rangle$ leakage error can result on the receiver qubit. In general, source and receiver qubits do not need to be nearest neighbors.

To mitigate leakage, we apply a compensation pulse of equal amplitude and π out of phase with respect to the parasitic drive directly to the receiver qubit. Mathematically, we apply a signal $V'(t) = V(t)\, re^{i\theta}$ where V' is the applied compensation, and V is the source signal.

We calibrate the compensation parameters r and θ with a version of the Ramsey error filter pulse sequence, as shown in Fig. S7.a. For certain delay times t, this sequence amplifies coherent leakage induced by crosstalk, which facilitates this calibration. This amplification can be understood as a result of constructive interference between the leakage amplitudes induced by successive pulses.

Fig. S7.b shows representative data from the Ramsey error filter vs delay time t. This data is taken while driving all qubits simultaneously to minimize data acquisition time. In the following calibration steps, t is held fixed at the value that maximizes the leakage population $p2$. In fig. S7.c, we show the output of the Ramsey error filter at optimal t during pairwise operation for all possible pairs that include the receiver qubit. This data identifies the source of the parasitic drive.

Fig. S7.d shows $p2$ on the receiver qubit during pairwise operation with the dominant source qubit at optimal t vs the amplitude r and phase θ of the compensation tone. The red star indicates the optimal r and θ to mitigate crosstalk induced leakage.

G. Surface code experimental details

1. *Surface code circuits*

We present the surface code circuit in Fig. 1b (main text), focusing primarily on two qubits. The circuit generalises across the surface code, which we show here for clarity in Fig. S8. We also clarify the $ZXXZ$ stabilisers and how they relate to other stabilisers in Fig. S9. Considered in this $ZXXZ$ perspective, the logical operators in Fig. 1a alternate between local X and Z operators: as they are drawn, $X_L = XZXZX$ and $Z_L = ZXZXZ$.

In addition to individual-gate calibrations to track how qubit phases are affected by each gate, we add additional corrections within the surface code circuit, empirically optimized to minimize detection probabilities [44, 68]. These catch-all corrections allow us to mitigate the impact of a variety of noise sources which all effectively act as a single qubit phase shift. We place a "virtual Z^{α}" correction preceding each Hadamard gate, and then tune the value of α for each correction to minimize the detection rate of the detectors that the correction impacts.

The measure qubit case is simpler because they are reset to $|0\rangle$ to start each cycle, meaning that the initial Hadamard does not need a correction. As a result, each measure qubit only has a single phase correction per error correction cycle. Data qubits, which are assumed to be in a non-trivial state throughout, require two phase corrections per cycle. In case there are "warmup" effects, we give each measure qubit a special correction value for the first cycle, and then each uses another value for the rest of the cycles, while data qubit corrections have unique

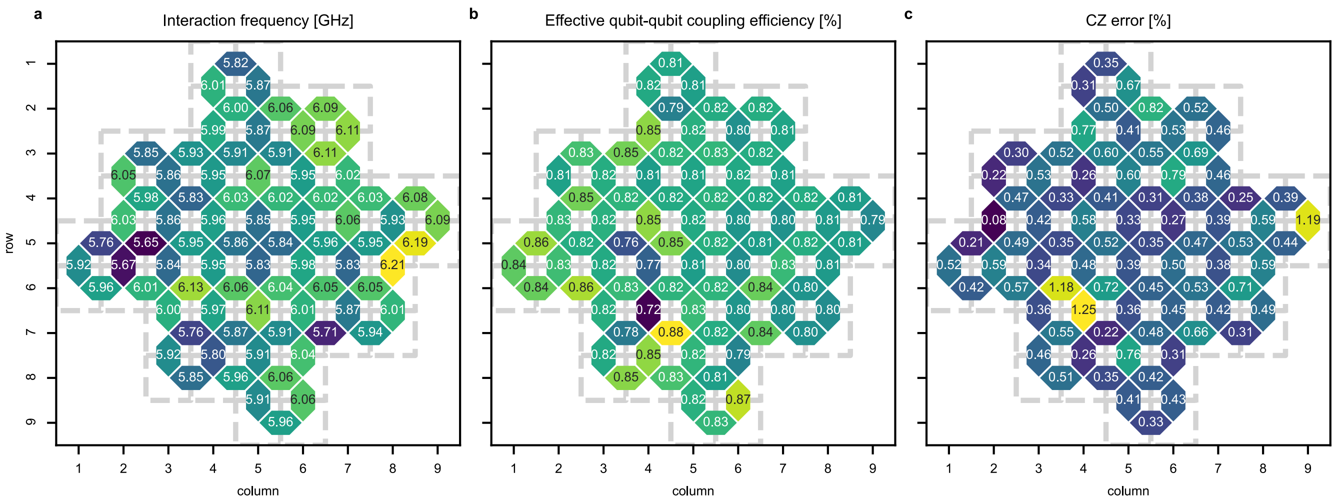

FIG. S4. **Heatmaps of various two qubit gate parameters. a,** Frequency where two coupled qubits interact to perform a CZ gate, specifically the average of the two qubits' f_{10} in the middle of the gate. **b,** The calibrated maximum coupling efficiency achieved between the two qubits during the CZ gate. The coupling efficiency is a unitless value, and the qubit qubit coupling is found by multiplying the coupling efficiency by the interaction frequency and dividing by 2. Note that these coupling efficiencies are inferred from a model of the circuit parameters for each coupler and pair of transmons. **c,** CZ gate error as measured using simultaneous cross entropy benchmarking, where the sets of CZ gates being executed simultaneously are the same as sets as in the surface code circuit.

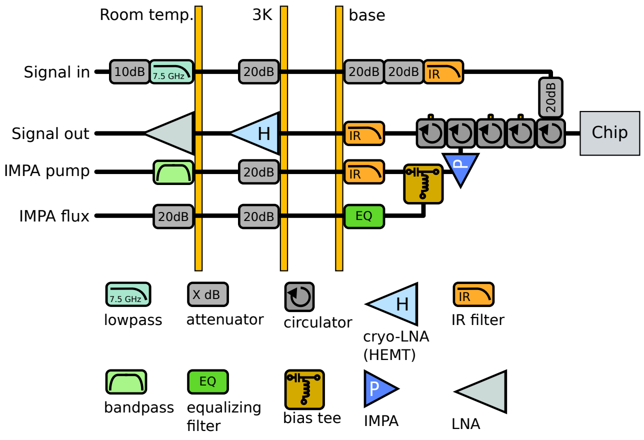

FIG. S5. Wiring diagram for the readout lines. The three vertical bars indicate temperature stages of the refrigerator.

values for the first cycle and the bulk cycle for each correction. Data qubits also have an additional phase correction in the last cycle, to correct for errors which occur right before logical measurement.

These corrections $\{\alpha\}$, typically small (< 0.1), are optimized using separate calibration experiments. In a given calibration experiment, we run the surface code circuit while sweeping the value of $\{\alpha\}$ on a specific correction, and record the detection probabilities for the specific detectors which are sensitive to phase errors at that circuit location. The value of $\{\alpha\}$ which minimizes the average detection probability is deemed the optimal value and used in primary quantum memory experiment.

Because different corrections are calibrated using different detectors, we can run many calibration experiments in parallel. We divide the corrections into ten groupings, where each grouping contains corrections which affect a unique set of detectors within that grouping. As an example, the measure qubit corrections only affect the detector on their own qubit line, so they can all be run in parallel, while corrections on a measure qubit and an adjacent data qubit may both impact the same detector, so they must be swept at different times to avoid

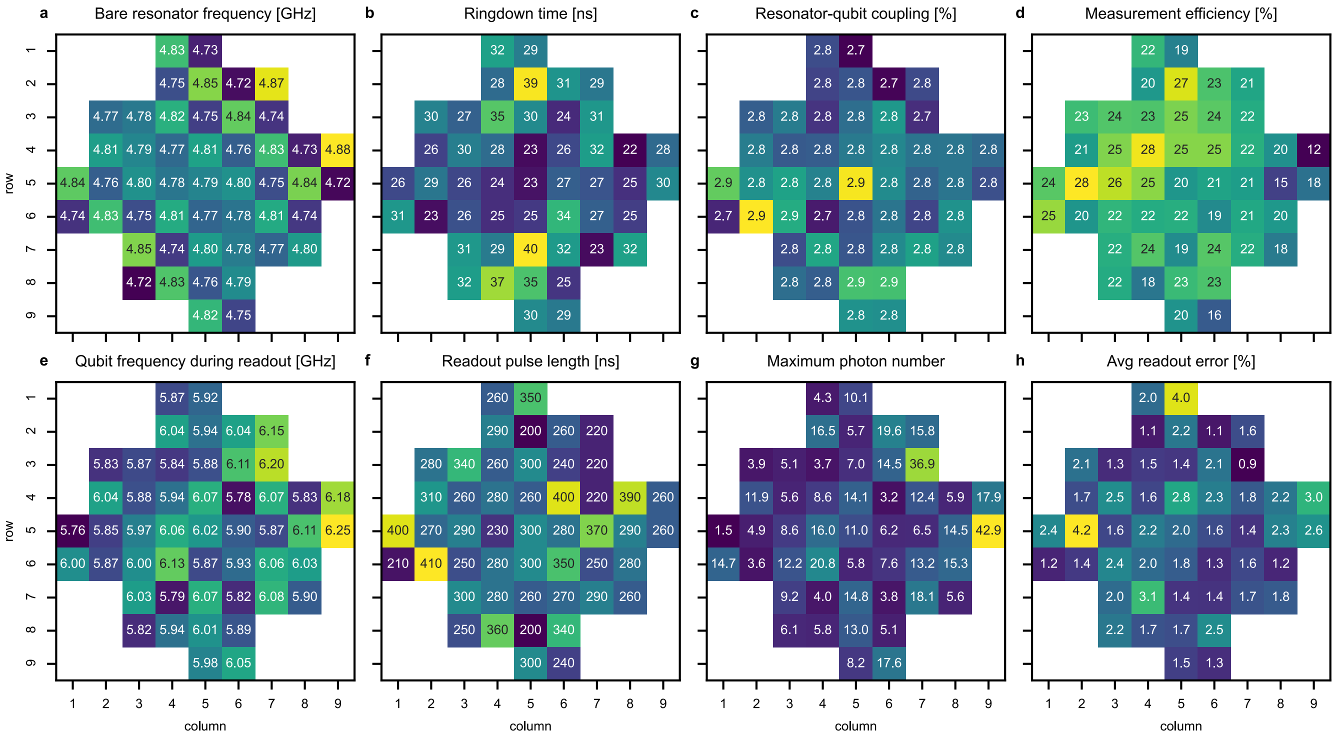

FIG. S6. **Heatmaps of various readout parameters.** **a,** Bare frequency of each readout resonator. **b,** Ringdown time (equal to $1/\kappa$) of each resonator. **c,** The unitless and frequency independent coupling efficiency between resonator and qubit. The coupling strength can be found by multiplying the coupling efficiency with the geometric mean of the qubit and resonator frequencies, and dividing by 2. **d,** Efficiency of the measurement chain close to the readout frequency. Measurement efficiency characterises the amount of information that can be learned, per measurement photon, about the qubit state, encapsulating all losses and added noise in the readout chain [66, 67]. **e,** Optimized qubit frequency during readout (in the absence of a readout drive). **f,** Optimized readout pulse lengths. **g,** Simulated maximum number of photons in the resonators during readout. The input readout pulse powers were calibrated using the AC-Stark shift. **h,** Measured average readout error over $|0\rangle$ and $|1\rangle$ when preparing a random set of qubit states across all 49 qubits.

having correlations obfuscate the outcome of the experiment. The groupings we use are based off the tiling of the surface code, and is independent of the size of the surface code logical qubit, so the number of experiments will remain fixed as we scale up the size of the surface code.

2. *Choice of distance-3 grids*

In total, there are 10 possible distance-3 grids whose footprint lies within the footprint of the distance-5 surface code grid. The four grids chosen were picked such that their overlap was minimized in order to reduce susceptibility to bias. There is still non-trivial overlap at the boundaries of the distance-3 grids, as well as the center qubit, which is included in all 4 of the smaller codes as seen in Fig. S10. Since the distance-3 codes are more susceptible to a single qubit with poor performance, the performance of the center qubit is essential to a fair measurement of $\Lambda_{3/5}$.

In Fig. S11, we show a dataset where the center qubit was experiencing excess phase errors due to a faulty calibration of the phase correction, dramatically impacting the performance of the distance-3 codes. In the inset, we can see that although this dataset would appear to show significant error suppression in the distance-5 code, it is clear that it is due to outlier performance. We can also see this by comparing to our models in Fig. 4 and observing that the logical error per cycle does not match where our models would expect such a crossover to occur. By looking at the detection probabilities in Fig. 2 of the main text, we can see that the performance around the center of the chip was within the standard range for the experiment presented, so we have no reason to believe that our measurement of $\Lambda_{3/5}$ was skewed.

3. *Order of experiments*

We interleave the experiments in Fig. 3 (main text). Specifically, we acquire one (number of cycles, basis, code) dataset at a time. Each dataset consists of 50000 total surface code runs (10 initial bitstring states times

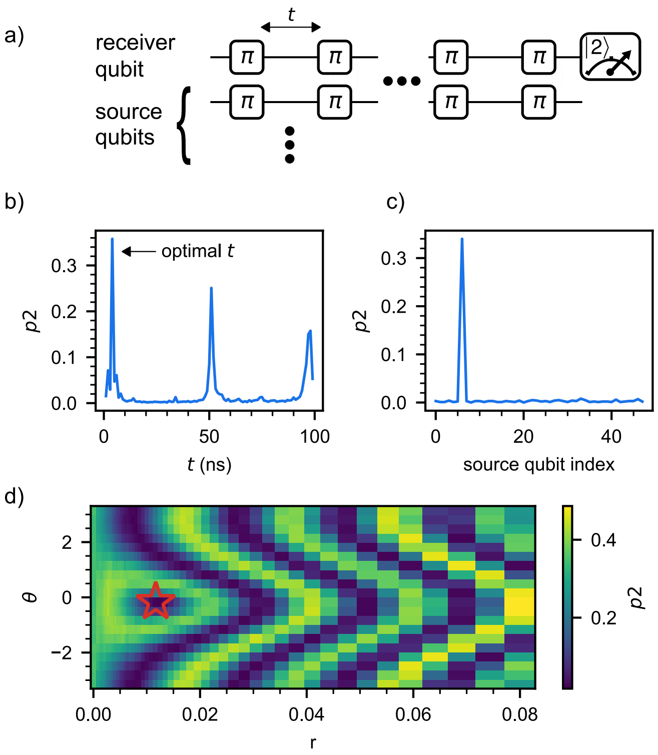

FIG. S7. Mitigation of microwave crosstalk induced leakage. **a,** The Ramsey error filter pulse sequence consists of π rotations separated by a variable delay t. We repeat this several times to amplify the coherent leakage, which we observe by direct measurement of $|2\rangle$. **b,** Leakage probability $p2$ vs t during the Ramsey error filter. This data is taken with all sources operating in parallel. The t that maximizes leakage is held fixed throughout the remainder of the calibration sequence. **c,** $p2$ vs source qubit during pairwise operation at optimal t. The peak identifies the dominant source. **d,** $p2$ after Ramsey error filter during pairwise operation with the dominant source vs r and θ, the magnitude and phase of the compensation tone used to null the crosstalk leakage. The red star indicates the optimal r and θ.

5000 repetitions).

We shuffle the order in which we acquire each (number of cycles) group of datasets. We begin with an extra 25-cycle experiment and then proceed with the other numbers of cycles in random order, in particular (25 (extra), 15, 17, 21, 13, 1, 19, 5, 23, 25, 7, 9, 3, 11).

Additionally, after each 5 cycles, we run a "frequency update" calibration on all the qubits. This is a simultaneous Ramsey experiment to quickly check the qubit frequency and update the flux bias values to tune the qubits to the desired frequencies if needed.

The pseudocode below describes the specific order in which the (number of cycles, basis, code) datasets were acquired. The total execution time is about 1.5 hours.

```
num_cycles = (25, 15, 17, ...)  # see text
codes = (
    d5,
    upper_left,
    lower_right,
    upper_right,
    lower_left,
)
for idx, n in enumerate(num_cycles):
    if should_run_frequency_update(idx):
        run_frequency_update()
    for basis in (Z, X):
        for code in codes:
            take_data(n, basis, code)
```

4. *Initial states*

As discussed in the main text, we use random bitstrings as the initial data qubit states. This avoids a situation where we start with a bias to more $|0\rangle$ measurement outcomes: if all the data qubits start in $|0\rangle$, about 3/4 of the measure qubits will see $|0\rangle$ in the first cycle, as opposed to 1/2, and there would be an asymmetry in measurement fidelity. This would artificially lower the error for the first several cycles as the code "warms up" to the steady state.

For the data in Fig. 3 (main text), we choose to initialize with 10 different bitstrings, 5 with each logical value. For N qubits, we generate 5 integers between 0 (inclusive) and 2^{N-1} (exclusive) and then interleave them with their N-bit bitwise complements (for odd surface code distance, bitwise complements have opposite logical values).

Specifically, the bit-packed decimal integers we use for distance-5 are (1497382, 32057049, 12984827, 20569604, 10981887, 22572544, 7363158, 26191273, 7264790, 26289641), and for distance-3, (22, 489, 198, 313, 167, 344, 112, 399, 110, 401).

The 25-bit representation of 1497382 is 0b0000101101101100100100110. Referring to Fig. S1, this is assigned to the data qubits in a big-endian fashion using the (row, column) coordinate system, with the most significant bit being (row=1, column=5), followed by (2, 4) and (2, 6) and ending with the least significant bit (9, 5). 1497382 is followed by its 25-bit complement, 32057049 or 0b1111010010010011011011001.

H. Repetition code experiment

A 50 cycle rep code is executed in 40 μs and there is a delay between shots of 8 μs, giving a total acquisition time of 24.5 seconds. Surveying 10 datasets from various repetition codes taken around the same time we observed an average of 2.3 cosmic ray hits per experiment, indicating that a cosmic ray hits our processor once every 10.6 seconds. This is in well agreement with our previous work in Ref. [59], and note that there are differences in experimental setup, including the processor size.

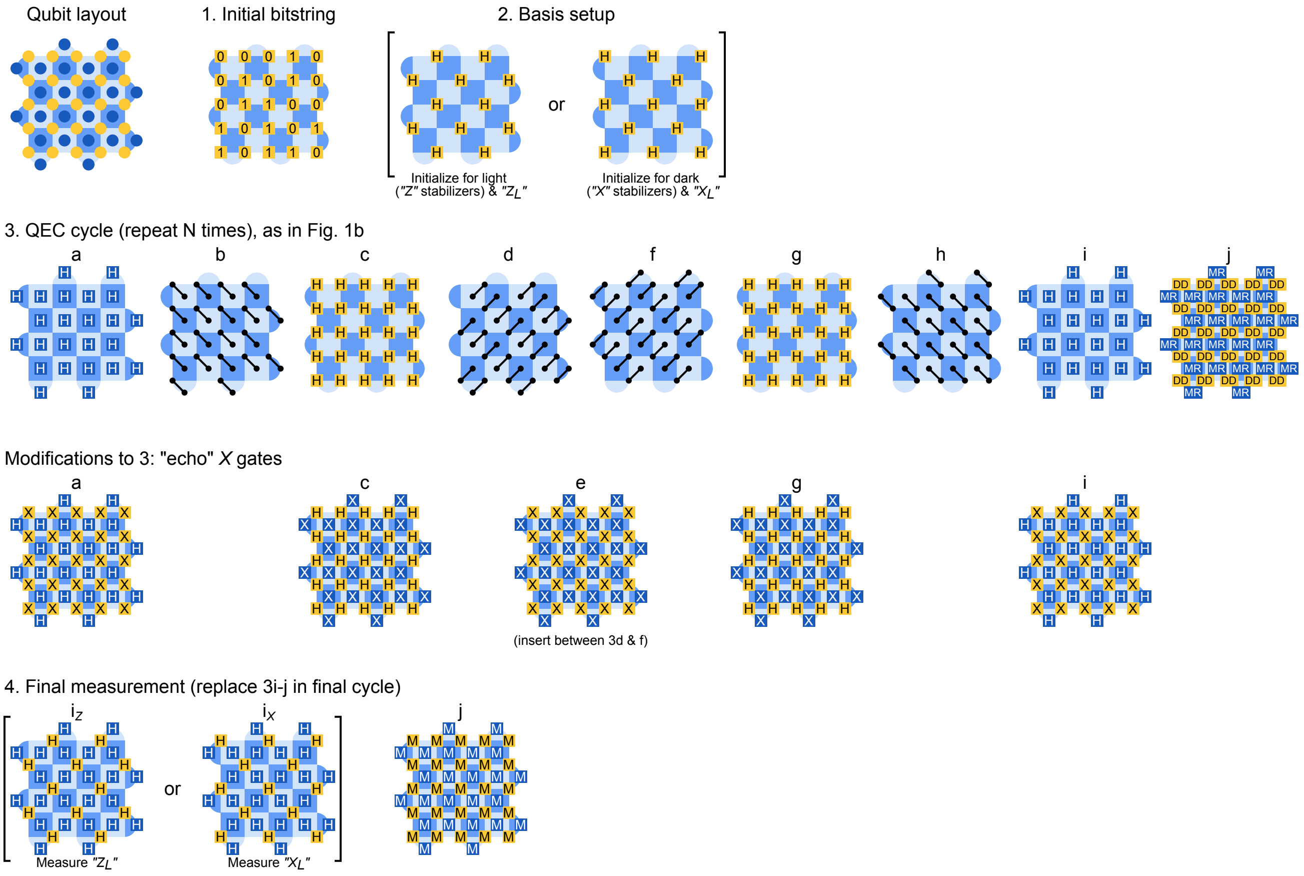

FIG. S8. **Distance-5 surface code circuits.** Expanded version of Fig. 1b (main text) showing the full sequence of operations. **1.** We reset all qubits to $|0\rangle$ and then prepare an initial bitstring state on the data qubits using X gates. **2.** We apply H (Hadamard) gates to some of the data qubits to convert the initial bitstring (eigenstate of all Z operators) into an eigenstate of half the $ZXXZ$ stabilisers, the half matching to logical operator of interest (X_L or Z_L) for the specific experiment. Note steps (1) and (2) could be combined into a single moment of Clifford gates, but for simplicity we execute them in two moments as shown. **3.** QEC cycle, as in Fig. 1b (main text), showing the explicit gate patterns. Note that although all stabilisers measure $ZXXZ$, the "X" stabilizers and "Z" stabilizers apply their CZs in a different order (specifically in 3d and f). This pattern is carefully designed to manage "hook" errors. As mentioned in the main text, we modify this gate sequence with additional "echo" or "dynamical decoupling" gates *within* the unitary part of the circuit. This amounts to adding X gates to the data qubits in 3a and 3i, to the measure qubits in 3c and 3g, and to all qubits in 3e (inserted between the middle two CZ moments). **4.** For the final measurement, we replace 3i-j with a different Hadamard pattern (transforming the data qubit state to the appropriate basis for logical measurement) and measure all qubits simultaneously. This final measurement is used for detectors for the penultimate cycle (measure qubit results) and the final cycle (data qubit results, converted to parities in the relevant basis).

To identify and remove the effect of the cosmic ray in the data presented in the main text, we apply a moving average filter with a window length of 30, identify peaks with mean detection probability $p_d > 0.2$, and throw out 100 shots before and 500 after. In total we discarded 741 continuous shots (36 ms) for this one event.

We illustrate the effect of this event on the logical performance in Fig. S12. At the peak of the spike in detection probability, the logical error probability is about 0.5 for all code distances d. Note these logical error probabilities are after 50 cycles, not logical error per cycle, indicating that the logical error per cycle is more than a few percent. The detection and logical error probabilities then decay back towards their pre-event levels, with the larger-distance codes recovering more quickly, as expected.

II. DECODING

A. Setting the prior distribution

Each decoder requires a prior distribution on the set of errors occurring in the device. However, there are

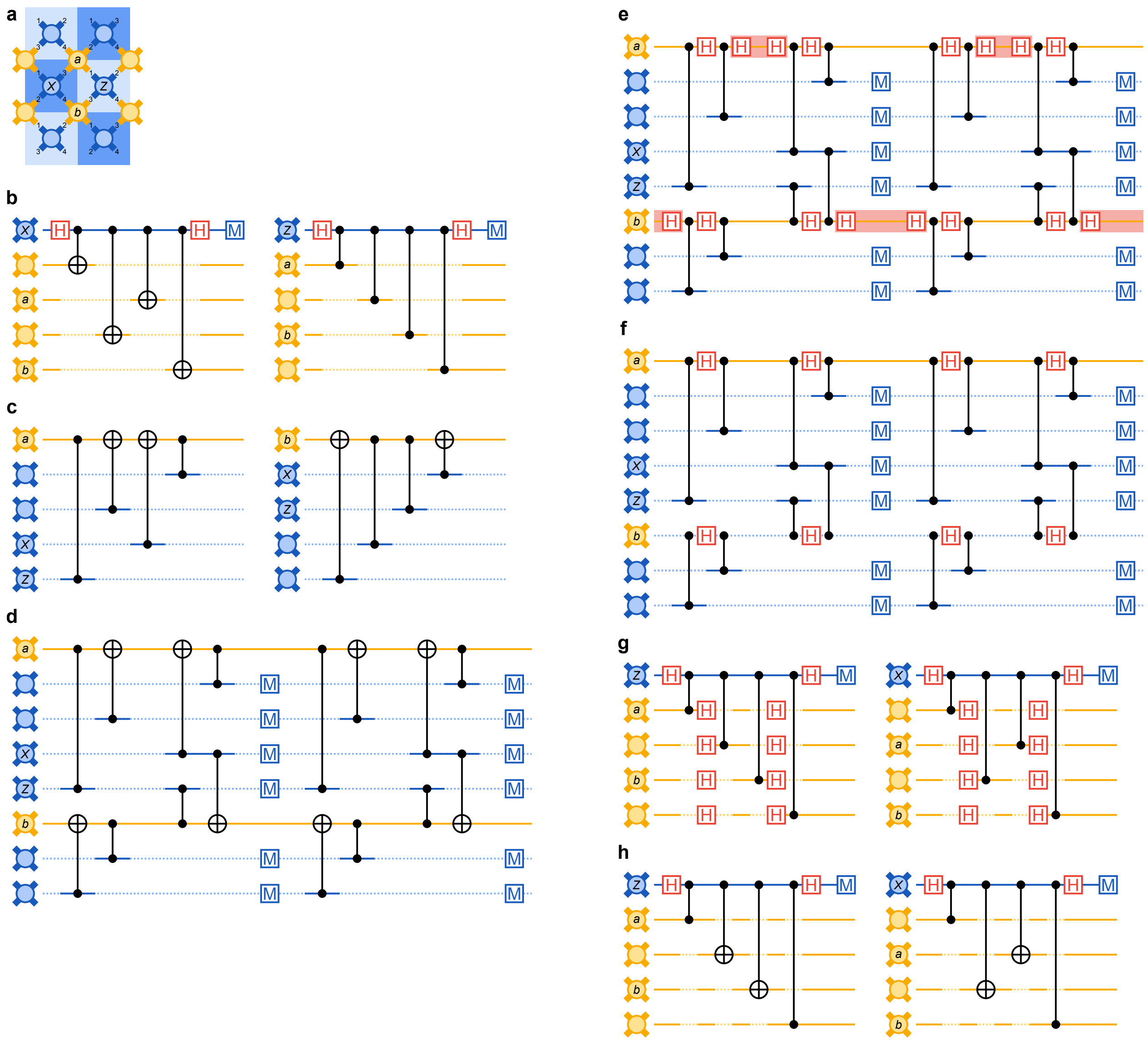

FIG. S9. **Circuit compilation from the standard surface code to *ZXXZ* stabilisers.** **a,** Schematic of a piece of the surface code with six data qubits (gold) and six measure qubits (blue). We indicate the chronological order of the *CZ* gates with the numbers 1-4 between each pair of qubits (also see Fig. S8). We focus on two data qubits a and b and two measure qubits X and Z. In the standard surface code, those qubits measure the operators XXXX and $ZZZZ$, respectively. **b,** Circuit compilations to measure XXXX (left) and $ZZZZ$ (right), with data qubits a and b indicated. Essentially, to measure an operator U on the data qubits, the measure qubit begins in $|0\rangle$, then performs a Hadamard (H), a controlled-U on the data qubits, another Hadamard, and is measured. **c,** The same circuits from **b** from the point of view of the data qubits a and b. **d,** The circuits from **c** combined together and repeated for two cycles. **e,** The circuit from **d** with CNOT compiled into *CZ* and Hadamard. This results in pairs of Hadamards with nothing in between, indicated with pink rectangles. **f,** The circuit from **e** with the redundant Hadamards removed. **g,** Single-cycle circuits from the point of view of the measure qubits, after the *CZ* compilation and Hadamard removal (otherwise similar to **b**). **h,** The same circuits from **g** recompiled to remove data qubit Hadamards and use CNOT instead. These can both be interpreted as "measuring $ZXXZ$."

many sets of errors which trigger the same set of detectors. Because our decoder only depends on the detection events themselves, we group error probabilities collectively into bins according to the set of detectors they activate. This is done using *Stim*'s detector error model functionality [69], which tracks Pauli errors to perform this binning automatically. Specifically, we use a simple depolarizing circuit noise model, derived from *Stim* circuit descriptions of the experimental circuits, to generate an initial set of bins of detectors and their collective probabilities. This information represents an initial error hypergraph - detectors are vertices, bins act as hyperedges

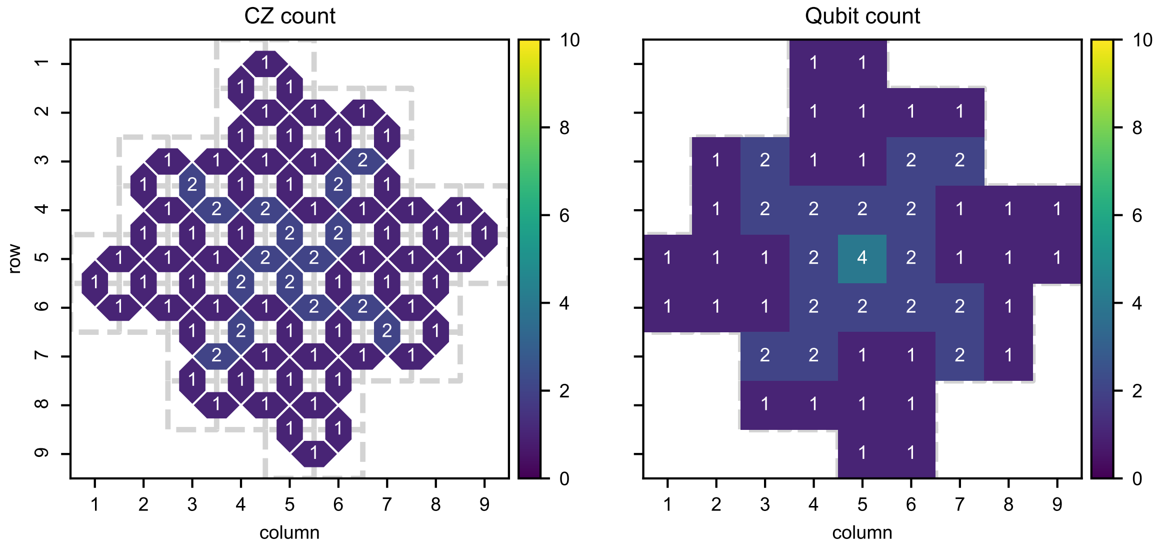

FIG. S10. Heatmaps of CZ and qubit involvements in the four distance-3 grids considered in this work.

connecting those detectors they activate, and hyperedge weights are defined from the collective error probabilities of the errors contained in each bin. Note that this is a slightly restricted model of decoding, and that more general models may be employed to increase the probability of success [58].

From here, we use a higher-order extension of the p_{ij} method to assign probabilities to this initial ansatz of hyperedges using device-level data [44, 51, 52]. We compute the highest-order correlations present in the standard depolarizing circuit model, which are weight-4, using the method described in Section VI C. Following that method, we compute lower-order correlations, subtracting the probabilities of higher-order correlations that contain them to avoid double counting. However, this procedure can underestimate probabilities due to over-subtracting when the detection event data exhibits additional correlations that are not captured in the initial ansatz [52]. This is most common in one- and two-body correlations, which may be contained in many three- or four-body correlations. To account for this, we enforce a simple averaged depolarizing T_1/T_2 noise probability floor (over the 500 ns measurement duration) for boundary edge one-body and spacelike two-body correlations to avoid unphysically small (or even negative) probabilities.

Because we use device-level data to calibrate the decoder, we must be careful not to fit the parameters of the decoder to the specific data that must be decoded. This can happen e.g. when optimizing minimum-weight perfect matching edge weights using gradient descent with the logical error per cycle as an objective function [68]. To avoid this issue, we decode an even subset of experimental trials by computing p_{ij} on the odd subset, and vice versa, similar to [68], and then average the two. This sidesteps the potential concern of having computed p_{ij} on the same data set as decoding (although in practice, we observe a negligible difference when decoding with p_{ij} computed from the decoded data set directly, as there is no optimization step). Without perfect statistics, this leads to a mild interdependence between the two individual decoding problems.

We validate the assumption that the interdependence is negligible by decoding half of a single experiment via this averaging method, and then decoding it again with p_{ij} computed from another quarter of the data independent from the half we decode. This ensures that each p_{ij} is given the same amount of statistics, so that the only additional difference between the two decoding pathways are statistical fluctuations. We perform an abbreviated (due to the computational cost of the tensor network) distance-3 decoding experiment using 5, 9, and 13 cycles with this method. With the tensor network decoder and belief-matching decoder, we observe a *relative* change of average logical error probability by 0.2% and 0.1%, respectively, when using the independently computed p_{ij} compared to averaging. These relatively weak dependencies of performance on the precise edge weights (rather than their general features) provide evidence that any interdependence between the two data sets due to statistical fluctuation is small.

For real-time decoding, it is also important that a decoder is robust to imperfections in the prior distribution e.g. caused by device drift. Although the tensor network decoder will likely be too slow to keep up with the throughput of a surface code processor, belief-matching is a promising candidate for building such a real-time decoder. To test belief-matching's sensitivity to an imperfect prior, we additionally use it to decode the experimental data with p_{ij} computed from earlier data generated by the device. We observe a very small increase in logi-

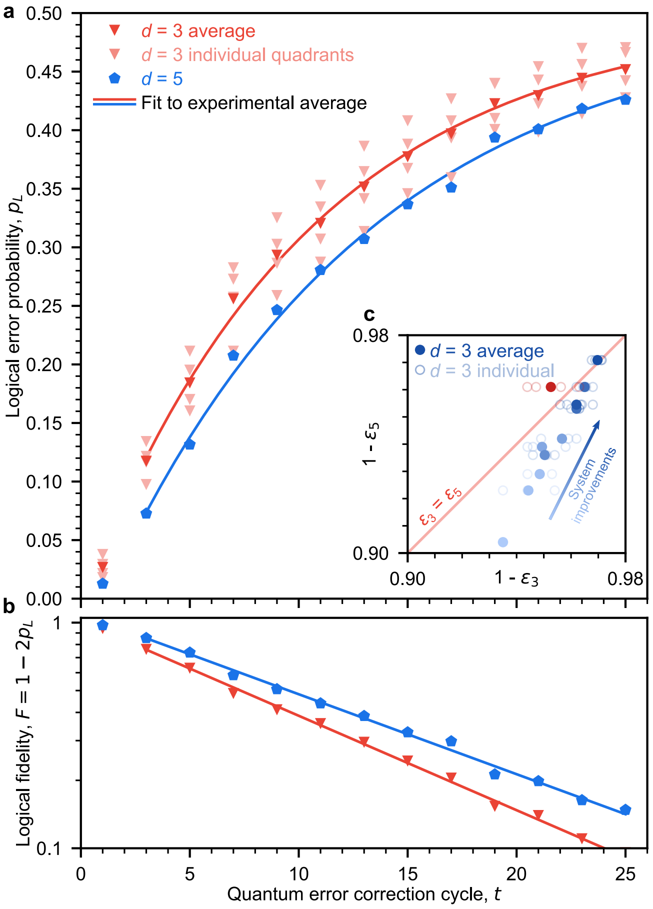

FIG. S11. Plots in the style of Fig. 3 in the main text, but for a dataset where the center qubit in the grid was experiencing performance issues. In **a** and **b**, we present the logical error probability versus cycle, while in the inset **c**, we present the same progression shown in the main text, but with this outlier set plotted in red.

cal error per cycle from 3.118% to 3.129% for distance-3 and 3.056% to 3.059% for distance-5. This robustness reinforces belief-matching as a promising candidate for real-time decoding.

B. Correlated minimum-weight perfect matching

For simulations involving larger scans through parameter space (e.g. for error budgeting), we employ a minimum-weight perfect matching decoder that uses a variant of the two-pass correlation strategy detailed in [70].

Correlated minimum-weight perfect matching first decomposes the error hypergraph into two error graphs by decomposing hyperedges into pairs of edges using *Stim* [69]. The initial pass of minimum-weight perfect matching is run, with edge weights given by $-\log(p)$ for edge probability p, to identify single edge matches. From these, we ascertain errors that are reasonably likely to have caused these single edge error paths. Then, using information about hyperedges containing these single edges, we can reweight the corresponding complementary edges in the alternate error graph to reflect the higher likelihood of having occurred. With this reweighted error graph - partly informed by detection events in the alternate error graph - we rerun minimum-weight perfect matching to obtain a more accurate solution. We refer the reader to [70] for more details.

Because the time cost of this decoder is essentially twice the time cost of minimum-weight perfect matching, we can leverage a fast custom matching engine to perform these larger scans quickly. Matching is also used to decode the repetition code, which does not require a two-pass strategy.

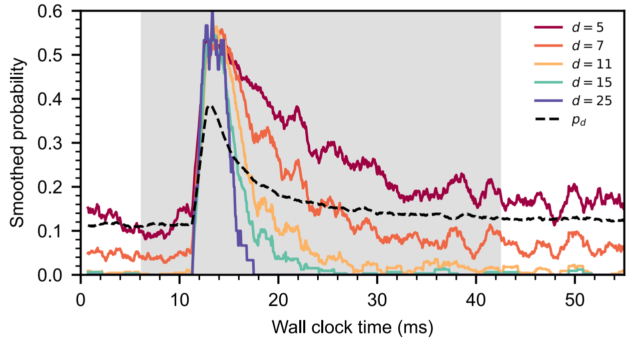

FIG. S12. **Logical performance of the repetition code during a high-energy event.** We plot the mean detection probability, p_d (averaging over the 25 measure qubits), which spikes upward at about 12 ms and then gradually decays (black dashed line). The color solid lines are the decoded logical error probabilities (evaluated after each 50-cycle experiment) for several code distances d ranging from 5 to 25. We smooth each trace using a moving average covering 30 experiments, about 1.5 ms. The light grey area indicates the shots that were discarded in the post-selected result described in the main text.

C. Belief-matching

The minimum-weight perfect matching (MWPM) decoder often used for surface codes is efficient, but only considers edge-like fault mechanisms in the error hypergraph, ignoring hyperedge fault mechanisms that include more than two detectors [34]. Like the correlated MWPM decoder, the belief-matching decoder exploits information about hyperedge fault mechanisms, but in a different way - by combining belief propagation (BP) with MWPM [53, 54]. In terms of efficiency, it has the same average and worst-case asymptotic running time as the conventional MWPM decoder [55].

In the first stage of belief-matching, BP is used to estimate the posterior marginal probability that each hyper-

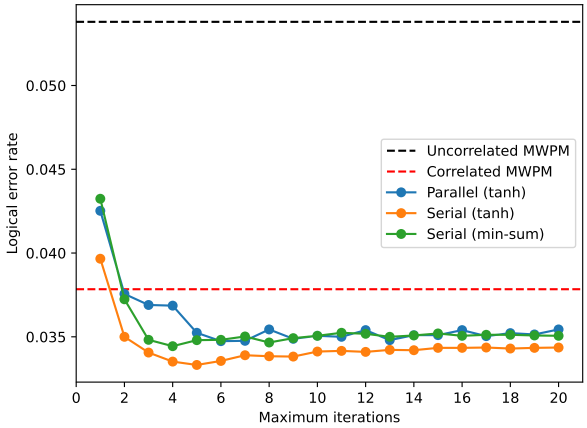

FIG. S13. Comparison of different variants of BP used as a subroutine in the belief-matching decoder for a distance-5 surface code with simulated circuit-level depolarising noise over 5 rounds of syndrome measurement. We compare a parallel and serial schedule using the tanh update rule, as well as a serial schedule using the min-sum approximation of BP with a scaling factor of 0.7. The x-axis shows the maximum number of iterations used for BP. The black and red dashed lines show the performance of an uncorrelated [34] and correlated [70] MWPM decoder, respectively. All decoders were run on the same simulated data set of 50,000 shots.

edge fault mechanism has occurred, based on the prior probability of each hyperedge in the error hypergraph, as well as the set of detection events that have occurred in the experiment. In the second stage, the error hypergraph is decomposed into two error graphs, using the BP posterior marginal probabilities to set the edge weights. These two error graphs are then decoded using a MWPM decoder [34]. We refer the reader to Ref. [53] for a more detailed description.

The main difference between our implementation of belief-matching and that of Ref. [53] is that we use a serial (rather than a parallel) schedule for BP and a maximum of 5 iterations. This choice of parameters is motivated by our analysis of different variants of BP in belief-matching for simulated surface code data, shown in S13. The serial schedule increases the rate of convergence of BP by roughly a factor of two [71–73], and we also find that it improves the accuracy of belief-matching relative to using a parallel schedule, which we attribute to the serial schedule mitigating against the problem of split-beliefs caused by quantum degeneracy [74]. We find that using more than 5 iterations for BP did not improve the accuracy of belief-matching for our simulations, and increasing the number of iterations can even degrade performance. We expect this is due to short loops in the Tanner graph bounding information spread beyond some local region [75] and leading to unreasonably confident log-likelihood ratios when the number of iterations is increased beyond the length of these short loops. Although we use the standard "tanh" rule for BP in our belief-matching implementation for the experiment, in S13 we also show the performance of belief-matching when using the min-sum approximation of BP. The min-sum algorithm is an approximation of BP that is more efficient to implement in hardware. Despite its relative simplicity, we find that the accuracy of the min-sum algorithm with a serial schedule is only slightly worse than that of BP using the tanh rule with the same serial schedule, and is comparable to that of the tanh rule with a parallel schedule. A review of the tanh rule and min-sum algorithm can be found in Ref. [76].

Practically, this decoder holds promise for scaling to the ~ 1 μs per round throughput required by a real-time superconducting quantum computer. In addition to using the min-sum approximation and a small number of iterations, the BP subroutine can be made even faster through the use of parallelisation. Furthermore, by using belief-find, which uses weighted union-find [77, 78] instead of MWPM for post-processing, further speedups might be achieved with only a very small reduction in accuracy [53].

D. Tensor network decoding

We implement a close-to-optimal decoder mapping the prior distribution defined in Sec. II A to a tensor network. Given a configuration of detection events and a choice of logical frame change, the contraction of this tensor network estimates its probability. It does so by summing the probabilities of all error configurations compatible with the set of detection events and logical frame change considered. This decoder guesses the more plausible logical frame change by comparing the likelihood of both outcomes. Unlike similar previous approaches [53, 56, 57], our protocol takes as input device level noise specified by p_{ij} correlations rather than gate-level noise.

The contraction complexity of the resulting tensor network grows exponentially in d^2, where d is the distance of the code. In practice, we contract it approximately as a matrix product state evolution with a finite maximum bond dimension, χ. The complexity of this approximate contraction grows as χ^3. In order to guarantee that the value of χ used achieves convergence, we study the logical error probability of a distance-5 Z-basis experiment over 25 cycles - the largest experiment run. We show in Fig. S14 that the logical error probability already converges at $\chi = 30$, which we use to decode. Our implementation of this decoder uses the tensor network library quimb as a backend [79]. Note that the tensor network decoder is many orders-of-magnitude slower than belief-matching and correlated MWPM - decoding all of the experiments was done on a cluster and took tens of CPU years in total.

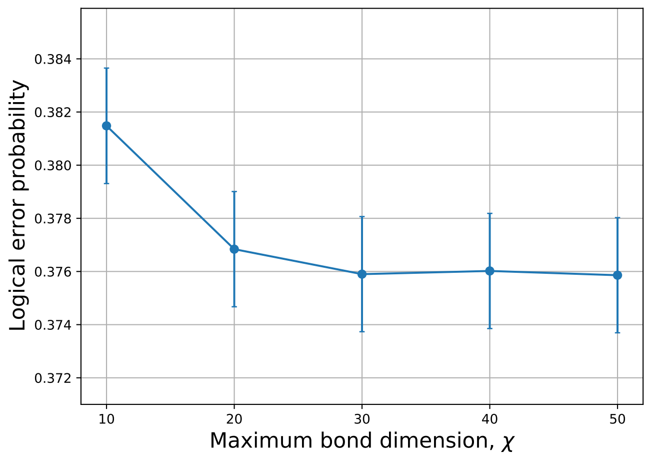

FIG. S14. Logical error probability of a distance-5, Z-basis, 25-cycle experiment with 50,000 shots as a function of the maximum bond dimension χ used in the tensor network contraction. Error bars denote standard error of the mean. We observe that the logical error probability stabilizes at $\chi = 30$.

III. FITTING ERROR VERSUS CYCLES TO EXTRACT LOGICAL ERROR PER CYCLE

There are many ways to fit the logical error probabilities versus cycles to extract the logical error per cycle, which can yield slightly different results. In this section we lay out the procedure followed for this work.

We start with logical fidelities $F_\mathrm{L}(t) = 1-2P_\mathrm{err}(t)$ for t the number of cycles. As discussed in the main text, the first cycle disproportionately favors the distance-5 code, and we thus discard it from our fits. Our goal is to find some logical error per cycle ε such that

$$\begin{aligned} P_\mathrm{err}(t+1) &= (1-\varepsilon)P_\mathrm{err}(t) + \varepsilon\big(1-P_\mathrm{err}(t)\big) \\ &= \varepsilon + (1-2\varepsilon)P_\mathrm{err}(t) \end{aligned} \tag{1}$$

which corresponds to exponential decay in the fidelity:

$$F_\mathrm{L}(t+1) = (1-2\varepsilon)F_\mathrm{L}(t). \tag{2}$$

To do this, we first take our $F_\mathrm{L}(t)$ data and append error bars from a binomial distribution, meaning that the variance at each point is given by

$$\sigma^2 = \frac{P_\mathrm{err}(t)\big(1-P_\mathrm{err}(t)\big)}{N} \tag{3}$$

for N the number of samples. We then take the base-10 logarithm of each of these points, transforming the error bars as appropriate, before fitting to simple two-parameter linear fit using least squares, weighting the squared errors by the transformed variances to approximate a maximum-likelihood estimate. We then exponentiate this linear fit in order to get the fit in the original coordinates. In Fig. S15 we show this process applied to the Z-basis distance-5 dataset presented in the main text. When combining data over multiple logical bases, or the different distance-3 datasets, this process is done on each dataset separately, and the final ε's are averaged. The different random initial states for a single code and logical basis are considered to be one dataset.

We additionally quantify out-of-model errors (such as leakage and device drift) by comparing the residuals of the fit to the residuals we would expect from binomial sampling errors alone. As the residuals are roughly three times larger than what we expect from sampling noise alone, we scale up the uncertainty calculated for sampling noise by this factor to account for the additional variance in our estimates from these out-of-model factors. As a final conservative measure, we upper bound our uncertainty by combining uncertainties from individual fits (from different initial-state bases and different distance-3 configurations) via averaging, which accounts for the possibility that these out-of-model errors might be correlated across different subsets of experiments.

As a check that this can appropriately account for additional errors, we also fit an extended model in which the residuals in our fits come primarily from fluctuations in the logical error per cycle ε itself instead of binomial sampling noise. Concretely, we model the measured logical fidelity at cycle t as $F_\mathrm{L}(t) = A[1-2\varepsilon(t)]^t$, where A is a constant factor accounting for preparation and measurement errors and $\varepsilon(t)$ is a normally distributed random variable $\varepsilon(t) \sim \mathcal{N}(\mu_\varepsilon, \sigma_\varepsilon^2)$, sampled once for each t. We fit both the mean and the variance, where the weights for the least-squares fit in this model come from these fluctuations in ε, as do the error bars on our estimate of the mean logical error per cycle. This model results in near identical separation between the logical error per cycle for distance-3 and distance-5, as measured in standard deviations of the estimate. The results of this fit are illustrated in Fig. S16 for all potential subintervals, demonstrating the robustness of our findings to out-of-model errors and fitting choices.

IV. SIMULATION OF LOGICAL MEMORY EXPERIMENT

We consider multiple simulation strategies for the logical memory experiment. The simplest approach, labeled "Pauli" in the main text, is implemented as a standard Pauli frame simulation. This simulation is based on the Stim open source library [69]. The associated error model corresponds to adding one and two-qubit depolarizing channels after each device operation, matched to experimentally benchmarked fidelities. The typical fidelities observed are summarized in Fig 1.C of the main text. Mean values for the distance-5 experiment can be read off directly from the first rows of Table I (excluding leakage and crosstalk).

A more sophisticated simulation approach is labeled "Pauli+". This is still a classical simulation in the sense that the cost grows polynomially in the number of qubits,

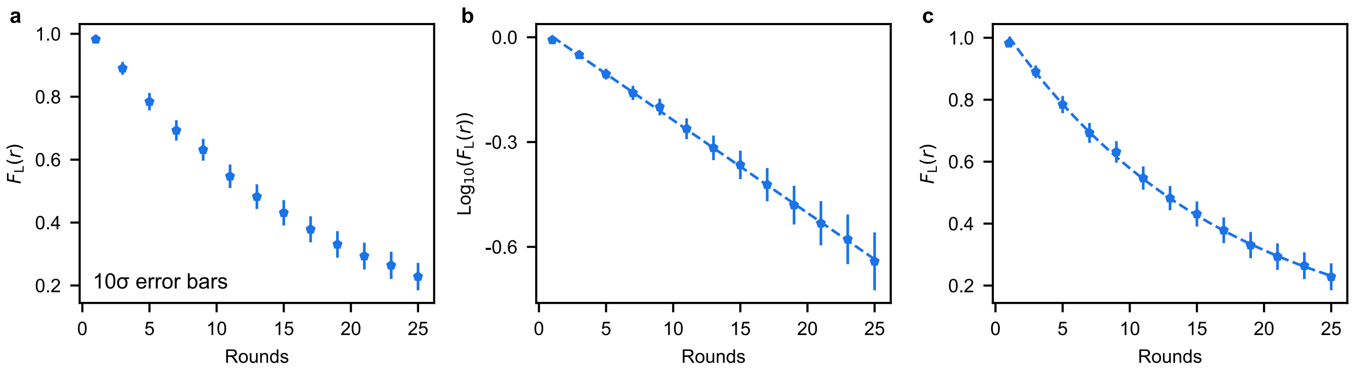

FIG. S15. A figure demonstrating our fitting procedure on the Z-basis distance-5 data from the run presented in the main text, decoded with the tensor network decoder. **a,** The logical fidelity data as a function of cycles, with error bars pulled from the binomial distribution (in this figure, all error bars shown at 10σ to help with visibility.) **b,** The same data after a base-10 logarithm has been applied to it, with the error bars transformed appropriately, along with a two-parameter linear fit. **c,** The data transformed back into the original coordinates, along with the transformed fit.

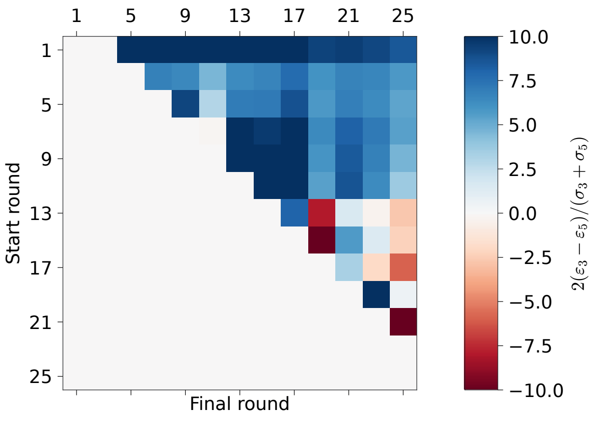

FIG. S16. Results of fitting to an extended model with fluctuating ε. Fits are performed for all subintervals of cycles containing at least three points, and the result of the fit is presented as the difference in logical error per cycle between distance-3 and distance-5 measured in the average of the uncertainties of logical error per cycle for each distance given the extended model. Fits starting from cycle 1 clearly favor distance-5 disproportionately, and are not used for our quoted results. Fits starting from cycles 3–11 show consistent significant separation between the estimates for logical error per cycle. The fit from cycles 3–25, the range used for our quoted logical error per cycle, shows a separation of 5.7σ between distance-3 and distance-5, which is consistent with the 5.8σ separation we get from our original fits when scaling our error bars by the excess residual factor.

though its performance is not as heavily optimized. The advantage of this simulation is that it explicitly accounts for correlated errors such as leakage of the qubit state outside the computational subspace and parallel CZ gate crosstalk.

Finally, we do a "brute force" quantum simulation of the distance-3 experiment, as enabled by the *kraus_ sim* library (Sec. IV B 1). This is a quantum trajectories simulation [80, 81], meaning that each experimental sample corresponds to propagating the noisy evolution of the full quantum state vector. Unlike the previous approaches, the quantum simulation is "exact" in the sense that all noise is modeled using explicit quantum channels derived from physical considerations. The previous approaches must represent noise using Pauli channels, which can be derived from the more general quantum channels through approximations (detailed below). While the quantum simulation is not scalable to large code distances, we can use it to validate the approximations required for the Pauli+ simulator (see Sec. IV C).

Below we outline the general procedure to simulate the logical memory experiment.

1. The "ideal" experiment is represented as a noiseless quantum circuit. Each layer of gate, idle, measure, or reset operations is encoded as a `Circuit` object of the *Cirq* open source library [81]. Lists of simultaneous operations are organized into `Moment` objects.

2. The "ideal" circuit is mapped to a "noisy" circuit. This corresponds to inserting additional moments into the circuit composed of operations representing noise. (The noise models used are described in Sec. IV A.) Each noise operation is a quantum channel encoded using Kraus operators. Such a representation is sufficient for the *kraus_ sim* quantum trajectories simulator. The noisy circuit processing is also implemented using the *kraus_ sim* library.

3. Additional processing of the noise is required for the "Pauli+" simulator. As detailed in Sec. IV B 3, we apply a generalization of the Pauli Twirling Approximation [82] to construct Generalized Pauli Channels (GPCs) capable of including leakage.

4. The chosen simulator generates measurement samples of the experiment represented by the noisy circuit. The initial data qubit states are matched to those used in experiment, and the results are then processed in the same way as experimental measurement data.

In the following sections, we discuss details of the simulated noise models, the *kraus_ sim* and Pauli+ simulators, as well as the Generalized Pauli Twirling Approximation. We conclude with a comparison of results between the quantum (*kraus_ sim*) and classical (Pauli+) simulations of a distance-3 experiment.

A. Noise models

Below we give a brief summary of all noise models included in our simulations. These models are accounted for using Kraus operators, which define quantum channels applied to a qubit after its respective gate or idling operations. In the Pauli+ simulation, these channels are first converted into corresponding Generalized Pauli Channels (GPCs) so that the noisy experiment is amenable to efficient (classical) simulation.

1. Decay, dephasing, and leakage heating

We consider the effects of qubit decay, white noise dephasing, and passive leakage to state $|2\rangle$ for an idling transmon qubit. We assume decay into a large, zero temperature environment with characteristic decay time T_1. Each transmon qubit (both measure and data) couples to the environment through its charge operator q. This induces a decay (in the energy eigenbasis) through the $|j\rangle\langle j+1|$ elements of the charge operator, which we assume scale as $q_{j,j+1} \propto \sqrt{j+1}$. Fermi's Golden rule predicts a $j+1 \to j$ decay rate proportional to $|q_{j+1,j}|^2 \propto (j+1)$, so we expect the state $|2\rangle$ to decay twice as fast as state $|1\rangle$. Similarly, one can derive dephasing as arising from a weak measurement of the number operator n, with information leaking at a rate $1/T_\phi$. Finally, we include a phenomenological leakage heating rate $\Gamma_{1\to 2}$ described by the Linbladian operator $\sqrt{\Gamma_{1\to 2}}|2\rangle\langle 1|$. The dynamics of an idle transmon in the presence of these processes is described through the master equation,

$$\begin{aligned}
\partial_t \rho &= -\frac{i}{\hbar}[H^{\text{idle}}, \rho] + \mathcal{L}[\rho] \\
&= -\frac{i}{\hbar}[H^{\text{idle}}, \rho] \\
&\quad + \frac{1}{T_1}\left(a\,\rho\,a^\dagger - \frac{1}{2}\{n, \rho\}\right) + \frac{2}{T_\phi}\left(n\rho\, n - \frac{1}{2}\{n^2, \rho\}\right) \\
&\quad + \Gamma_{1\to 2}\left(|2\rangle\langle 1|\rho|1\rangle\langle 2| - \frac{1}{2}\{|1\rangle\langle 1|, \rho\}\right), \qquad (4) \\
a &= \sum_{j\geq 0} \sqrt{j+1}|j\rangle\langle j+1|, \\
n &= \sum_{j\geq 0} j|j\rangle\langle j| = a^\dagger a,
\end{aligned}$$

where H^{idle} denotes the Hamiltonian of the idling transmon (diagonal in the standard basis $|j\rangle$). The values for T_1 and T_ϕ used in our simulations are both qubit specific. They are extracted from experimental decay and CPMG data. For $\Gamma_{1\to 2}$ we use a homogeneous value of $1/(700\mu\text{s})$, which is in agreement with typical numbers for both our current device and past experiments [83].

We use the dissipative dynamics in Eq. (4) to extract corresponding discrete quantum noise channels. These are applied on each qubit following every idle or gate operation. For an operation of duration t, evolution under the master equation can be approximately decomposed as $\mathcal{E} \circ \hat{U}_0$, where $\hat{U}_0$ represents the ideal unitary evolution and

$$\mathcal{E} = \exp(t\mathcal{L}) \qquad (5)$$

represents the effect of decoherence. We solve for $\mathcal{E}$ by representing $\mathcal{L}$ as a linear operator and doing the direct matrix exponentiation; this ignores a correction due to weak non-linearity of the spectrum of H^{idle}. From this we follow the standard prescription to extract the Kraus operators of $\mathcal{E}$. These can be extracted directly from any square root of the Choi matrix [84, 85], $\sum_{i,j}(|i\rangle\langle j|) \otimes \mathcal{E}(|i\rangle\langle j|) = XX^\dagger$.

2. Readout and reset error

Readout error is assumed to be a classical process. Accordingly, our simulations in fact implement perfect readout in the standard basis (including leaked states $|2\rangle$ and $|3\rangle$) and errors are added using a Markov transition matrix in post-processing. To populate this matrix we use the calibrated readout fidelities in the device. Measure qubit readout fidelities are calibrated during parallel readout of all measure qubits, while for data qubits we use fidelities calibrated during parallel readout of *all* qubits. Since the experiment does not actually implement readout that distinguishes the leaked states, we assume that both state $|2\rangle$ and $|3\rangle$ are read out as $|0\rangle$ or $|1\rangle$ with equal probability. Additionally, we account for

measure qubit reset error by adding this probability to the average readout error of the measure qubits. This is justified as a reset error on a measure qubit at the beginning of an error correction cycle has the same effect as a measurement error at the end of the cycle.

3. *Leakage*

Leakage outside the computational subspace is caused by three mechanisms included in our models.

1. Heating: Passive heating from state $|1\rangle$ to $|2\rangle$, as included in the first error model (Sec. IV A 1).
2. CZ gate dephasing: Since our CZ gates are diabatic [16] (implemented as one cycle trip swap $|11\rangle \rightarrow |02\rangle \rightarrow |11\rangle$), dephasing processes may cause transitions between states $|11\rangle$ and $|02\rangle$. To model this process, after each CZ gate we include a channel with Kraus operators

$$K_1 = \sqrt{p_t}\,(|02\rangle\langle 11| + |11\rangle\langle 02|)$$
$$K_0 = \sqrt{I - K_1^\dagger K_1}\,. \quad (6)$$

   The value $p_t = 8 \times 10^{-4}$ used matches the median value observed in the characterization of CZ gates. To exclude parallel gate crosstalk effects, leakage in each CZ gate was measured while all neighboring qubits were held idle. Note that during implementation of the diabatic CZ gate, only the higher idle frequency qubit occupies the $|2\rangle$ state. Therefore this is the only qubit with considerable dephasing-induced leakage. The qubit ordering used to specify the transition Kraus operator K_1 reflects this [44].
3. CZ crosstalk: As discussed below, coupling crosstalk between independent CZ pairs or idling qubits may lead to leakage transitions.

Along with affecting measurement outcomes, we also account for the effect of leakage on our CZ gates. This is captured by two processes:

1. Controlled phases: When one of two qubits is in a leaked state $|j\rangle$ during the CZ gate, the applied Z phase on the other, ϕ_j, will typically not be 0 or π. When the non-leaked qubit is a measure qubit, this will have a randomizing effect on the observed stabilizer measurement [86]. Under the twirling approximation used in the Pauli+ simulation, this effect is mapped to a σ_z Pauli channel applied conditionally on the non-leaked qubit.
2. Higher level transitions: The physical implementation of the diabatic CZ gate leads to resonances between higher energy states (e.g. $|22\rangle$ and $|13\rangle$). Accordingly, this leads to coherent transitions between these states and effective transport of leakage between measure and data qubits [50].

In order to accurately capture the above processes, we numerically calibrated and simulated the CZ gate matching the experimental qubit and coupler control schedules. Each transmon qubit was represented using the five lowest energy levels, and the coupler was represented using four. Qubit idle frequencies (~ 6 GHz), hold frequencies, and gate durations were matched to experimental values, while the coupler frequency and qubit detuning at hold were the only optimized parameters [16]. A Gaussian filter with 1 ns rise time was applied to all frequency waveforms. The observed values for ϕ_j, as well as the transition amplitudes (specifically between states $|22\rangle \Longleftrightarrow |13\rangle$ and $|21\rangle \leftrightarrow |03\rangle$) were extracted. These effects are then combined into a single unitary operator $U_{leakage}$ that is applied following the "ideal" CZ unitary. The ideal unitary acts as a perfect CZ in the computational subspace and as a local diagonal operator on the higher energy states, with phases determined by the gate time and qubit nonlinearities.

4. *Crosstalk*

We model coupling crosstalk between qubits undergoing parallel CZ or idling operations. This crosstalk error is caused by two effects. First, the *inactive* couplers (i.e those not implementing a CZ gate) used to mediate the qubit-qubit couplings at idle are unable to cancel all effective couplings over all fixed excitation subspaces [87]. Second, diagonal capacitive couplings (e.g., between next-nearest-neighbor qubits separated by a single horizontal and vertical displacement on the square grid) are also present throughout the device, with $|01\rangle \leftrightarrow |10\rangle$ swap rates on the order of 200 kHz. To account for both of these effects, we consider all pairs of simultaneous gates (either single qubit idle or two-qubit CZ). For each pair of gates containing a diagonal or nearest neighbor, we simulate the parallel operation of both gates in the lab frame (while including the undesired couplings above). The full 3- or 4- qubit unitary U_{lab} is then re-expressed in the "interaction picture"

$$U_{lab} U_0^\dagger = U_I \quad (7)$$

where U_0 represents the "ideal" evolution in the absence of the unwanted couplings. Note that the ordering of operations is swapped compared to the typical interaction picture definition $U_{lab} = U_0 U_I$. This is to retain consistency with the other noise models, for which we append error channels after (instead of before) each ideal gate operation. This ideal evolution can be computed as a tensor product of unitaries acting on the two subsystems involved in each gate. Note that the simulations are carried out using 4-level qubits, so that leakage-related effects are explicitly included. The U_I unitaries for all pairs of neighboring gates are included as error channels in the representation of the noisy circuit. We note that the dominant error mechanisms stem from unwanted

$|01\rangle \leftrightarrow |10\rangle$ swapping between neighboring qubits (undergoing separate CZ / idle gates). This swapping is highly sensitive to unwanted resonances arising during parallel operation.

5. *Depolarizing errors*

While the models above account for a majority of errors in the experiment, they typically under-predict the experimentally calibrated decay of fidelity in benchmarking experiments. To compensate for this missing error, we append additional depolarizing errors after each gate. In all cases, the fidelity decay curves are converted to an inferred Pauli error: we then find the depolarizing channel that would produce the observed same decay curve and extract its Pauli error. This error is compared to the sum of Pauli errors accumulated over the error models described above. (We assume that for independent error sources, this should match the total Pauli error to leading order [88].). The effect of leakage transitions on the Pauli error are explicitly accounted for as in Ref. [89].

There are multiple possible factors leading to the discrepancy between the implemented error models and the benchmarking experiments. Single qubit gates are characterized using parallel randomized benchmarking [30], while CZ gates are characterized with parallel cross-entropy benchmarking (XEB) [31]. The data qubit idles (during measure qubit measurement and reset) are characterized using interleaved single qubit XEB. In all of these cases, effects such as microwave or DC (flux) control crosstalk may contribute to component fidelity decays. Effects involving other quantum degrees of freedom may also be involved, such as the coupler transitioning to its first excited state [87] or coherent TLS that come close in frequency to a qubit. Drifts in control electronics or TLS frequencies between device calibration and collection of experimental data may also be involved. Measuring and mitigating these (and other) effects are an active area of research.

B. Simulator details

1. *kraus_sim - noisy circuit compiler and quantum simulator*

The *kraus_sim* library serves as an intermediate layer between the *Cirq* (used to represent the circuit) and *qsim* (used for high-performance qubit state evolution) [31, 90] libraries. At the interface to *Cirq*, it processes the ideal circuit to generate the noisy circuit (as described in Sec. IV A). These noisy circuits are then consumed by *kraus_sim*'s quantum trajectories simulator. A single quantum trajectory is equivalent to consecutively sampling the Kraus operators of each applied quantum channel using Born's rule for POVMs [91], then applying the Kraus operator to the state vector and normalizing the result [24, 81]. Below, we highlight unique features of the library, which necessitate a custom simulator backend based on the the *qsim* library.

The *kraus_sim* library encodes all operations (both gates and error channels) using Kraus operators, each of which is represented as a multi-dimensional tensor. While typical Kraus operators are "square" matrices, we allow these tensors to have an arbitrary shape. This means that operations can be defined which "create" or "destroy" degrees of freedom in the system. Accordingly, in many cases it is possible to reorder the application of commuting operations to greatly speed up calculations by decreasing the size of the state vector. Specifically, the structure of the surface code and bit flip code circuits means that operations can be ordered so that a "destructive" measurement of a single measure qubit is following by a "creative" reset of another measure qubit [92]. Thus, only a single measure qubit ever needs to be represented in memory. This allows for noisy simulations of up to a distance-5 surface code (25 data + 24 measure qubits) while keeping at most 25+1 qubits in memory. However, this technique is not possible in the presence of 3- and 4-local crosstalk errors, since if two measure qubits are affected by such error they must both exist concurrently in the simulation. We therefore focus only on distance-3 simulations (requiring only 9+8=17 qubits in memory).

kraus_sim also implements functionality to represent classical degrees of freedom (registers), which are updated using stochastic matrices. Any operation can be conditioned on the values of these registers, which allows the registers to affect the quantum state evolution. Analogously, the index of the sampled Kraus operators for a given sample trajectory can be used to apply conditional stochastic matrices to classical registers. This is relevant for simulations of leakage, as in our models we approximate all leakage transitions as incoherent. This is justified by the fact that, of the three error mechanisms causing leakage transitions, two (heating and dephasing-induced leakage) are incoherent processes, while coherent leakage arising from crosstalk is rapidly "dephased" as the neighboring measure qubits are measured and reset. The system state is therefore well approximated as block diagonal with respect to the leakage and computational subspaces. As a result of this approximation, no superposition is ever formed between the computational and leakage subspaces of any qubit, and we therefore are never required to keep more than two levels per qubit in our state vector. Instead we use classical registers to track the leakage status of each qubit, applying only the parts of our Kraus operators that act in the current computational subspace [86, 92, 93].

2. *Pauli+ simulator implementation*

Pauli+ carries out a classical simulation of the logical memory experiment. The term "classical" here denotes the fact that the simulation cost scales quadrat-

ically with the number of qubits and linearly with the number of error correction cycles, so it is amenable to classical computers. For a circuit with arbitrary Clifford gates and Pauli channel errors on qubits, the Gottesman-Knill theorem [94] provides a simulation scheme with polynomial scaling. Related methods have long been used for accurate threshold calculations [95–102], non-Pauli errors [103], and even for simulating circuits with a small fraction of non-Clifford gates [104, 105].

Pauli+ generalizes the standard *tableau* method [106] of stabilizer state tracking to also include qubit leakage. This results in a Markov-chain simulation based on an approximate Generalized Pauli Channel (GPC) error model similar to that used in Pauli-frame simulations by Fowler [107]. In our case, the state of the system is described by a vector of device leakage labels and an m-qubit stabilizer state for the devices in the computational (qubit) subspace. Note that throughout this section, we will refer to each multi-level transmon qubit as a "device." Additionally, we refer to the local energy eigenbasis of each transmon qubit as the "standard basis," with states $|0\rangle$ and $|1\rangle$ forming the span of the "computational subspace". In the context of multi-device operations, the computational subspace will refer to the tensor product of the relevant devices' computational subspaces.

The noisy circuit is modified for Pauli+ simulations by decomposing each noisy gate as a product of an ideal Clifford gate and a sequence of residual error channels. The error channels are then each approximated as a GPC. As a result, the entire simulation consists of four operation types:

1. A unitary that acts as a perfect Clifford gate in the computational subspace and identity on its complement.
2. A perfect measurement (all leakage levels resolved) in the standard basis.
3. A perfect single-device reset to state $|0\rangle$ in the standard basis.
4. An error channel, represented as a Generalized Pauli Channel (GPC).

(As noted previously, classical readout error is handled through post-processing of measurement data.) We describe how each of these operations are handled by the simulator below.

The full Hilbert space of the n-device system is approximated as a q^n-dimensional space, where each device has $q = 4$ energy levels represented by the local standard basis states $\{|i\rangle\}_{i=0}^{q-1}$. We assume that the states $|2\rangle$ and $|3\rangle$ are subject to rapid dephasing, so that the local Hilbert space of each device naturally decomposes as

$$\mathcal{H}_c \oplus \mathcal{H}_2 \oplus \mathcal{H}_3 \,,$$

where

$$\mathcal{H}_c = \mathrm{Span}\{|0\rangle\,, |1\rangle\}, \quad \mathcal{H}_2 = \mathrm{Span}\{|2\rangle\}, \quad \mathcal{H}_3 = \mathrm{Span}\{|3\rangle\}. \tag{8}$$

The full Hilbert space is separated into mutually incoherent subspaces given by Kronecker products

$$\mathcal{H}_{\bar{a}} = \mathcal{H}_{a[1]} \otimes \mathcal{H}_{a[2]} \otimes \ldots \otimes \mathcal{H}_{a[n]}, \tag{9}$$

where the jth vector entry $a[j] \in \{c, 2, 3\}$ denotes the local subspace of device j.

Each GPC acting on k devices in the index set $J = \{j_1, j_2, \ldots, j_k\}$ is represented by a collection of possible transitions between incoherent subspaces:

$$\bar{a}[J] \equiv (a[j_1], a[j_2], \ldots, a[j_k]) \rightarrow \bar{b}[J] \equiv (b[j_1], b[j_2], \ldots, b[j_k])\,. \tag{10}$$

For each transition $\bar{a} \rightarrow \bar{b}$, the set of devices in J are partitioned into four categories, depending on their initial and final leakage status. The devices which begin and *remain* in the computational subspace are denoted

$$R \equiv \{j \in J \mid a[j] = b[j] = c\}. \tag{11}$$

These devices will receive a standard Pauli channel. The devices that transition *up* to leakage are denoted by

$$U \equiv \{j \in J \mid a[j] = c, b[j] \neq c\}. \tag{12}$$

Leaked devices are not modified after the transition. Conversely, devices which transition *down* to the computational subspace are labeled

$$D \equiv \{j \in J \mid a[j] \neq c, b[j] = c\}. \tag{13}$$

We assume that such devices are fully disordered, meaning that they receive a maximally depolarizing Pauli channel, but we note that a more physically accurate distribution could be used here. To account for the fact that transitions occur in the standard basis, we may instead randomly prepare the device in one of the σ_z eigenstates. In the context of the twirling approximation (Sec. IV B 3), the probability of preparing one of the states could be set to match the device's $\langle\sigma_z\rangle$ observable, on the condition that the device decayed to the computational subspace. Finally, the remaining devices are those which start and end *leaked*,

$$L \equiv \{j \in J \mid a[j] \neq c, b[j] \neq c\}. \tag{14}$$

Like the devices in U, no Pauli channel is applied for these devices. However, transitions between different leakage subspaces are allowed.

Each GPC is decomposed into its actions with respect to the observed transitions. Each transition (denoted by the initial and final subspace vectors $\bar{\imath}$, $\bar{f}$ supported on J) is assigned a conditional probability $P(\bar{f} \mid \bar{\imath}) \equiv P(\bar{\imath} \rightarrow \bar{f})$. Whenever the GPC is applied to the system initially in subspace $\mathcal{H}_{\bar{a}}$, a unique subspace label $\bar{\imath} \equiv \bar{a}[J]$ is updated according to the distribution $P(\bar{f} \mid \bar{\imath})$. Additionally, each transition is associated with a standard Pauli channel acting on the qubits in R. The initial state of the qubits in U is traced over, while the final state for each qubit in D is a randomly selected $|0\rangle$ or $|1\rangle$ with equal probability

(equivalent to applying a maximally depolarizing channel). Overall, each GPC acting on devices in the index set J is represented by compound data

$$\left\{ \left(P(\bar{\imath} \to \bar{f}),\, p(\sigma_{\bar{\mu}}|\bar{\imath} \to \bar{f}) \right) \right\}_{\bar{\imath},\bar{f}}, \qquad (15)$$

where the Pauli operators $\bar{\sigma}_\mu$ act only on the qubits in the set R (Eq. (11)).

The Pauli+ simulator is implemented as a Markov chain tracking the incoherent subspace of each device and the current stabilizer state of the non-leaked devices. The full incoherent subspace is tracked using the label vector $\bar{a}$. At any point in the simulation, there may exist $m = m_{\bar{a}} \le n$ devices in the computational subspace. Their m-qubit stabilizer state is specified in terms of m Hermitian generators of the corresponding stabilizer group $\mathcal{S} = \langle \hat{G}_1, \ldots, \hat{G}_m \rangle$, an abelian subgroup of the m-qubit Pauli group $\mathcal{P}_m$ which does not include $-\hat{I}$. Notice that we track the stabilizer group but not the particular generators that represent the state. Thus at any point the set of generators can be changed by an arbitrary sequence of *row transformations* $\hat{G}_i \to \hat{G}_i\hat{G}_j$, $j \ne i$.

To collect a single sample, the simulation begins by initializing all n components of the leakage state label $\bar{a}$ to c and the $m = n$ qubits in the product state $|00\ldots 0\rangle$. Accordingly, the stabilizer generators are $\hat{G}_i = \sigma_z^{(i)}$, $i = 1, 2, \ldots, m$, where a single-qubit state $|0\rangle$ is a $+1$ eigenstate of σ_z. Subsequently, the simulator iterates over the operations in the circuit, at each step updating the stabilizer generators and possibly the leakage state label $\bar{a}$. The specific update rules are outlined below. (Note that $\hat{G}_i[j] \in \{I, \sigma_x, \sigma_y, \sigma_z\}$ refers to the component of $\hat{G}_i$ acting on device j.)

1. `clifford`: a Clifford unitary $\hat{U}$ acting on devices in the index set $J \subset \{1, 2, \ldots, n\}$.
   (i) If any of the devices in J are outside of the computational state ($a[j] \ne c$), do nothing.
   (ii) Otherwise, replace each stabilizer generator $\hat{G}_i$ with a Pauli operator $\hat{G}'_i = \hat{U}\hat{G}_i\hat{U}^\dagger$.

2. `measure`: an ideal standard basis measurement of device j.
   (i) If device j is leaked, record the leakage label $a[j]$ as the measurement outcome.
   (ii) Otherwise, use row transformations ($\hat{G}_i \to \hat{G}_i\hat{G}_j$) to select a set of generators so that (without loss of generality) either (a) $\hat{G}_1$ acting *only* on device j (i.e. it is unentangled), or (b) $\hat{G}_1$ and $\hat{G}_2$ the only generators acting on j (but also other devices), where $\hat{G}_1[j] = \sigma_x$ and $\hat{G}_2[j] = \sigma_z$.
   (iii) In case (a), if $\hat{G}_1[j] \in \{\sigma_x, \sigma_y\}$, or in case (b), select the result $\alpha \in \{0, 1\}$ randomly with equal probabilities and replace the first generator with $\hat{G}'_1 = (-1)^\alpha \sigma_z^{(j)}$. Record α as the measurement outcome.
   (iv) Otherwise, in case (a) with $\hat{G}_1[j] = \sigma_z$, use row transformations to do a full row reduction of the generator matrix so that $\hat{G}_1$ becomes a single qubit σ_z Pauli, $\hat{G}'_1 = (-1)^\alpha \sigma_z$. Record α as the measurement outcome.

3. `reset`: an ideal reset of device j to the state $|0\rangle$.
   (i) If the device j is leaked, set the label $a[j] = c$ and add an extra generator $\hat{G}_{m+1} = \sigma_z^{(j)}$ to the generating set. Increment $m \to m + 1$.
   (ii) Otherwise, do the partial row reduction in step 2(ii), and replace the first generator with $\hat{G}'_1 = +\sigma_z^{(j)}$.

4. `paulichannel`: Generalized Pauli Channel (GPC) acting on devices in the index set J.
   (i) Construct the initial subspace vector $\bar{\imath} = \bar{a}[J]$ and choose the final subspace vector $\bar{f}$ according to the probability distribution $P(\bar{\imath} \to \bar{f})$.
   (ii) For this outcome, select the set of devices U transitioning upward from the computational subspace. For each $j \in U$: (**a**) do partial row reduction as in step 2(ii); (**b**) drop the first generator $\hat{G}_1$ (swap it with $\hat{G}_m$ and decrement $m \to m - 1$); (**c**) if the qubit j was entangled with the rest of the system, replace $\hat{G}_2[j] = \sigma_z$ with the identity operator; (**d**) with probability $p = 1/2$, flip the sign of $\hat{G}_2$; (**e**) set leakage label $a[j]$ according to its value in $\bar{f}$.
   (iii) Select the Pauli error operator $\hat{E} = \bar{\sigma}_\mu$ according to the conditional distribution $p(\bar{\sigma}_\mu|\bar{\imath} \to \bar{f})$ supported on the set R of devices remaining in the computational subspace, and flip the signs of all the generators $\hat{G}_i$ which anticommute with the error $\hat{E}$: $\hat{G}'_i \equiv \hat{E}\hat{G}_i\hat{E}^\dagger = \pm\hat{G}_i$.
   (iv) For every device $j \in D$ coming down from leakage, (**a**) add a generator $\hat{G}_{m+1} = \pm\sigma_z^{(j)}$ with a random sign; (**b**) set $a[j] := c$; and (**c**) increment $m \to m + 1$.
   (v) For every device $j \in L$ that stayed leaked, update $a[j] = f[j]$ to account for possible transitions between $|2\rangle$ and $|3\rangle$.

We note that the generator processing steps for the `measure` and `reset` operations are equivalent to those implemented in ref [106]. Once the last operation in the circuit is reached, a record of all measurement results is kept for each sample.

3. Generalized twirling approximation

Given an arbitrary quantum channel $\mathcal{E}$ with Kraus operators K_j acting on devices J, the Generalized Pauli Twirling approximation allows us to define a corresponding GPC. This mapping corresponds to the following physical process:

1. For a given initial subspace $\mathcal{H}_{\bar{\imath}}$, double the number of degrees of freedom and prepare a maximally entangled state over the product $\mathcal{H}_{\bar{\imath}} \otimes \mathcal{H}_{\bar{\imath}}$, a direct product of EPR pairs. The devices whose index in $\bar{\imath}$ match the computational subspace and their matching ancillary pairs should be set as the $+1$ eigenstates of $\sigma_x \otimes \sigma_x$ and $\sigma_z \otimes \sigma_z$.

2. Apply $\mathcal{E}$ to the first subsystem.

3. Measure the leakage status of each device in the first subsystem, thereby determining the final subspace $\bar{f}$. This can be represented by the single-device projective measurement with elements
$$\begin{aligned} P_c &= |0\rangle\langle 0| + |1\rangle\langle 1| \\ P_2 &= |2\rangle\langle 2| \\ P_3 &= |3\rangle\langle 3| \end{aligned}$$

4. For the devices that remained in the computational subspace (R), measure the commuting stabilizers $\sigma_x \otimes \sigma_x$ and $\sigma_z \otimes \sigma_z$ acting on them and their entangled pair. Associate the resulting stabilizer eigenvalue pairs $(1,1)$, $(1,-1)$, $(-1,1)$, $(-1,-1)$ with the Pauli operators I, σ_x, σ_z, σ_y, respectively. The total error operator $\sigma_{\bar{\mu}}$ over all devices in R is the tensor product of all single qubit errors.

The conditional probability distribution $P(\bar{\imath} \to \bar{f}, \bar{\mu})$ is then defined as the probability of recording the outcomes $\bar{f}, \bar{\mu}$ assuming an initial state prepared in subspace $\mathcal{H}_{\bar{\imath}} \otimes \mathcal{H}_{\bar{\imath}}$. Accordingly, the extracted GPC will have the exact same statistics under the physical process represented above.

The probabilities $P(\bar{\imath} \to \bar{f}, \bar{\mu})$ can be determined directly from the Kraus operators K_j. For a given transition $\bar{\imath} \to \bar{f}$, Kraus operator K_j decomposed as a sum over tensor products for the devices in U, R, D, and L.

$$P_{\bar{f}} K_j P_{\bar{\imath}} = \sum_{\bar{u},\bar{d}} \left(K_j^{\bar{u},\bar{d},\bar{\imath}\to\bar{f}} \right)_R \otimes |\bar{f}[U]\rangle\langle\bar{u}|_U \otimes |\bar{d}\rangle\langle\bar{\imath}[D]|_D \otimes |\bar{f}[L]\rangle\langle\bar{\imath}[L]|_L$$

where $|\bar{u}\rangle, |\bar{d}\rangle$ sum over the tensor product of standard (normalized) basis vectors $(|0\rangle, |1\rangle)$ representing an orthonormal basis for the computational subspaces of devices U and D, respectively. The vectors $|\bar{f}[U]\rangle$, $|\bar{\imath}[D]\rangle$, and $(|\bar{\imath}[L]\rangle, |\bar{f}[L]\rangle)$ correspond to the leakage states of the devices which leaked, returned to the computational subspace, and remained leaked, respectively. (Note that the last operator in the tensor product can be used to represent transitions between leaked states.) The sub-block Kraus operator $K_j^{\bar{u},\bar{d},\bar{f}[U+L],\bar{\imath}[D+L]}$ can correspondingly be written as a partial tensor contraction,

$$K_j^{\bar{u},\bar{d},\bar{\imath}\to\bar{f}} = \left(\langle\bar{f}[U]|_U \otimes \langle\bar{d}|_D \otimes \langle\bar{f}[L]|_L \right) K_j \left(|\bar{u}\rangle_U \otimes |\bar{\imath}[D]\rangle_D \otimes |\bar{\imath}[L]\rangle_L \right).$$

Given the above decomposition, we can further break up the operators on the computational devices R by decomposing them as a linear combination of Pauli operators,

$$K_j^{\bar{u},\bar{d},\bar{\imath}\to\bar{f}} = \sum_{\bar{\mu}} c_{\bar{\mu}}^{j,\bar{u},\bar{d},\bar{\imath}\to\bar{f}} \sigma_{\bar{\mu}},$$

where the sum is taken over the Pauli operators $\sigma_{\bar{\mu}}$ acting on the devices in R; these operators are assumed to vanish on the leakage states $|2\rangle, |3\rangle$. (Below we will shorten the notations by dropping the extra fixed indices $\bar{\imath} \to \bar{f}$.) From this expansion, one may derive the conditional distribution, cf. Eq. (15):

$$P(\bar{\imath} \to \bar{f}, \bar{\mu}) \equiv P(\bar{\imath} \to \bar{f}) P(\sigma_{\bar{\mu}} | \bar{\imath} \to \bar{f}, \bar{\mu}) = \frac{1}{2^{|U|}} \sum_{\bar{u},\bar{d},j} \left| c_{\bar{\mu}}^{j,\bar{u},\bar{d}} \right|^2. \quad (16)$$

The prefactor accounts for the fact that each state $|\bar{u}\rangle$ exists in a computational subspace of dimension $2^{|U|}$. Finally, the sum itself is the average probability of applying $\sigma_{\bar{u}}$ on the qubits in R. In the case of no leakage transitions ($\bar{\imath} = \bar{f}$), Eq. 16 corresponds to the typical Pauli Twirling Approximation.

As a simple example, we apply the approximation to an error channel representing coherent rotation between states $|1\rangle$ and $|2\rangle$. The error channel is composed of a single unitary Kraus operator

$$\begin{aligned} U = \; & |0\rangle\langle 0| + \cos(\theta)\left(|1\rangle\langle 1| + |2\rangle\langle 2|\right) + |3\rangle\langle 3| \\ & + \sin(\theta)\left(|1\rangle\langle 2| - |2\rangle\langle 1|\right). \end{aligned}$$

Using the definitions of the single device projection operators, the non-vanishing blocks of the Kraus operator are

$$\begin{aligned} P_c U P_c &= |0\rangle\langle 0| + \cos(\theta)|1\rangle\langle 1| \\ &= \left(\cos^2(\theta/2)\sigma_I + \sin^2(\theta/2)\sigma_z\right) \cdot 1_U \cdot 1_D \cdot 1_L \\ P_c U P_2 &= \sin(\theta) \times (1_R \cdot 1_U \cdot |1\rangle\langle 2|_D \cdot 1_L) \\ & \quad +0 \times (1_R \cdot 1_U \cdot |0\rangle\langle 2|_D \cdot 1_L) \\ P_2 U P_c &= -\sin(\theta)\,(1_R \cdot |2\rangle\langle 1|_U \cdot 1_D \cdot 1_L) \\ & \quad +0 \times (1_R \cdot |2\rangle\langle 0|_U \cdot 1_D \cdot 1_L) \\ P_2 U P_2 &= \cos(\theta) 1_R \cdot 1_U \cdot 1_D \cdot |2\rangle\langle 2|_L \\ P_3 U P_3 &= 1_R \cdot 1_U \cdot 1_D \cdot |3\rangle\langle 3|_L \end{aligned}$$

where we have added trivial terms with zero coefficients to emphasize that the subspace $\mathcal{H}_c$ is two-dimensional. From this decomposition we may read off the non-vanishing Pauli coefficients:

$$\begin{aligned} c_I^{c\to c} &= \cos^2(\theta/2) \quad , \qquad c_Z^{c\to c} = \sin^2(\theta/2), \\ c_0^{u=1,c\to 2} &= \sin(\theta) \quad = -c_0^{d=1,2\to c}, \\ c_0^{2\to 2} &= \cos(\theta) \quad , \\ c_0^{3\to 3} &= 1. \end{aligned}$$

The conditional probabilities can now be read off using Eq. (16).

$\imath$	f	μ	$P(\imath \to f, \mu)$
c	c	I	$\cos^4(\theta/2)$
c	c	Z	$\sin^4(\theta/2)$
c	2	-	$\sin^2(\theta)/2$
2	c	-	$\sin^2(\theta)$
2	2	-	$\cos^2(\theta)$
3	3	-	1

Evidently, for each input state $\imath$, the sum of output probabilities equals one.

As expected, the probability to observe a transition down from state $|2\rangle$ is just the modulus square of the transition amplitude, $\sin^2(\theta) = |\langle 1| U |2\rangle|^2$. The reverse transition, though, has half this probability because a device in the computational subspace is (on average) in state $|1\rangle$ only half the time. A higher order effect caused by this twirling is the dephasing observed in the $c \to c$ transition. This can be interpreted as a measurement "back-action": because the device was not observed to leak and only state $|1\rangle$ can leak, the channel acts like a weak measurement in the standard basis.

C. Comparison of Pauli+ and quantum simulations

We tested the validity of the Generalized Twirling Approximation used by the Pauli+ model by doing a direct comparison with the equivalent *kraus_sim* quantum trajectories simulation. The comparison considered the distance-3 Z-basis and X-basis experiments associated with the West device configuration. Each 25-cycle simulation corresponded to 200,000 samples. The classical Pauli+ simulation required about 160 seconds (distributed over 16 CPU cores), while the quantum simulation required 11 hours. Note that the *qsim* backend for the *kraus_sim* simulator has not been optimized for performance on these system sizes. Decoding was implemented using the algorithm in Ref. [70], with detection cluster probabilities inferred from the p_{ij} matrix.

As can be seen in Figs. S17-S19, there is generally quite good agreement between both simulations with respect to logical error and typical detection event statistics. One reason this may be the case is that the surface code measurement circuit implies a stabilizer-state projection at the end of each cycle. This has the effect of "twirling" the quantum noise, so that for a single data qubit error in the absence of leakage the approximation is exact [108]. The same is true for errors in non-neighboring circuit locations. With the net error rates $\sim 1\%$ per gate and a total of $n \sim 20$ qubits, we expect around one error per cycle. Moreover, the added depolar-

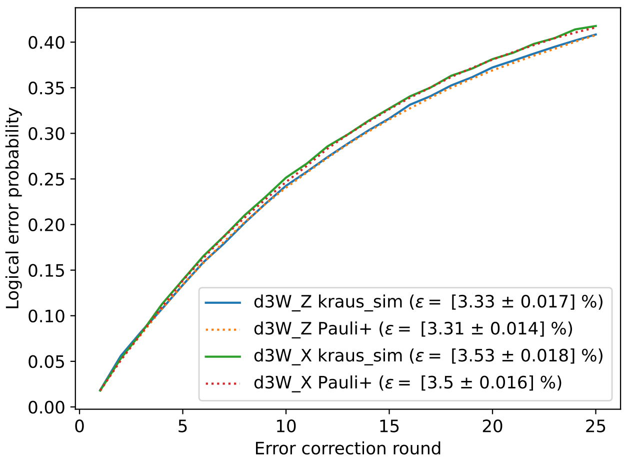

FIG. S17. Comparison of simulated logical error probabilities for the distance-3 West, X and Z basis experiments. Each data point is the logical error fraction over $N = 2 \times 10^5$ samples, which estimates the logical error probability p. This is modeled as a Bernoulli process with standard deviation $\sqrt{p(1-p)}$, so the standard deviation of the sample average is $\sqrt{p(1-p)/N} \leq 1/\sqrt{4N} \approx 0.11\%$. The cited uncertainties for the logical error per cycle are derived by propagating these Bernoulli standard deviations through the weighted linear fit described in Sec. III.

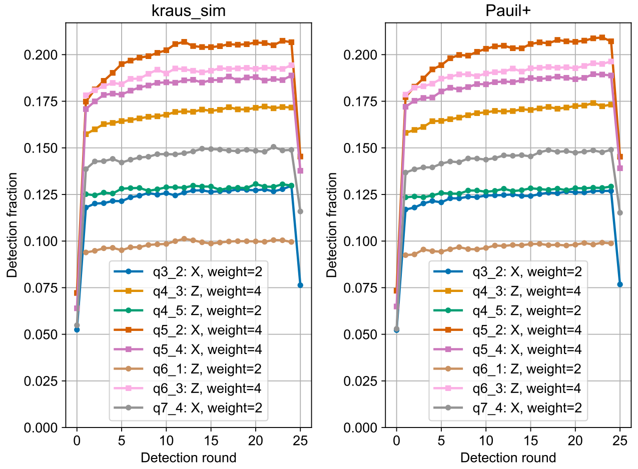

FIG. S18. Comparison of simulated detection probabilities for the distance-3 West, X-basis experiment. As in the experimental data, we see a slow increase in detection probability as the number of cycles progresses. This can be attributed to leakage accumulation in the data qubits.

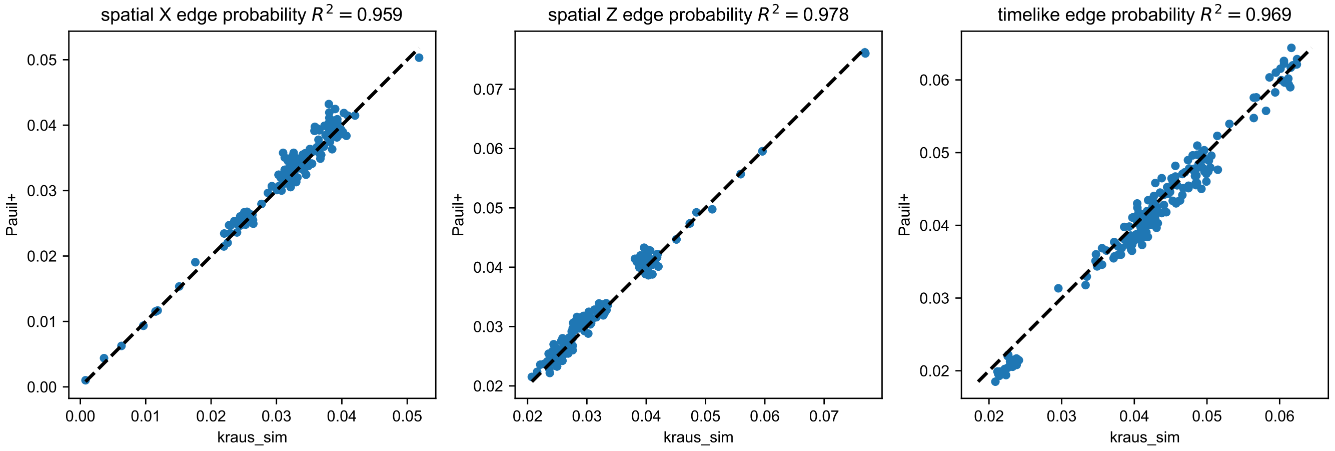

FIG. S19. Comparison of simulated spacelike and timelike edge probabilities for the distance-3 West, X-basis experiment. From left to right, the scatter plots correspond to "X" spatial edges (between stabilizers in the initial state basis), "Z" spatial edges (between stabilizers with undetermined initial value), and timelike edges (between the stabilizers separated by one error correction cycle). The R^2 quantities reported above correspond to the coefficient of determination between the "observed" (kraus_sim) and "predicted" (Pauli+) edge probabilities.

izing errors after each gate operation and idle are exactly described by a Pauli channel.

The level of agreement between the two simulations is contrasted by some results in the literature. We expect the two simulations to disagree in the presence of considerable coherent effects: our simulations include some unitary errors due to crosstalk, which may add coherently in a quantum trajectories simulation [109], but this may be suppressed due to non-local nature of these errors. Additionally, early work [110] simulating smaller distance codes suggested that the Twirling Approximation under both decoherence and unitary gate errors strongly over-predicted the expected logical error (by factors of 2 or more). Yet early numerical test of the distance-3 surface code [80] found good agreement with the Pauli Twirling Approximation under amplitude damping (T_1 decay) and (white noise) dephasing, but only in the logical Z basis (twirling over-predicted the error for the logical X). There are several factors that may have contributed to reducing these effects in our simulations. First, the Hadamard operations in our experiments are chosen so that the stabilizer generators describing our system are all of mixed $ZXXZ$ type [46, 47]. This has the effect of approximately symmetrizing all bit-flip and phase-flip errors. Second, we deliberately insert X gates whenever a qubit is idle, which is known to reduce coherent errors. Overall, the good agreement between the exact quantum trajectories and a classical simulations indicates that the net contribution of coherently-added errors is not large enough to see.

V. SENSITIVITY OF LOGICAL ERROR PER CYCLE TO PHYSICAL ERRORS

In this section we discuss the sensitivity coefficients of the surface code logical error per cycle (ε) to errors in the component operations of the surface code circuits - namely, controlled-Z (CZ) gates, single qubit (SQ) gates, data qubit idle (including the dynamical decoupling operations during readout and reset), and readout and reset. Additionally, we also estimate the sensitivity coefficients of ε to leakage and crosstalk.

To estimate the sensitivity coefficients, we simulate the operation of a surface code logical qubit with an error model corresponding to our experimental implementation, or the *experimental operation point*. For the purposes of this analysis, we use the Pauli+ simulation as described previously, along with correlated minimum-weight perfect matching for efficiency. Then, for a certain

Component	Error probability
SQ gates	1.09×10^{-3}
CZ gates	6.05×10^{-3}
Data qubit idle	2.46×10^{-2}
Reset	1.86×10^{-3}
Readout	1.96×10^{-2}
CZ leakage	2.0×10^{-4}
Leakage from heating	6.6×10^{-4}
CZ crosstalk	9.5×10^{-4}

TABLE I. Average probabilities of main errors at the operation point of distance-5 surface code experiments. We point out that the last three rows do not correspond to actual components of the surface code circuit, but we include them for future reference.

type of component error (e.g., SQ gate error), we change all error rates of the same type by varying amounts, while resimulating the logical error per cycle for each change. Given that the changes to the component errors are small, we assume a linear relationship between logical error per cycle and component error rates, such that the sensitivity coefficient ν_{error} can be defined as

$$\nu_{\text{error}} = \frac{\delta\varepsilon}{\delta p_{\text{error}}}, \tag{17}$$

where $\delta\varepsilon$ is the change of the logical error per cycle due to a change δp_{error} of the error probability of all components of the same type. Thus, the sensitivity coefficient can be found by linear regression of the simulated logical error per cycle against changes in component error rate.

A. Experimental operation point

The experimental operation point of the distance-5 surface code experiment is specified by the error probabilities of all components in the surface code circuit as well as by parameters that characterize the strength of leakage and crosstalk. We independently measure the component error probabilities to be used in the simulator as follows.

- SQ gate errors are measured by simultaneous randomized benchmarking (RB) on all qubits
- *CZ* gate errors are measured using four simultaneous cross-entropy benchmarking experiments (XEB), each characterizing one of the four sets of *CZ*s that are executed simultaneously during the surface code circuit
- Data qubit idle errors are measured by simultaneous interleaved RB on the data qubits, where the interleaved operation consists of measurement and reset of the measure qubits and dynamical decoupling on the data qubits
- Readout errors are measured by preparing random classical states on the measure qubits and performing a correlated measurement on all of the measure qubits. The readout error for each qubit is found by computing the probability that the qubit was measured in the expected state, marginalized from the correlated measurement data.
- Finally, the reset error is estimated by preparing $|0\rangle$ or $|1\rangle$ on all measure qubits, performing measurement and reset on all the measure qubits, then measuring the measure qubits again. The reset error for each qubit is the residual $|1\rangle$ population in the second measurement averaged over the initial states. Note that for the purpose of sensitivity analysis, we combine reset and readout errors.

The mean Pauli error probability for gates, and mean error probability for readout and reset, aggregated over all components of the same type, are given in the first five rows of Table I. We note that the simulator uses individual error probabilities for these components rather than the mean.

We incorporate leakage into the simulator from two different sources. First, we assume that leakage is generated on the high frequency qubit of a *CZ* gate (e.g., due to qubit dephasing during *CZ* operation) with a nominal probability of

$$p_2^{\text{CZ}} = 0.25\, p_t = 2.0 \times 10^{-4}, \tag{18}$$

where we assume $p_t = 8 \times 10^{-4}$ for all *CZ*s (see Sec. IV A 3) and the factor 0.25 is the probability that both qubits are in state $|11\rangle$ during a *CZ* gate. Second, we assume leakage can be generated from a non-equilibrium "heating" process on each qubit, characterized by the parameter $\Gamma_{1\to2}$. For our analysis, we assume that each qubit has a heating rate $\Gamma_{1\to2} = 1/(700\,\mu\text{s})$, which is the mean value from experimental measurements. While $\Gamma_{1\to2}$ is the varied parameter for sensitivity analysis, in Table I we report a typical leakage error due to heating per QEC cycle as

$$p_2^{\text{heating}} = 0.5\,\Gamma_{1\to2}\, t_{\text{cycle}} \approx 6.6 \times 10^{-4}, \tag{19}$$

where the factor 0.5 is the probability that the qubit is in state $|1\rangle$ during one QEC cycle.

Finally, we also estimate the sensitivity of logical error per cycle to stray interactions during simultaneous operation of the *CZ* gates. These unwanted interactions originate from the tunable coupler design and heavily depend on the frequency configuration of both qubits and couplers [111]. Unlike the control crosstalk discussed in Sec. I F, crosstalk due to stray interactions cannot be mitigated with active cancellation since the strengths of the stray interactions are dependent on the state of the qubits. While stray interactions can in principle occur during single qubit gate and data-qubit idling, we focus only on the *CZ* gate crosstalk, where interaction crosstalk is the most pronounced since both the qubit and coupler frequencies are modulated. To estimate the crosstalk operating point, for each *CZ* we compute the error due to interactions with each of the states in the qubits neighboring the *CZ* pair, some of which may also be executing *CZ* gates. Then, we average over all *CZ* pairs and crosstalk error sources, yielding a mean additional Pauli error probability of

$$p_{\text{CZ}}^{\text{crosstalk}} = 9.5 \times 10^{-4}, \tag{20}$$

which is roughly 15% of the measured total *CZ* error. The computed crosstalk error is largely consistent with the observed difference between isolated and parallel *CZ* errors. Possible limitations in our calculations of crosstalk include imprecise estimations of the coupler frequencies, since we cannot directly probe the coupler.

Component	sensitivity (d3)	sensitivity (d5)
SQ gates	6.2 ± 0.3	8.4 ± 0.2
CZ gates	2.13 ± 0.08	4.0 ± 0.2
Data qubit idle	0.91 ± 0.02	1.05 ± 0.03
Readout or reset	0.26 ± 0.01	0.41 ± 0.02
Leakage (heating)	2.3 ± 0.3	3.9 ± 0.8
CZ crosstalk	5.0 ± 0.5	6.5 ± 0.6

TABLE II. Logical error per cycle sensitivities at the experimental operation point.

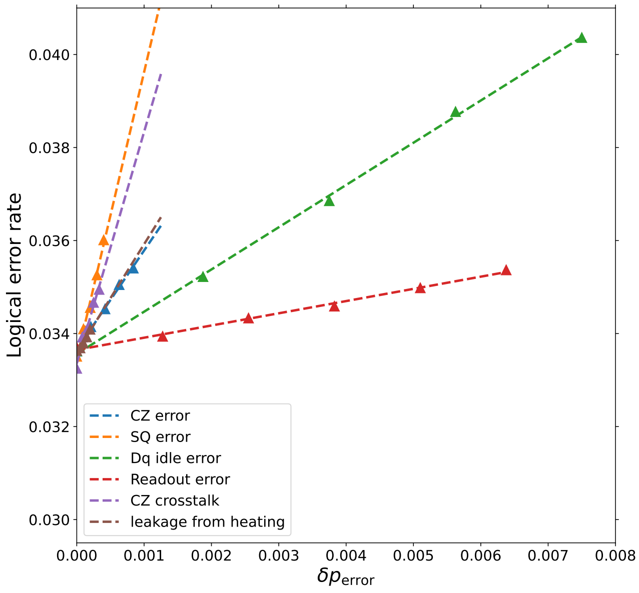

FIG. S20. Simulation of logical error per cycle for a distance-3 surface code while varying component errors of various types. The depicted logical error per cycle values are averaged over the four distance-3 surface code grids. The variable δp_{error} is the additive change of the corresponding error probability for each qubit (SQ error, data qubit idle error, readout error, leakage from heating) or pair of qubits (CZ error and CZ crosstalk). Dashed lines are linear fits to the simulation data (markers) and their slopes are the sensitivities reported in Table II.

B. Logical error per cycle sensitivities for distance-3/5 surface codes

Figure S20 shows the result of simulating logical error per cycle while sweeping each component error rate for the distance-3 code. Figure S21 likewise shows the simulation results for the distance-5 code. Table II shows the sensitivity coefficients for the distance-3 and distance-5 logical error per cycle as obtained by linear regression of the simulation results. We observe that the distance-5 surface code is more sensitive to errors than the distance-3 surface code, consistent with the experimental observation in Fig. 3 that distance-5 logical performance improved more quickly than distance-3 when component errors were improved. The results also indicate that logical error per cycle is most sensitive to gate error, CZ crosstalk, and leakage.

C. Logical error per cycle sensitivity vs $1/\Lambda_{3/5}$ sensitivity

To build the error budget presented in Fig. 4a, we additionally analyzed the component error sensitivity of the inverse of the error suppression factor, $1/\Lambda_{3/5}$. This analysis proceeds similarly to the logical error per cycle sensitivity analysis, with two primary differences. First, we assume that all component errors of the same type have the same error rate. Second, following the methodology of Ref. [44], the sensitivities are evaluated at the half-operation point - in other words, all component errors of the same type will have an error probability that is 0.5 times the error probability shown in Table I). As explained in Ref. [44], the choice of half-operation point is based on the assumption that $1/\Lambda_{3/5}$ is a second-order function of component errors. The contributions to $1/\Lambda_{3/5}$ for each error type are then computed by multiplying the simulated sensitivity and the mean error rate at the experimental operation point (not the half-operation point).

VI. DIAGNOSTICS USING p_{ij} OF SURFACE CODES

We analyze the pairwise detection event correlations in the surface code data using the p_{ij} *correlation matrix method* that was introduced in [44, 51, 52]. This method quantifies the probability p_{ij} of simultaneously triggering two detection events at the error detection nodes i and j. The p_{ij} probability of an edge between nodes i and j is approximately equal to the sum of the probabilities of all error processes that produce clusters of detection events including the nodes i and j. In Sec. VI C we discuss how to estimate the probability of clusters of arbitrary size, while in this section we focus on edges, which are used for surface code diagnostics. We note that the extension to larger clusters helps filter the probability of p_{ij} down to only those error processes that trigger nodes i and j exactly, which is used for decoding. The p_{ij} probability of an edge between the nodes i and j is given by

$$p_{ij} = \frac{1}{2} - \frac{1}{2}\sqrt{1 - \frac{4\left(\langle x_i x_j\rangle - \langle x_i\rangle\langle x_j\rangle\right)}{1 - 2\langle x_i\rangle - 2\langle x_j\rangle + 4\langle x_i x_j\rangle}}, \qquad (21)$$

where $x_i = 0$ if there is no detection event at the node i; otherwise, $x_i = 1$. The derivation of formula (21) is given in Ref. [44]. The averages $\langle x_i\rangle$, $\langle x_j\rangle$ and $\langle x_i x_j\rangle$ are evaluated from detection event data, which in our surface code experiments consist of 50,000 experimental realizations. In our experiments $p_{ij} \ll 1$ so that (21) can

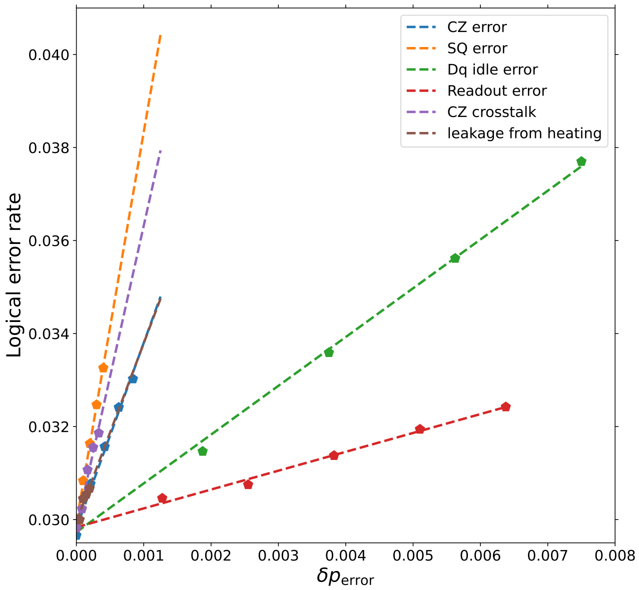

FIG. S21. Simulation of logical error per cycle for a distance-5 surface code while varying component errors of various types.. Similarly to Fig. S20, dashed lines are linear fits to the simulation data (markers) and their slopes are the sensitivities reported in Table II.

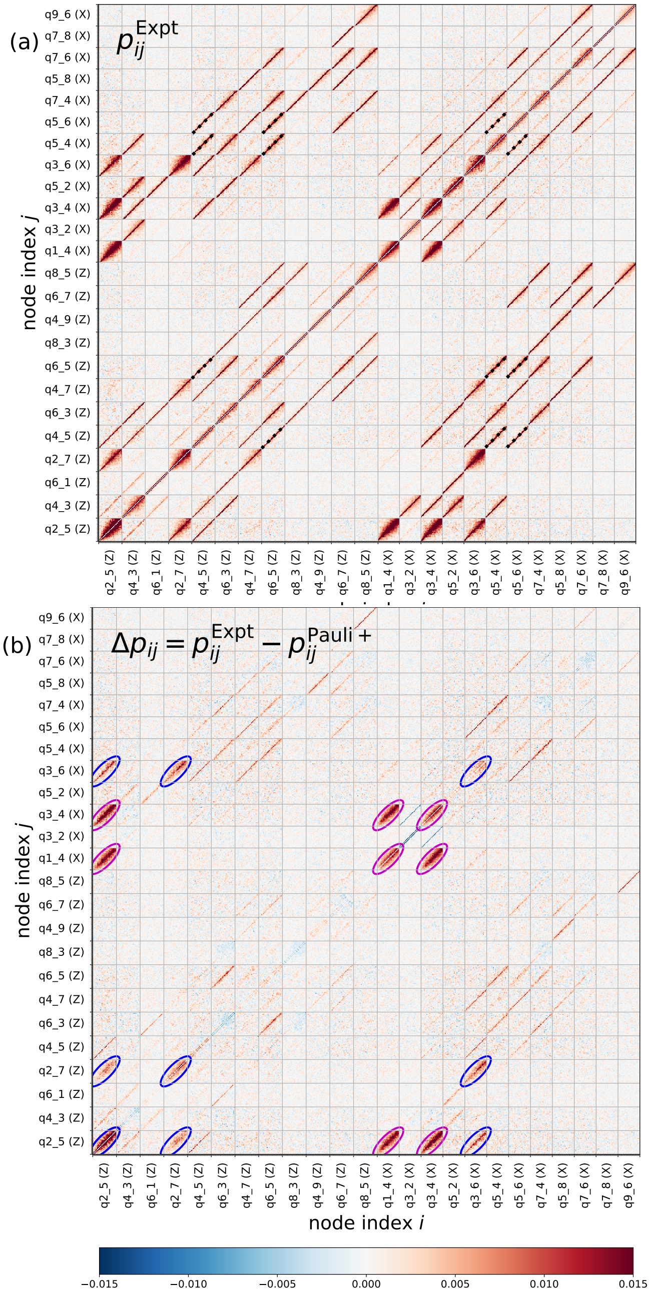

FIG. S22. Correlation matrix p_{ij}. Panel (a) illustrates the 600×600 symmetric correlation matrix p_{ij} for a 25-cycle distance-5 Z-basis surface code experiment. Each detection node $i = (t, s)$ has time coordinate t indicated by the minor tick and a spatial coordinate s indicated by the measure qubit labels (s = q2_5, q4_3, ... q9_6). Panel (b) illustrates the difference of the p_{ij} correlation matrices between the experimental and simulation Pauli+ data. The ellipses indicate the main discrepancies between the experiment and simulation, which are likely due to excess leakage accumulation in a pair of data qubits (2_6 and 2_4) during the surface code experiment. Dotted lines in panel (a) indicate the matrix elements that are affected by idle Y errors on the middle data qubit 5_5.

be approximated as

$$p_{ij} \approx \frac{\langle x_i x_j \rangle - \langle x_i \rangle \langle x_j \rangle}{(1 - 2\langle x_i \rangle)(1 - 2\langle x_j \rangle)} \,. \tag{22}$$

One can then think of p_{ij} as the usual covariance, normalized by $(1 - 2\langle x_i \rangle)(1 - 2\langle x_j \rangle)$.

The probability $p_{i\,\mathrm{B}}$ of a boundary edge at the node i is estimated by the formula

$$p_{i\,\mathrm{B}} = \frac{\langle x_i \rangle - p_{i,\Sigma}}{1 - 2p_{i,\Sigma}} \,, \tag{23}$$

where $p_{i,\Sigma}$ is the sum of p_{ij} probabilities of all edges connected to the node i,

$$p_{i,\Sigma} = g(p_{ij_k}, \ldots g(p_{ij_3}, g(p_{ij_2}, p_{ij_1}))\ldots), \tag{24}$$
$$g(p, q) \equiv p(1 - q) + (1 - p)q = p + q - 2pq, \tag{25}$$

with $g(p, q)$ being the effective sum of probabilities p and q. Note that $g(p, q) \approx p + q$ if the probabilities p and q are small. In practice, we use the standard circuit-level error model to determine the boundary edges and the bulk edges that connect to the boundary nodes. We then use Eq. (21) and Eqs. (23)–(25) to evaluate the boundary edge probabilities.

The p_{ij} method provides the correlation probability for any pair of nodes i and j, but we will focus here on the dominant pairwise correlations that we observe in the data: timelike, spacelike, and spacetimelike (diagonal)

edges. We also compare the experiment and Pauli+ simulation p_{ij} correlation matrices. The experimental data that is used to compare against simulations is from the 25 cycle, distance-5 surface code experiment with data qubits prepared in the Z basis.

A. Experiment *vs.* Pauli+ p_{ij} correlation matrices

Panel (a) of Fig. S22 is a graphical representation of the 600×600 symmetric correlation matrix p_{ij} for the considered distance-5, 25 cycle, Z-basis experiment. The number of detection nodes i is $12 \times 26 + 12 \times 24 = 600$, since for the twelve measure qubits that measure the Z stabilisers, we have 26 detectors (an additional layer comes from the final data qubit measurements), while for the remaining twelve measure qubits that measure the X stabilisers, we have 24 detectors (since the values of the X-stabiliser measurements are initially random). The detectors $i = (t, s)$ have a time, space coordinate t, s. In Fig. S22(a), the minor ticks indicate time coordinates while the major ticks indicate space coordinates, e.g. $s =$ q2_5(Z) for the measure qubit 2_5 that measures a Z-stabiliser. Note that although we use the $ZXXZ$ variant of the surface code, all notations for stabilizers here assume the traditional surface code labels.

There are two distinct types of features in the p_{ij} correlation matrix illustrated in Fig. S22(a). Narrow reddish features (e.g. the one inside the qubit-qubit block q8_5–q6_5) can be explained by conventional Pauli errors in the surface code circuit. Such conventional errors manifest in the p_{ij} matrix as one-pixel-wide reddish features since these errors produce detection events separated in time by 0 or 1 cycles. This includes Pauli errors that produce many-body clusters of detection events (e.g., idle Y-type data qubit errors). For instance, idle Y-errors on the middle data qubit 5_5 increase the p_{ij} matrix elements indicated by the black dotted lines in the upper panel of Fig. S22. In addition, we also see some broader reddish features (e.g. qubit-qubit block q1_4–q2_5) that indicate the presence of long-lived time correlations between detection events. Such correlations are likely caused by leakage accumulation in the data qubits.

Panel (b) of Fig. S22 shows the difference between the p_{ij} correlation matrices from experimental data and Pauli+ simulation data used in the main text. We see that the p_{ij} matrix difference looks much cleaner (with fainter reddish regions than the experimental p_{ij} matrix shown in Fig. S22(a)). This indicates that Pauli+ simulation does a good job in capturing most of the detection event correlations that are present in the experiment. There are, however, some spatial/temporal correlations that are significantly stronger in the experiment than in the simulation, indicated by the ellipses in the p_{ij} matrix difference. We attribute these features to stronger-than-usual leakage in two data qubits: 2_4 and 2_6. Leakage accumulation in these data qubits leads to detection events that may be correlated locally

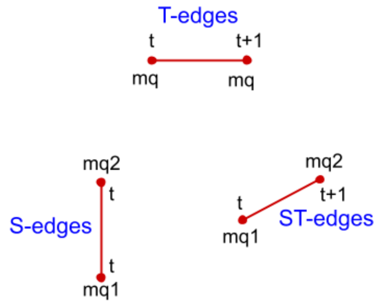

FIG. S23. Main pairwise detection event correlations in the surface code. The red dots indicate detection events. The considered pairwise correlations (edges) are timelike (T) edges, spacelike (S) and spacetimelike (ST) edges. These correlations are mainly caused by readout/reset errors, idle data qubit errors, and CZ errors, respectively.

in space (with detection events at neighboring measure qubits) but nonlocally in time (with a correlation time of roughly $T_1/2 \approx 10\,\mu$s). These correlations produce the reddish regions indicated by the magenta and blue ellipses for leakage accumulation in data qubits 2_4 and 2_6, respectively. We stress that the Pauli+ simulation can capture these missing correlations by properly adjusting the simulation parameters related to leakage generation. Besides the broad reddish regions indicated by the ellipses in Fig. S22(b), we also notice some faint narrow reddish features in the p_{ij} matrix difference. These features indicate short temporal correlations of detection events separated in time by 0 or 1 cycles - they can be understood as a discrepancy between the actual component error probabilities during the surface code experiment and the component error probabilities used in the simulation. To quantify this discrepancy, we need to compare the p_{ij} probabilities for the edges of the surface code error graph.

B. Probabilities in p_{ij} of main error edges in the surface code

In this section we examine the probabilities of the main pairwise correlations (error edges) in the distance-5 surface code. These error edges are depicted in Fig. S23. Timelike edges (T-edges) indicate correlations between detection events occurring at the same measure qubit and separated in time by 1 cycle. In our experiments, T-edges are mainly caused by readout/reset errors, and to a lesser extent, by SQ- and CZ-errors. In addition, leakage and crosstalk can also contribute to the T-edge probabilities. Note that since leakage states in the data qubits survive for several rounds (with an approximate lifetime of $10\,\mu$s), leakage in the data qubits not only leads to T-edges but also to correlations between nodes separated in time by more than 1 cycle. Spacelike edges (S-edges) indicate correlations between detection events

occurring in the same cycle at two measure qubits of the same type (X or Z). We consider two types of spacelike edges, namely SX (spacelike edges between X measure qubits) and SZ edges (spacelike edges between Z measure qubits). In the $ZXXZ$ surface code implementation, SZ edges mainly come from idle T_1 errors in roughly half of the data qubits and idle T_2 errors in the remaining data qubits (see e.g. the labels T1 and T2 in Fig. S26). SQ- and CZ-errors as well as leakage and crosstalk also contribute to spacelike edges. Finally, spacetimelike edges indicate correlations between detection events separated in time by 1 cycle and occurring on different measure qubits. Spacetimelike edges that come from Pauli CZ errors are denoted as ST edges, while other spacetimelike edges that are not expected from usual CZ errors are denoted as ST' edges. The latter are likely due to leakage or crosstalk errors.

1. *Timelike edges*

The probability of T-edges are obtained from the p_{ij} correlation matrix with $i = (t, s)$ and $j = (t+1, s)$, where the space coordinate s refers to one of the measure qubits. It is convenient to average over time t and present the time-averaged T-edge probabilities as shown at the top panel heatmap of Fig. S24. This way of presenting the p_{ij} data for the T-edges is particularly useful to spot measure qubits with good or bad readout/reset errors. We reemphasize that, although readout/reset errors are the main contributors to the T-edges in our experiment, other physical errors can also contribute to the T-edge probabilities.

The average T-edge probability averaging over all qubits and cycles are

$$p_{ij}^{\text{T-edge, mean}} = 3.85 \times 10^{-2} \quad (26)$$
$$[\text{Pauli+ sim} = 3.7 \times 10^{-2},$$
$$\text{Pauli sim} = 3.0 \times 10^{-2}].$$

The lower panel heatmap of Fig. S24 shows the difference between the time-averaged T-edge probabilities in experiment and Pauli+ simulation. We see that the difference is relatively small, indicating a good agreement between experiment and simulation. The biggest discrepancy between experiment and simulation is for T-edges on measure qubits 1_4, 3_4, 2_5, 2_9, which are spatially close to the leaky data qubits 2_4 and 2_6. Thus, the reason for this discrepancy is most likely leakage accumulation in these data qubits, as discussed in Sec. VI A.

The upper heatmap in Fig. S24 illustrates the usefulness of the p_{ij} method as an error-diagnostic tool: it indicates which qubits or gates are underperforming.

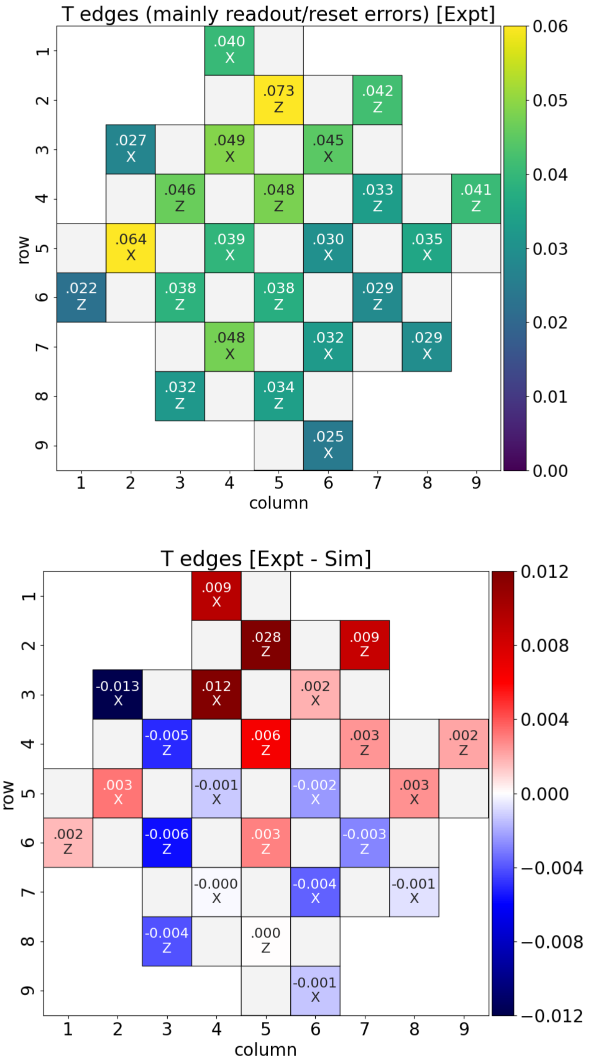

FIG. S24. Top panel: timelike edge probabilities. Each value in the heatmaps represents the median of the T-edge probabilities of a measure qubit. Measure qubits that measure X- or Z-stabilisers are indicated by X or Z. The Top panel shows the time-averaged T-edge probabilities for the 25 cycles distance-5 surface code experiment with data qubits initialized in the Z-basis. Bottom panel shows the difference of T-edge probabilities between experiment and Pauli+ simulation.

2. *Spacelike edges*

The heatmap at the top panel of Fig. S25 shows the time-averaged probabilities of SX edges for the distance-5 surface code experiment in the Z-basis. To explain the information displayed in this heatmap, let us consider the middle data qubit 5_5. A phase-flip error in this data qubit during idle time produces two detection events at the neighboring X measure qubits 5_4 and 5_6 (see horizontal red edge in Fig. S25) at some cycle t. The number 0.043 labeling the middle tile $(5, 5)$ is the median (over

cycles) of the p_{ij} probabilities of all SX edges between these two X measure qubits. Idle Y errors on data qubit 5_5 also contribute to the displayed probability 0.043; for this reason, we write 'T2' below 0.043 to remind us that this SX edge probability may come from Z or Y idle errors (and likely more error mechanisms). Let us consider another example: an idle X error (due to T_1 qubit decay) in data qubit 6_4 produces an SX edge between the X measure qubits 5_4 and 7_4 (vertical red edge in Fig. S25).
Idle errors along the boundary data qubits of the surface code grid sometimes produce a single detection event (i.e. a boundary edge). For instance, an idle Z error on data qubit 1_5 produces only one detection event at X measure qubit 1_4. We evaluate the probabilities of the boundary edges at the X measure qubit 1_4 using Eq. (23) for all times t, and then the time-averaged value (0.029) is indicated in tile $(1,5)$ of the upper heatmap of Fig. S25. Finally, we can also have idle errors in two different boundary data qubits produce the same detection event or boundary edge. For instance, an X error in data qubit 6_2 and a Z error in data qubit 5_1 both produce the same boundary edge at the X measure qubit 5_2. In this ambiguous situation, we divide the probability of the boundary edge (given by Eq. (23)) at the X measure qubit 5_2 among these data qubits (in this example 0.03 in the tiles $(5,1)$ and $(6,2)$), with asterisks denoting those data qubit tiles.

Figure S26 is complementary to Fig. S25 - it displays the time-averaged SZ edge probabilities. For instance, for the middle data qubit 5_5, we read an SX edge probability of 0.043 from Fig. S25, and an SZ edge probability of 0.031 from Fig. S26. The bottom panels of Figs. S25 and S26 show the difference between the experimental and simulated values, which is relatively small.

The average spacelike edge probabilities are

$$p_{ij}^{\mathrm{SX-edge,\,mean}} = 3.4\times 10^{-2} \quad (27)$$
$$[\text{Pauli+ sim} = 3.3\times 10^{-2},$$
$$\text{Pauli sim} = 3.1\times 10^{-2}],$$

and,

$$p_{ij}^{\mathrm{SZ-edge,\,mean}} = 3.2\times 10^{-2} \quad (28)$$
$$[\text{Pauli+ sim} = 3.0\times 10^{-2}$$
$$\text{Pauli sim} = 2.9\times 10^{-2}].$$

3. *Spacetimelike edges*

Within the standard circuit error model, spacetimelike edges are due to CZ errors. Those spacetimelike edges are referred to as ST-edges. In the experiment and in the Pauli+ simulations, we also find other unexpected spacetimelike edges that we denote as ST' edges, which are mainly due to leakage accumulation in the data qubits.

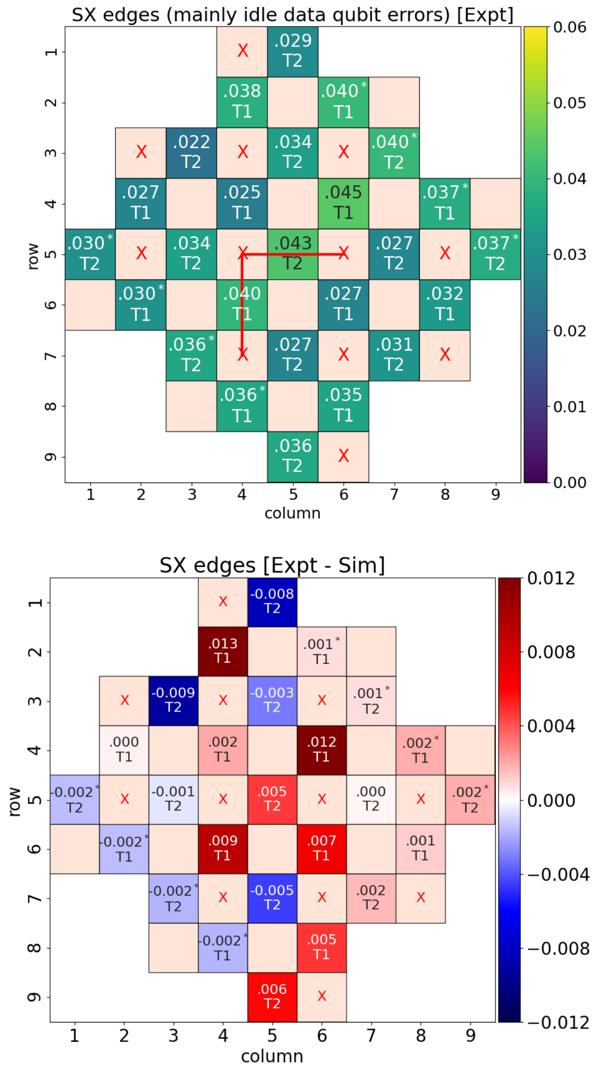

FIG. S25. Top panel: time-averaged p_{ij} probabilities of SX edges. Pink tiles with an X indicate X-measure qubits, empty pink tiles indicate Z-measure qubits. The red horizontal edge indicates an SX edge between the X measure qubits 5_4 and 5_6. Their probabilities are obtained from the p_{ij} matrix with $i=(s,t)$ and $j=(s',t)$, and $s=$ q5_4 and $s'=$ q5_6. After averaging over time t, we write the result in the middle data qubit tile (5,5) since phase-flip errors in the data qubit 5_5 would produce such SX edges. Asterisks indicate ambiguous data qubits (e.g., the pair 5_1 and 6_2) with idle errors that produce the same boundary edge (see Sec. VI B 2). Lower panel: the difference between the time-averaged SX edge probabilities in the experiment and the Pauli+ simulation. Labels T1 and T2 at the tiles indicate the type of idle data qubit error that give rise to the SX edges (see Sec. VI B 2).

We expect that leakage in the data qubits should increase the ST- and ST'-edges by roughly the same amount, and this is indeed validated by the experimental and simulation data, as shown below.

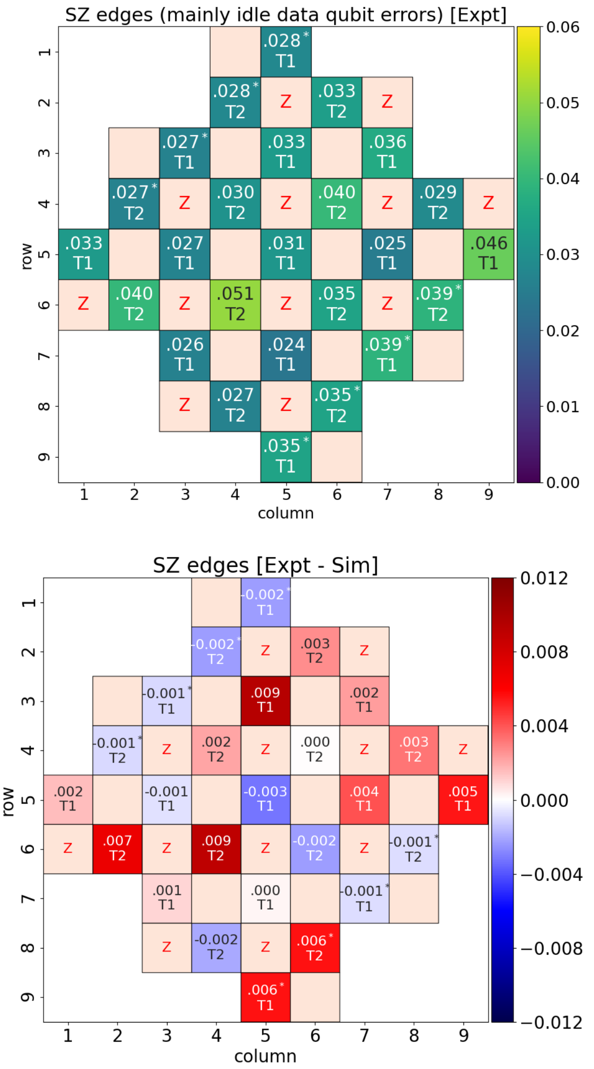

FIG. S26. Top panel: time-averaged p_{ij} probabilities of SZ edges. Description of the information displayed is similar to that of Fig. S25. Lower panel: the difference between the time-averaged SZ edge probabilities in the experiment and the Pauli+ simulation.

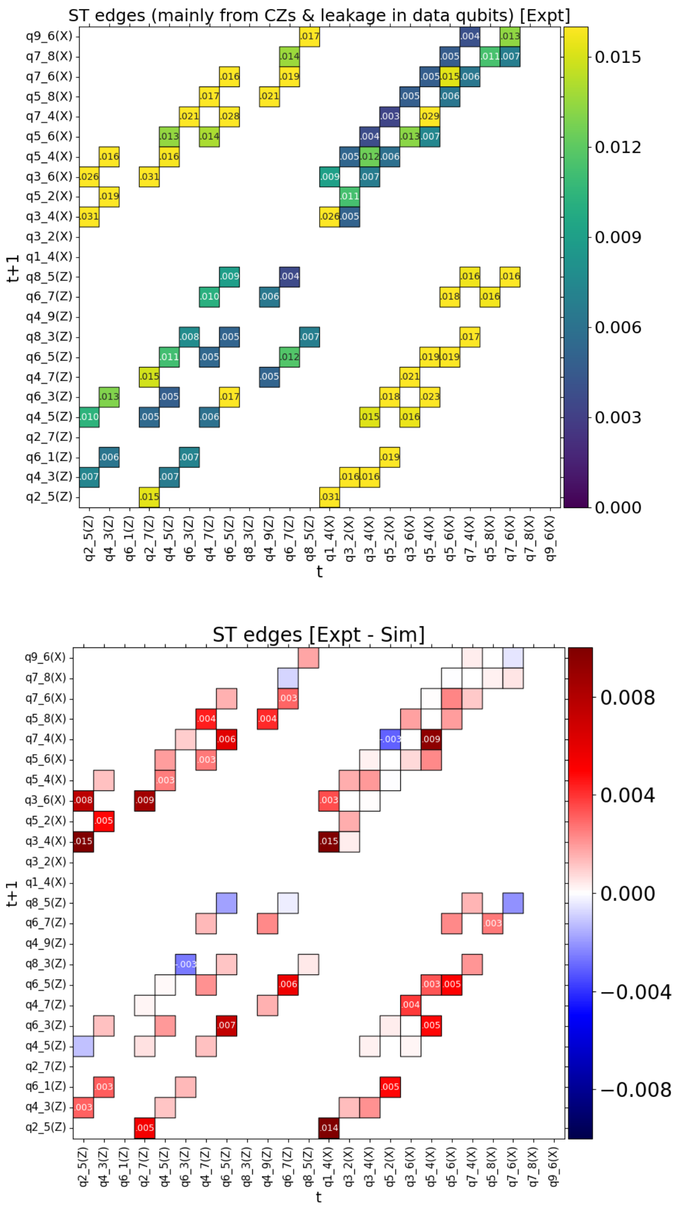

FIG. S27. Time-averaged p_{ij} probabilities of ST-edges. In the top panel, each number is the time average of ST edges from $i = (t, s_i)$ to $j = (t+1, s_j)$. We only include pairwise correlations i-j that are expected from Pauli CZ errors. In the lower panel, we show the difference of the time-averaged ST-edges between experiment and Pauli+ simulation. Numbers that are smaller in magnitude than 2.5×10^{-3} are not shown.

The top panel of Fig. S27 shows the time-averaged ST-edge probabilities for all ST edges (expected due to CZ errors) for experimental data. The lower panel shows the discrepancy between experiment and Pauli+ simulation.

The mean ST-edge probability for the distance-5 surface code experiment is

$$p_{ij}^{\mathrm{ST-edge,\,mean}} = 1.3 \times 10^{-2} \quad (29)$$
$$[\text{Pauli+ sim} = 1.1 \times 10^{-2},$$
$$\text{Pauli sim} = 0.8 \times 10^{-2}].$$

We see that the ST-edge probability that is expected from the conventional Pauli error model is 0.8×10^{-2}; however, the actual ST-edge probability is higher by 0.5×10^{-2} in the experiment and by 0.3×10^{-2} in the Pauli+ simulation. This excess of ST-edge probability turns out to be similar to the average probability of the unexpected ST'-edges:

$$p_{ij}^{\mathrm{ST'-edge,\,mean}} = 0.6 \times 10^{-2}\ [\text{Pauli+ sim} = 0.35 \times 10^{-2}].$$

A significant difference here indicates that we most likely

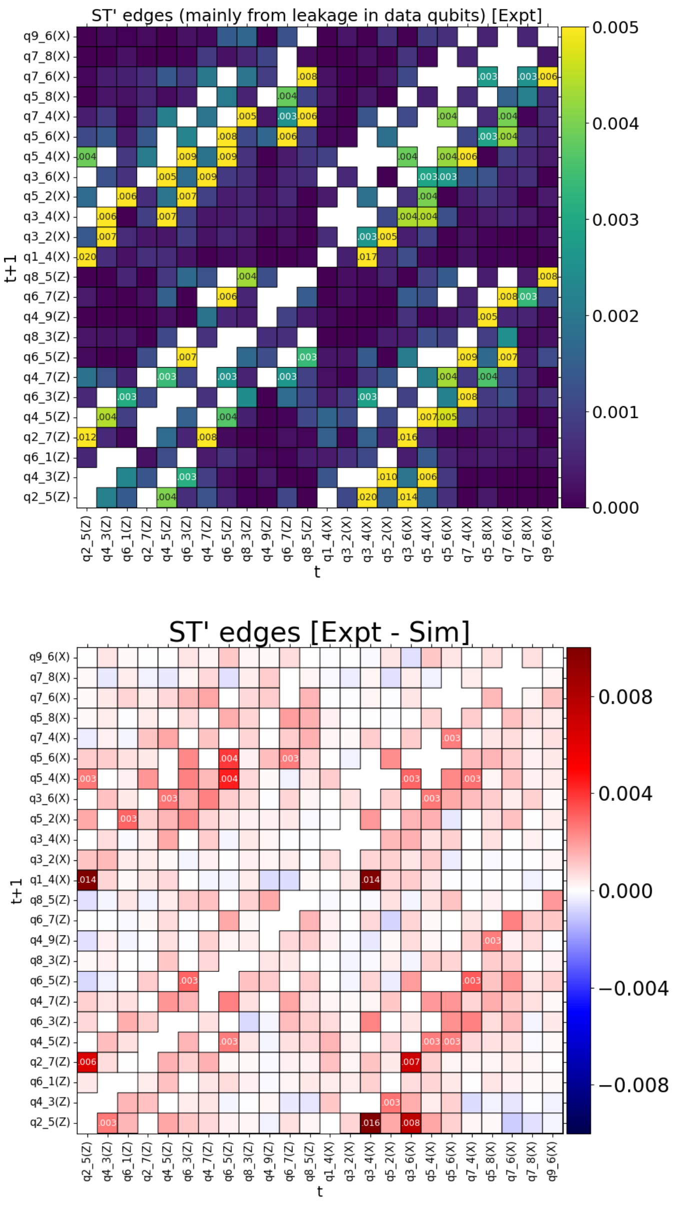

FIG. S28. Time-averaged p_{ij} probabilities of spacetimelike edges not expected from Pauli CZ errors (ST' edges). These unexpected pairwise correlations are mainly caused by leakage. Top panel shows the ST'-edge probabilities from the distance-5 experiment, and the lower panel shows the discrepancy between the experiment and the Pauli+ simulation. Numbers that are smaller in magnitude than 2.5×10^{-3} are not shown.

underestimate leakage in the simulation.

The time-averaged probability of all ST' edges for the experimental data is shown at the top panel of Fig. S28, and the lower panel shows the discrepancy between experiment and simulation. We note that the largest discrepancies are present in ST'-edges between measure qubits that are spatially close to the data qubits 2_4 and 2_6, which exhibit more leakage accumulation in the experiment than in the simulation (see lower panel of Fig. S22).

C. Probability using generalized p_{ij} of clusters of detection events

In the surface code, there are physical errors that can produce more than two detection events. To characterize such physical processes using experimental error detection data, we use a generalized version of the p_{ij} correlation matrix method [44] that allows us to calculate the probability of clusters of detection events. To explain this method, let us consider a physical error process that can produce a three-body cluster of detection events; that is, it can simultaneously flip the state of three error graph nodes, denoted as $i = 1, 2, 3$. Our goal is to find the probability of the considered error process from the experimentally accessible averages: $\langle x_1 \rangle$, $\langle x_2 \rangle$, $\langle x_3 \rangle$, $\langle x_1 x_2 \rangle$, $\langle x_1 x_3 \rangle$, $\langle x_2 x_3 \rangle$ and $\langle x_1 x_2 x_3 \rangle$. To do this, we model the statistics of the three nodes in terms of seven independent effective error processes. Three of these processes act individually on the nodes i with unknown probabilities $\tilde{p}_i$. These individual processes account for other error processes that produce detection event clusters that have only one node in common with the three-body cluster under consideration. The statistical model also includes three effective processes that separately act on the edges 1-2, 1-3 and 2-3 of the considered three-body cluster with probabilities $\tilde{p}_{12}$, $\tilde{p}_{13}$ and $\tilde{p}_{23}$. These effective processes account for the error processes that yield clusters that have two nodes in common with the considered three-body cluster. Finally, the statistical model includes the error process of interest that simultaneously flips the state of the three nodes 1, 2, 3 with a probability p_{123}. The next step is to express the above seven experimental averages in terms of the seven unknown probabilities $\tilde{p}_1$, $\tilde{p}_2$, $\tilde{p}_3$, $\tilde{p}_{12}$, $\tilde{p}_{13}$, $\tilde{p}_{23}$ and p_{123}. For instance, $\langle x_1 \rangle$ can be expressed as

$$\begin{aligned}\langle x_1 \rangle = {} & \tilde{p}_1(1-\tilde{p}_{12})(1-\tilde{p}_{13})(1-p_{123}) + (1-\tilde{p}_1)\tilde{p}_{12}\tilde{p}_{13}p_{123} \\ & + \tilde{p}_{12}(1-\tilde{p}_1)(1-\tilde{p}_{13})(1-p_{123}) + (1-\tilde{p}_{12})\tilde{p}_1\tilde{p}_{13}p_{123} \\ & + \tilde{p}_{13}(1-\tilde{p}_{12})(1-\tilde{p}_1)(1-p_{123}) + (1-\tilde{p}_{13})\tilde{p}_{12}\tilde{p}_1 p_{123} \\ & + p_{123}(1-\tilde{p}_{12})(1-\tilde{p}_{13})(1-\tilde{p}_1) + (1-p_{123})\tilde{p}_{12}\tilde{p}_{13}\tilde{p}_1.\end{aligned}$$

We assume that initially the three nodes do not exhibit detection events (i.e., $x_i = 0$). The first term describes the case in which the final state of node 1 is $x_1 = 1$ due to occurrence of the $\tilde{p}_1$-process, and no occurrence of the $\tilde{p}_{12}$-, $\tilde{p}_{13}$- and p_{123}-processes. The second term describes the case in which the final state of node 1 is $x_1 = 1$ due to occurrence of the $\tilde{p}_{12}$-, $\tilde{p}_{13}$- and p_{123}-processes, along with no occurrence of the $\tilde{p}_1$-process. The interpretation of the other terms proceeds similarly.

The final step in the calculation of the sought probability p_{123} is to solve the system of seven nonlinear equations with seven unknowns using an appropriate numeri-

Component	$p_{\rm expt}^{(i)}$	w_i	$1/\Lambda_{3/5}$ contrib.
SQ gates	1.09×10^{-3}	78.7	0.086 (9.6%)
CZ gates	4.9×10^{-3}	54.5	0.267 (29.7%)
Data qubit idle	2.46×10^{-2}	7.0	0.172 (19.1%)
Readout	1.96×10^{-2}	5.6	0.11 (12.2%)
Reset	1.86×10^{-3}	5.6	0.0104 (1.1%)
Leakage (heating)	6.6×10^{-4}	121	0.08 (8.9%)
CZ leakage	2.0×10^{-4}	121	0.024 (2.7%)
CZ crosstalk	9.5×10^{-4}	158	0.15 (16.7%)

TABLE III. Calculation of the error budget for $1/\Lambda_{3/5}$. We multiply the component errors $p_{\rm expt}^{(i)}$ by the sensitivities (weights) w_i at the half-operation point, resulting in the contributions to $1/\Lambda_{3/5}$ shown in the right column.

cal solver. This generalized p_{ij} method can be applied to analyze processes that can produce arbitrary number n of detection events. In this case, we would need to solve 2^n-1 nonlinear equations with the same number of unknown probabilities, one of which being the probability of the n-body process.

We have used the generalized p_{ij} method to do error diagnostics of higher order processes (e.g., Y data qubit errors) using the experimental error detection data. We also use the cluster probabilities provided by the generalized p_{ij} method to set the priors for decoders, as discussed in Sec. II.

We point out that if we use the p_{ij}-formula (21) on the edge between e.g. nodes 1 and 2 of the above considered three-body cluster, the p_{ij} probability would be $p_{12}=\tilde{p}_{12}(1-p_{123})+(1-\tilde{p}_{12})p_{123}\approx\tilde{p}_{12}+p_{123}$, where we have used the effective sum rule Eq. (25). This approximation is valid for sufficiently small probabilities, which are typical in our experiments. So, when we apply the p_{ij}-formula (21) on detection event data to find edge probabilities, the resulting p_{ij}-probability for a given edge i-j is approximately the sum of the probabilities of all error processes that produce detection event clusters that include the edge i-j. For this reason, the p_{ij} probabilities of e.g. spacelike edges obtained from Eq. (21) include the contribution of idle Y-type data qubit errors. If we wish to separate such errors from error processes that lead only to spacelike edges, we need to use the generalized p_{ij} method.

VII. SURFACE CODE $1/\Lambda_{3/5}$ ERROR BUDGET

In this section we discuss the procedure to obtain the surface code error budget for $1/\Lambda_{3/5}$, which is presented as the bar chart in Fig. 4a of the main text. Following Ref. [44], we write $1/\Lambda_{3/5}$ as a sum of contributions from each error channel i that is considered in the Pauli+ simulation,

$$(\Lambda_{3/5})^{-1}=\sum_i w_i\,p_{\rm expt}^{(i)}=\boldsymbol{w}\cdot\boldsymbol{p}_{\rm expt},\qquad(30)$$

where $\boldsymbol{p}_{\rm expt}$ is the vector of component error probabilities at the experimental operation point and $\boldsymbol{w}$ is the vector of weights. The latter is obtained from the gradient (sensitivities) of the nonlinear function $[1/\Lambda_{3/5}](\boldsymbol{p})$ at $\boldsymbol{p}=\boldsymbol{p}_{\rm expt}/2$, which we refer to as the "half-operation point."

The half-operation point is defined as half of the experimentally measured component error probabilities for SQ gates, CZ gates, data qubit idle, reset and readout – see Table I. Additionally, the simulation leakage parameters become $1/(1.4\,{\rm ms})$ for the heating rate and 1.0×10^{-4} for the probability of leakage generation from qubit dephasing during CZs (CZ leakage), and the crosstalk unitaries are rescaled from U_I to $(U_I)^{1/\sqrt{2}}$ at half-operation point.

The third column in Table III shows the sensitivities of $1/\Lambda_{3/5}$ at the half-operation point for the eight error channels that are considered in the simulations. These sensitivities are then used as the weight parameters w_i in Eq. (30) to obtain the contribution of each error channel to $1/\Lambda_{3/5}$. The resulting contributions are shown in the right column of Table III. Note that for the CZ error channel, we use an error probability 4.9×10^{-3} instead of 6.05×10^{-3} in Table I to avoid double counting the CZ error coming from crosstalk and CZ leakage: we subtract the contribution of 9.5×10^{-4} from crosstalk and the contribution of 2×10^{-4} from CZ leakage. Also note that for the sensitivity to CZ, leakage we use the same value as for the sensitivity to heating-induced leakage.

The values from the right column of Table III are used in Fig. 4a of the main text. The sum of these values is approximately 0.90, which is very close to the inverse of the simulated value $\Lambda_{3/5}=1.10$.

VIII. OVERVIEW OF ERROR CORRECTION EXPERIMENTS

TABLE IV. Summary of various error correction and error detection experiments. Experiments using "classical" codes (i.e. codes that only detect one type of error e.g. only phase flips or only bit flips) use $[n, k, d]$ code notation instead of quantum $[[n, k, d]]$ code notation. Entries with an N/A are experiments related to embedding error correction into the subspace of an oscillator.

Paper	Year	Code name	[[#data,#logical,distance]]	Physical qubits	Rounds	Physical qubit type
[112]	1998	Repetition Code	[3,1,3]	3	single shot	NMR
[113]	2001	Perfect Code	[[5,1,3]]	5	single shot	NMR
[114]	2011	Repetition Code	[3,1,3]	3	3	Ion trap
[115]	2011	Repetition Code	[3,1,3]	3	2	NMR
[116]	2011	Repetition Code	[3,1,3]	3	single shot	NMR
[117]	2012	Repetition Code	[3,1,3]	3	single shot	Superconducting
[118]	2012	Perfect Code	[[5,1,3]]	5	single shot	NMR
[119]	2014	Surface Code	[[4,1,2]]	4	single shot	Photons
[68]	2014	Repetition Code	[3,1,3]-[5,1,5]	9	8	Superconducting
[120]	2014	Color Code	[[7,1,3]]	7	single shot	Ion trap
[121]	2014	Repetition Code	[[3,1,3]]	4	single shot	NV center
[122]	2015	Repetition Code	[3,1,3]	5	single shot	Superconducting
[123]	2015	Bell State	[[2,0,2]]	4	single shot	Superconducting
[124]	2016	Repetition Code	[3,1,3]	4	1-3	Superconducting
[26]	2016	Cat States	N/A	1	1-6	3D cavity
[125]	2017	Color Code	[[4,2,2]]	5	single shot	Superconducting
[126]	2017	Color Code	[[4,2,2]]	5	single shot	Ion trap
[127]	2018	Repetition Code	[3,1,3]-[8,1,8]	15	single shot	Superconducting
[128]	2019	Bell State	[[2,0,2]]	3	1-12	Superconducting
[129]	2019	Perfect Code	[[5,1,3]]	5	single shot	Superconducting
[130]	2019	Binomial Bosonic States	N/A	1	1-19	3D cavity
[131]	2020	Repetition Code	[3,1,3]-[22,1,22]	5-43	single shot	Superconducting
[132]	2020	Surface Code	[[4,1,2]]	7	1-11	Superconducting
[133]	2020	Bell State	[[2,0,2]]	3	1-26	Superconducting
[20]	2020	Bacon-Shor Code	[[9,1,3]]	15	single shot	Ion trap
[134]	2020	Bacon-Shor Code	[[9,1,3]]	11	single shot	Photons
[28]	2020	GKP States	N/A	1	1-200	3D cavity
[44]	2020	Repetition Code	[3,1,3]-[11,1,11], [[4,1,2]]	5-21	1-50	Superconducting
[21]	2021	Color Code	[[7,1,3]]	10	2-12	Ion trap
[22]	2022	Perfect Code	[[5,1,3]]	7	1-11	NV center
[24]	2022	Surface Code	[[9,1,3]]	17	1-16	Superconducting
[25]	2022	Surface Code	[[9,1,3]]	17	1-11	Superconducting
[135]	2022	Surface Code	[[4,1,2]]	7	1-15	Superconducting
[23]	2022	Subsystem Code	[[9,1,3]]	23	1-10	Superconducting
[136]	2022	Surface Code	[[13, 1, 3]]	19	1	Rydberg
[136]	2022	Toric Code	[[16, 2, 2]]	24	1	Rydberg
This work	2022	Repetition Code	[3,1,3]-[25,1,25]	5-49	50	Superconducting
This work	2022	Surface Code	[[9,1,3]]-[[25,1,5]]	17-49	1-25	Superconducting

IX. ADDITIONAL PLOTS OF SURFACE CODE DATA

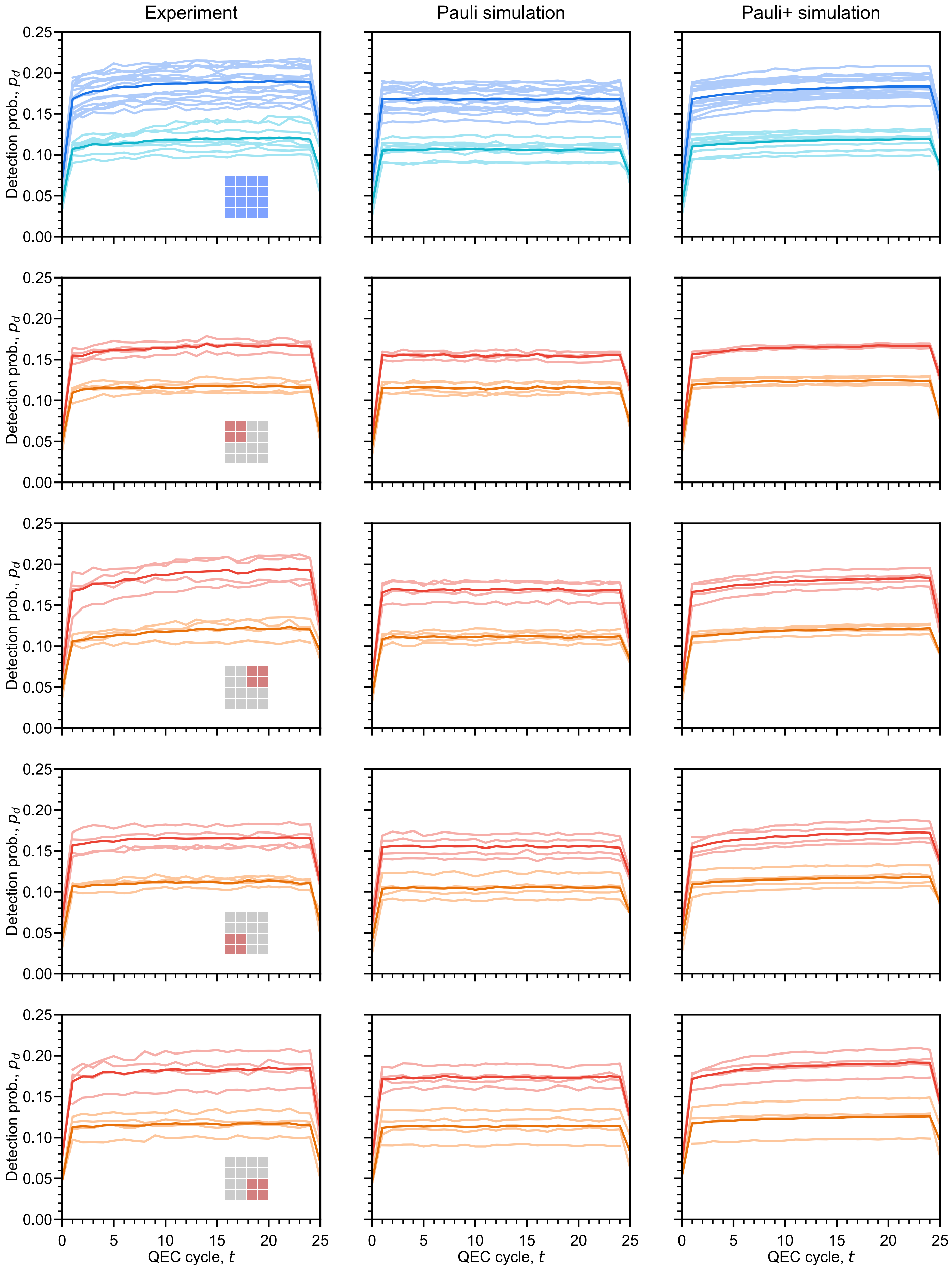

FIG. S29. Detection probabilities for individual codes.

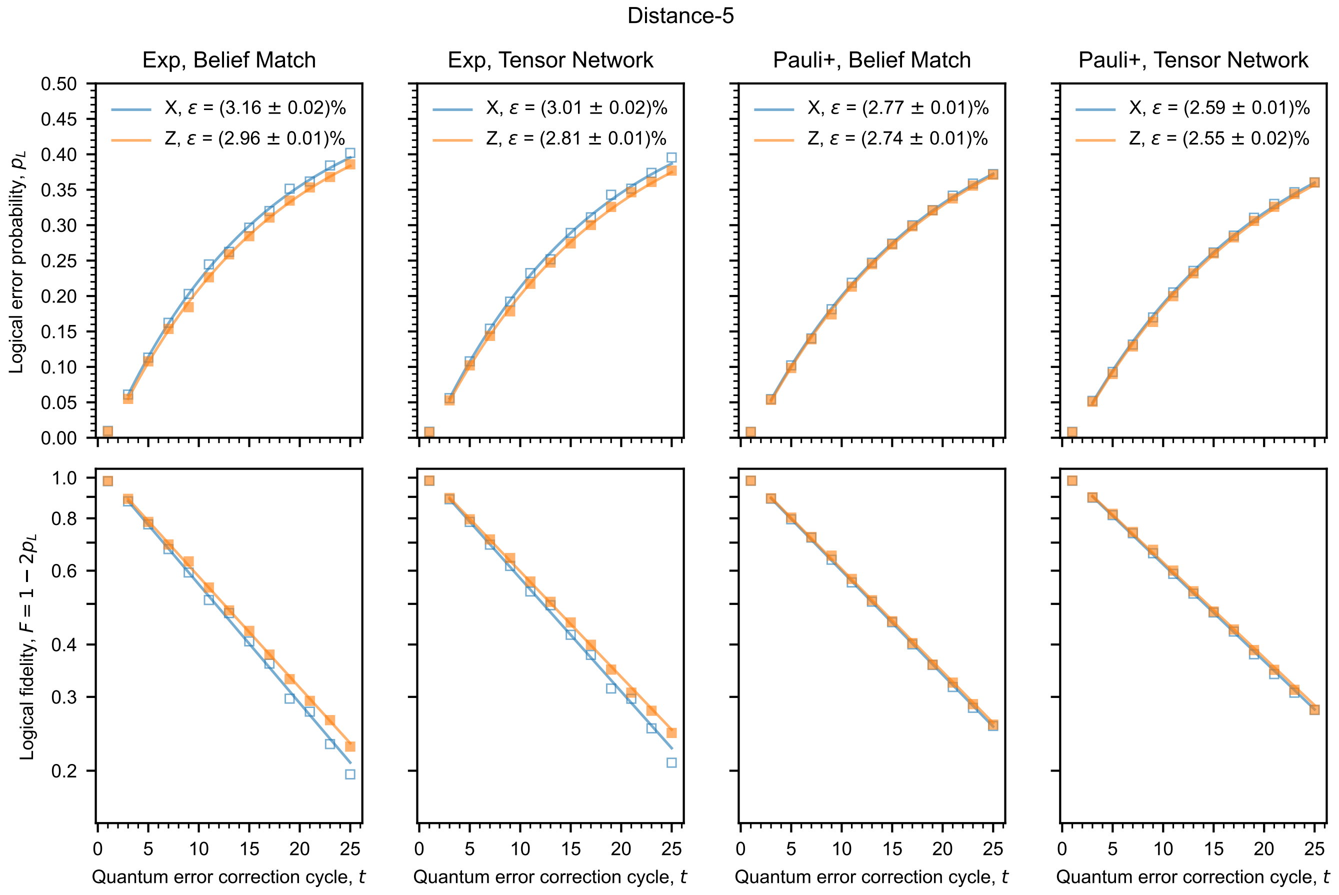

FIG. S30. Logical error probabilities vs quantum error correction cycle for the distance-5 code for four scenarios, from left to right: experimental data with belief-matching, experimental data with tensor network decoding, Pauli+ simulation with belief-matching, and Pauli+ with tensor network decoding. Top row: logical errors plotted on normal scale. Bottom row: logical fidelities plotted on logscale.

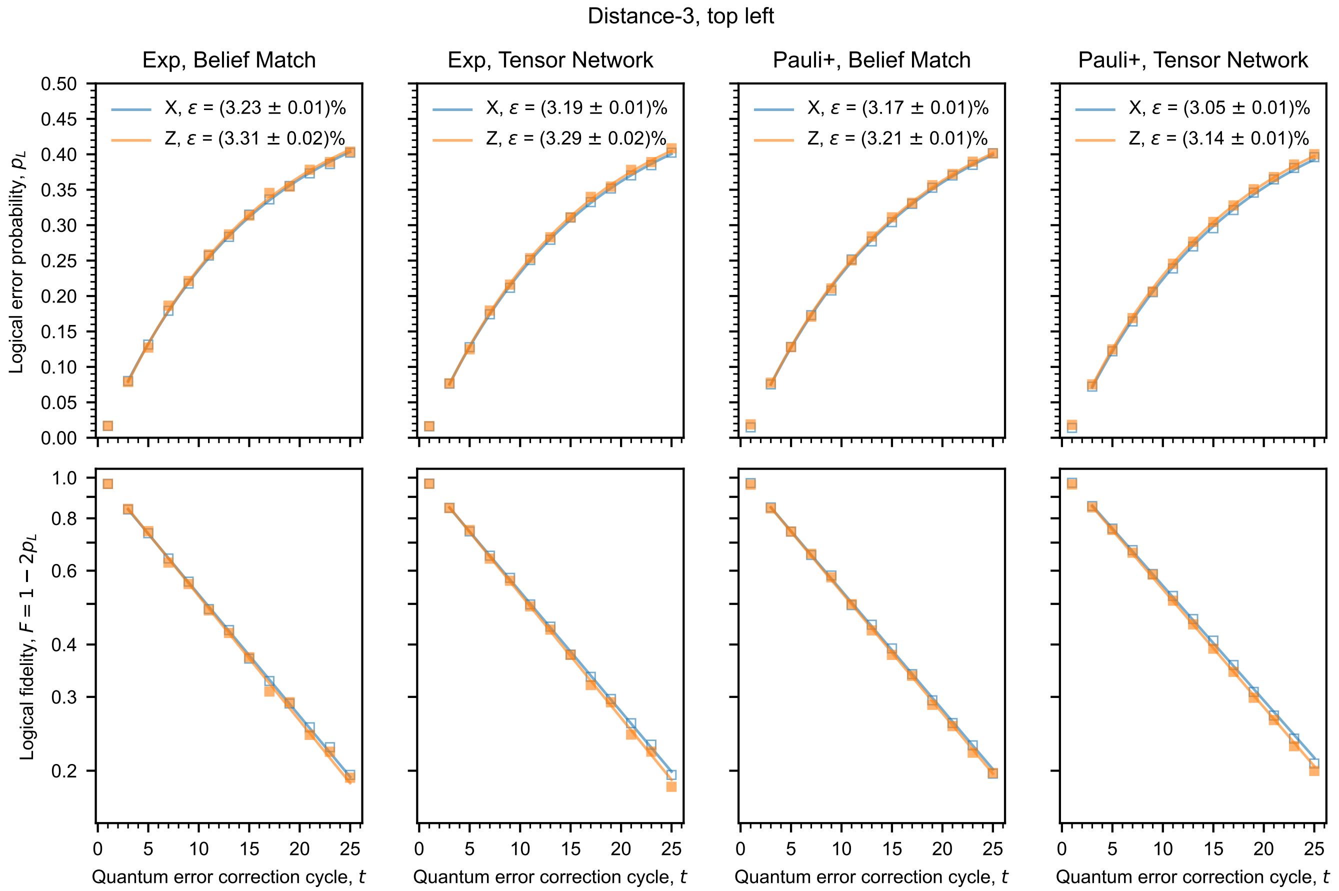

FIG. S31. Logical error probabilities vs quantum error correction cycle for the top left distance-3 code for four scenarios, from left to right: experimental data with belief-matching, experimental data with tensor network decoding, Pauli+ simulation with belief-matching, and Pauli+ with tensor network decoding. Top row: logical errors plotted on normal scale. Bottom row: logical fidelities plotted on logscale.

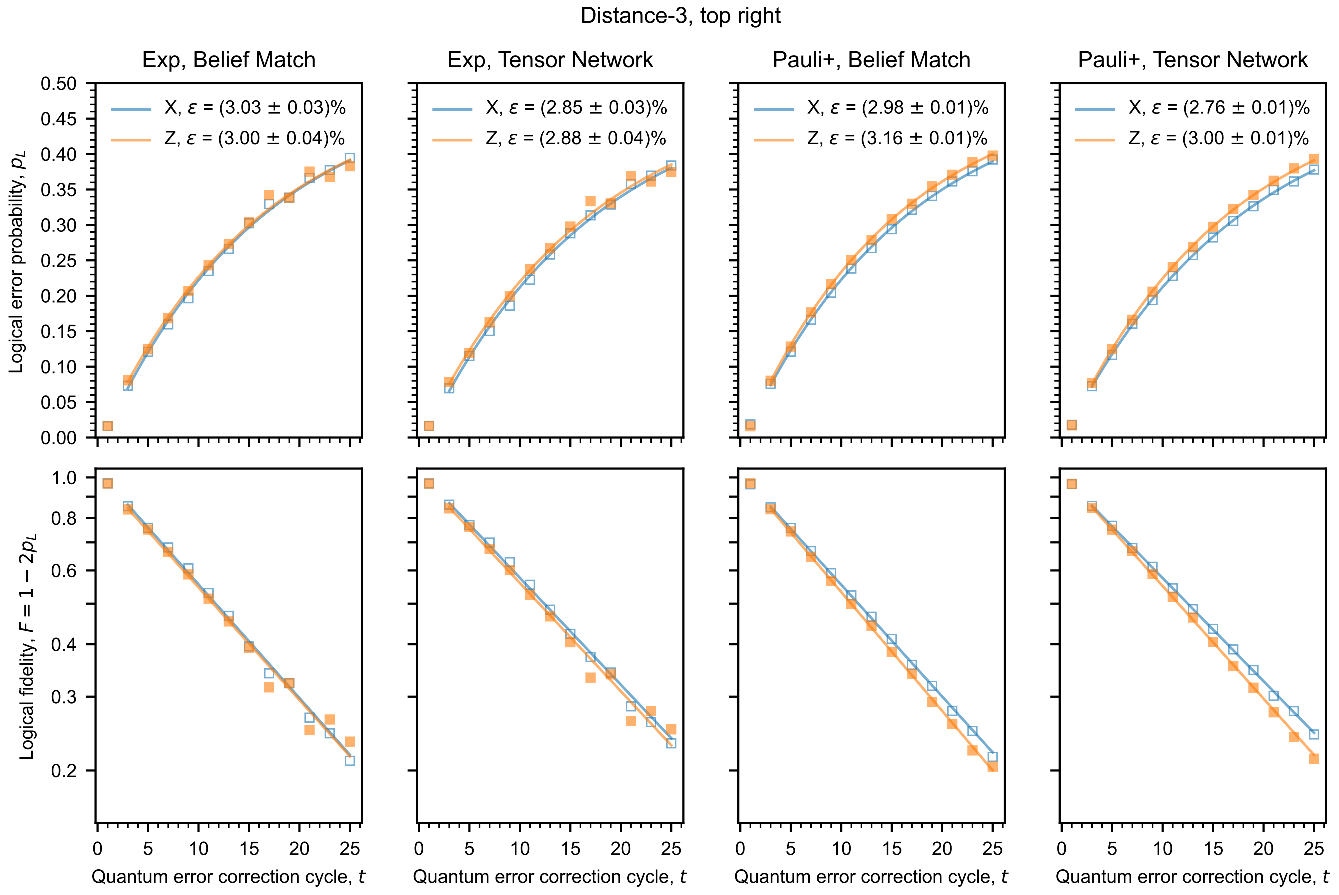

FIG. S32. Logical error probabilities vs quantum error correction cycle for the top right distance-3 code for four scenarios, from left to right: experimental data with belief-matching, experimental data with tensor network decoding, Pauli+ simulation with belief-matching, and Pauli+ with tensor network decoding. Top row: logical errors plotted on normal scale. Bottom row: logical fidelities plotted on logscale.

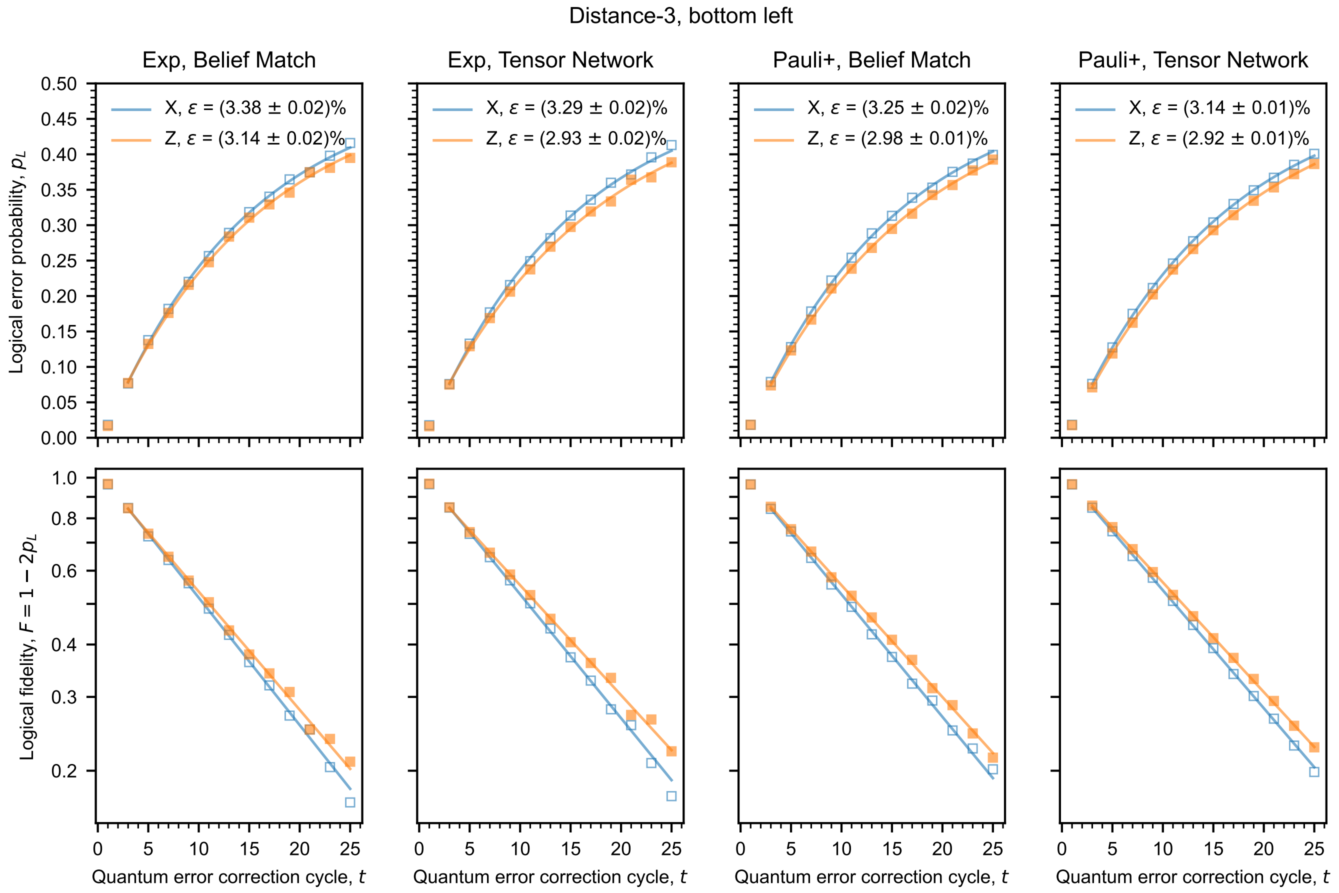

FIG. S33. Logical error probabilities vs quantum error correction cycle for the bottom left distance-3 code for four scenarios, from left to right: experimental data with belief-matching, experimental data with tensor network decoding, Pauli+ simulation with belief-matching, and Pauli+ with tensor network decoding. Top row: logical errors plotted on normal scale. Bottom row: logical fidelities plotted on logscale.

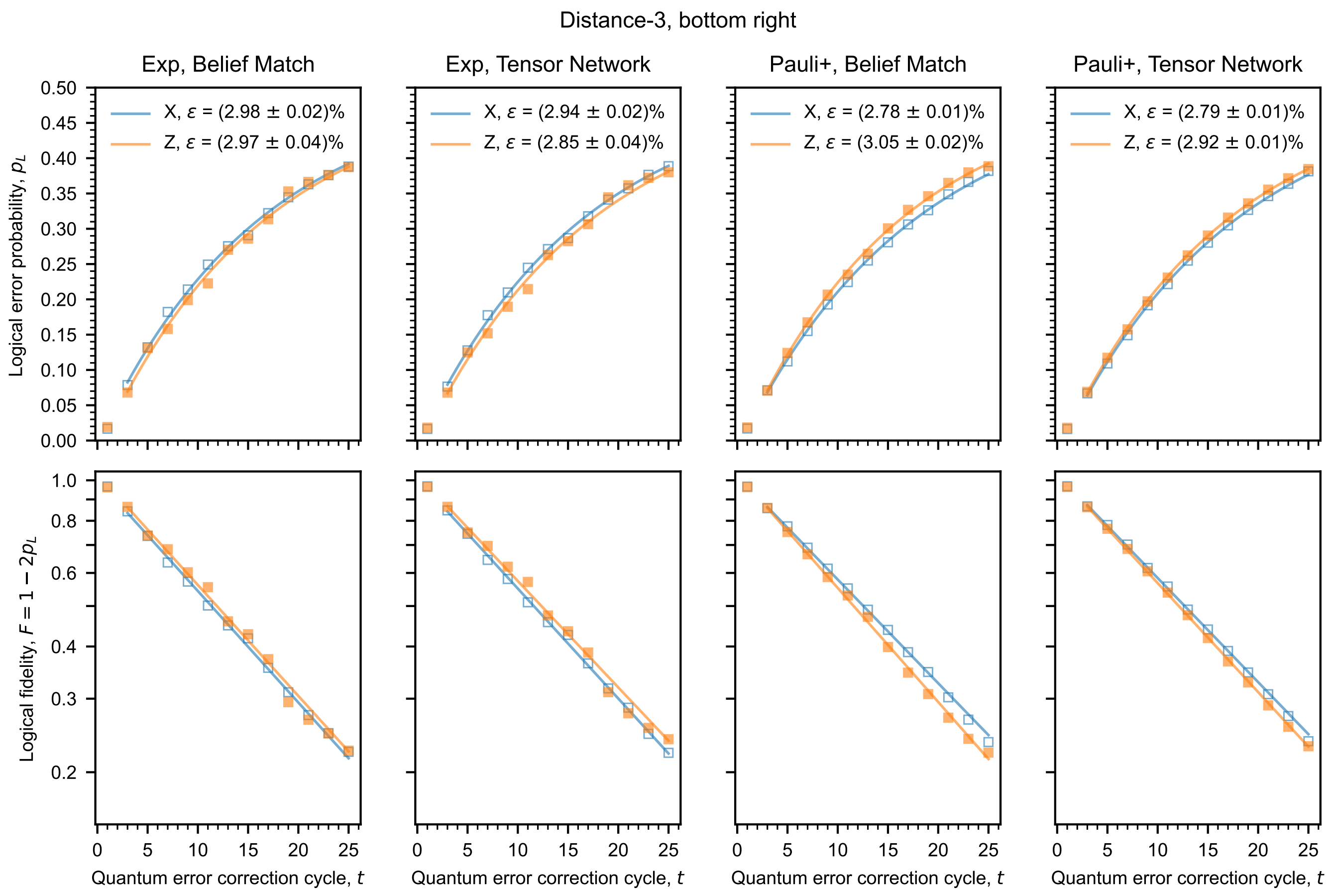

FIG. S34. Logical error probabilities vs quantum error correction cycle for the bottom right distance-3 code for four scenarios, from left to right: experimental data with belief-matching, experimental data with tensor network decoding, Pauli+ simulation with belief-matching, and Pauli+ with tensor network decoding. Top row: logical errors plotted on normal scale. Bottom row: logical fidelities plotted on logscale.

Google Quantum AI:

Rajeev Acharya[1], Igor Aleiner[1,2], Richard Allen[1], Trond I. Andersen[1], Markus Ansmann[1], Frank Arute[1], Kunal Arya[1], Abraham Asfaw[1], Juan Atalaya[1], Ryan Babbush[1], Dave Bacon[1], Joseph C. Bardin[1,3], Joao Basso[1], Andreas Bengtsson[1], Sergio Boixo[1], Gina Bortoli[1], Alexandre Bourassa[1], Jenna Bovaird[1], Leon Brill[1], Michael Broughton[1], Bob B. Buckley[1], David A. Buell[1], Tim Burger[1], Brian Burkett[1], Nicholas Bushnell[1], Yu Chen[1], Zijun Chen[1], Ben Chiaro[1], Josh Cogan[1], Roberto Collins[1], Paul Conner[1], William Courtney[1], Alexander L. Crook[1], Ben Curtin[1], Dripto M. Debroy[1], Alexander Del Toro Barba[1], Sean Demura[1], Andrew Dunsworth[1], Daniel Eppens[1], Catherine Erickson[1], Lara Faoro[1], Edward Farhi[1], Reza Fatemi[1], Leslie Flores Burgos[1], Ebrahim Forati[1], Austin G. Fowler[1], Brooks Foxen[1], William Giang[1], Craig Gidney[1], Dar Gilboa[1], Marissa Giustina[1], Alejandro Grajales Dau[1], Jonathan A. Gross[1], Steve Habegger[1], Michael C. Hamilton[1,4], Matthew P. Harrigan[1], Sean D. Harrington[1], Oscar Higgott[1], Jeremy Hilton[1], Markus Hoffmann[1], Sabrina Hong[1], Trent Huang[1], Ashley Huff[1], William J. Huggins[1], Lev B. Ioffe[1], Sergei V. Isakov[1], Justin Iveland[1], Evan Jeffrey[1], Zhang Jiang[1], Cody Jones[1], Pavol Juhas[1], Dvir Kafri[1], Kostyantyn Kechedzhi[1], Julian Kelly[1], Tanuj Khattar[1], Mostafa Khezri[1], Mária Kieferová[1,5], Seon Kim[1], Alexei Kitaev[1,6], Paul V. Klimov[1], Andrey R. Klots[1], Alexander N. Korotkov[1,7], Fedor Kostritsa[1], John Mark Kreikebaum[1], David Landhuis[1], Pavel Laptev[1], Kim-Ming Lau[1], Lily Laws[1], Joonho Lee[1], Kenny Lee[1], Brian J. Lester[1], Alexander Lill[1], Wayne Liu[1], Aditya Locharla[1], Erik Lucero[1], Fionn D. Malone[1], Jeffrey Marshall[8,9], Orion Martin[1], Jarrod R. McClean[1], Trevor McCourt[1], Matt McEwen[1,10], Anthony Megrant[1], Bernardo Meurer Costa[1], Xiao Mi[1], Kevin C. Miao[1], Masoud Mohseni[1], Shirin Montazeri[1], Alexis Morvan[1], Emily Mount[1], Wojciech Mruczkiewicz[1], Ofer Naaman[1], Matthew Neeley[1], Charles Neill[1], Ani Nersisyan[1], Hartmut Neven[1], Michael Newman[1], Jiun How Ng[1], Anthony Nguyen[1], Murray Nguyen[1], Murphy Yuezhen Niu[1], Thomas E. O'Brien[1], Alex Opremcak[1], John Platt[1], Andre Petukhov[1], Rebecca Potter[1], Leonid P. Pryadko[11,1], Chris Quintana[1], Pedram Roushan[1], Nicholas C. Rubin[1], Negar Saei[1], Daniel Sank[1], Kannan Sankaragomathi[1], Kevin J. Satzinger[1], Henry F. Schurkus[1], Christopher Schuster[1], Michael J. Shearn[1], Aaron Shorter[1], Vladimir Shvarts[1], Jindra Skruzny[1], Vadim Smelyanskiy[1], W. Clarke Smith[1], George Sterling[1], Doug Strain[1], Marco Szalay[1], Alfredo Torres[1], Guifre Vidal[1], Benjamin Villalonga[1], Catherine Vollgraff Heidweiller[1], Theodore White[1], Cheng Xing[1], Z. Jamie Yao[1], Ping Yeh[1], Juhwan Yoo[1], Grayson Young[1], Adam Zalcman[1], Yaxing Zhang[1], Ningfeng Zhu[1]

[1] Google Research
[2] Department of Physics, Columbia University, New York, NY
[3] Department of Electrical and Computer Engineering, University of Massachusetts, Amherst, MA
[4] Department of Electrical and Computer Engineering, Auburn University, Auburn, AL
[5] Centre for Quantum Computation and Communication Technology, Centre for Quantum Software and Information, Faculty of Engineering and Information Technology, University of Technology Sydney, NSW 2007, Australia
[6] Department of Physics, Institute for Quantum Information and Matter, and Walter Burke Institute for Theoretical Physics, California Institute of Technology, Pasadena, CA
[7] Department of Electrical and Computer Engineering, University of California, Riverside, CA
[8] USRA Research Institute for Advanced Computer Science, Mountain View, CA
[9] QuAIL, NASA Ames Research Center, Moffett Field, CA
[10] Department of Physics, University of California, Santa Barbara, CA
[11] Department of Physics and Astronomy, University of California, Riverside, CA

---

[1] Shor, P. W. Scheme for reducing decoherence in quantum computer memory. *Physical Review A* **52**, R2493 (1995).

[2] Gottesman, D. *Stabilizer codes and quantum error correction*. Ph.D. thesis, California Institute of Technology, Pasadena, CA (1997).

[3] Feynman, R. P. Simulating physics with computers. *International Journal of Theoretical Physics* **21** (1982).

[4] Shor, P. W. Polynomial-time algorithms for prime factorization and discrete logarithms on a quantum computer. *SIAM review* **41**, 303–332 (1999).

[5] Farhi, E. *et al.* A quantum adiabatic evolution algorithm applied to random instances of an NP-complete problem. *Science* **292**, 472–475 (2001).

[6] Biamonte, J. *et al.* Quantum machine learning. *Nature* **549**, 195–202 (2017).

[7] Lloyd, S. Universal quantum simulators. *Science* **273**, 1073–1078 (1996).

[8] Aspuru-Guzik, A., Dutoi, A. D., Love, P. J. & Head-Gordon, M. Simulated quantum computation of molecular energies. *Science* **309**, 1704–1707 (2005).

[9] Reiher, M., Wiebe, N., Svore, K. M., Wecker, D. & Troyer, M. Elucidating reaction mechanisms on quantum computers. *Proceedings of the National Academy of Sciences* **114**, 7555–7560 (2017). URL `https://www.pnas.org/doi/abs/10.1073/pnas.1619152114`.

[10] Gidney, C. & Ekera, M. How to factor 2048 bit RSA integers in 8 hours using 20 million noisy qubits. *Quantum* **5**, 433 (2021).

[11] Kivlichan, I. D. *et al.* Improved fault-tolerant quantum simulation of condensed-phase correlated electrons via trotterization. *Quantum* **4**, 296 (2020).

[12] Ballance, C., Harty, T., Linke, N., Sepiol, M. & Lucas, D. High-fidelity quantum logic gates using trapped-ion hyperfine qubits. *Physical Review Letters* **117**, 060504 (2016).

[13] Huang, W. *et al.* Fidelity benchmarks for two-qubit gates in silicon. *Nature* **569**, 532–536 (2019).

[14] Rol, M. *et al.* Fast, high-fidelity conditional-phase gate exploiting leakage interference in weakly anharmonic superconducting qubits. *Physical Review Letters* **123**, 120502 (2019).

[15] Jurcevic, P. *et al.* Demonstration of quantum volume 64 on a superconducting quantum computing system. *arXiv preprint arXiv:2008.08571* (2020).

[16] Foxen, B. *et al.* Demonstrating a continuous set of two-qubit gates for near-term quantum algorithms. *Physical Review Letters* **125**, 120504 (2020).

[17] Wu, Y. *et al.* Strong quantum computational advantage using a superconducting quantum processor. *Phys. Rev. Lett.* **127**, 180501 (2021). URL `https://link.aps.org/doi/10.1103/PhysRevLett.127.180501`.

[18] Knill, E., Laflamme, R. & Zurek, W. H. Resilient quantum computation. *Science* **279**, 342–345 (1998).

[19] Aharonov, D. & Ben-Or, M. Fault-tolerant quantum computation with constant error rate. *SIAM Journal on Computing* (2008).

[20] Egan, L. *et al.* Fault-tolerant control of an error-corrected qubit. *Nature* **598**, 281–286 (2021).

[21] Ryan-Anderson, C. *et al.* Realization of real-time fault-tolerant quantum error correction. *Physical Review X* **11**, 041058 (2021).

[22] Abobeih, M. *et al.* Fault-tolerant operation of a logical qubit in a diamond quantum processor. *Nature* **606**, 884–889 (2022).

[23] Sundaresan, N. *et al.* Matching and maximum likelihood decoding of a multi-round subsystem quantum error correction experiment. *arXiv preprint arXiv:2203.07205* (2022).

[24] Krinner, S. *et al.* Realizing repeated quantum error correction in a distance-three surface code. *Nature* **605**, 669–674 (2022).

[25] Zhao, Y. *et al.* Realization of an error-correcting surface code with superconducting qubits. *Phys. Rev. Lett.* **129**, 030501 (2022). URL `https://link.aps.org/doi/10.1103/PhysRevLett.129.030501`.

[26] Ofek, N. *et al.* Extending the lifetime of a quantum bit with error correction in superconducting circuits. *Nature* **536**, 441–445 (2016).

[27] Flühmann, C. *et al.* Encoding a qubit in a trapped-ion mechanical oscillator. *Nature* **566**, 513–517 (2019).

[28] Campagne-Ibarcq, P. *et al.* Quantum error correction of a qubit encoded in grid states of an oscillator. *Nature* **584**, 368–372 (2020).

[29] Grimm, A. *et al.* Stabilization and operation of a Kerr-cat qubit. *Nature* **584**, 205–209 (2020).

[30] Emerson, J., Alicki, R. & Życzkowski, K. Scalable noise estimation with random unitary operators. *Journal of Optics B: Quantum and Semiclassical Optics* **7**, S347 (2005).

[31] Arute, F. *et al.* Quantum supremacy using a programmable superconducting processor. *Nature* **574**, 505–510 (2019).

[32] See supplementary information.

[33] Kitaev, A. Y. Fault-tolerant quantum computation by anyons. *Annals of Physics* **303**, 2–30 (2003).

[34] Dennis, E., Kitaev, A., Landahl, A. & Preskill, J. Topological quantum memory. *Journal of Mathematical Physics* **43**, 4452–4505 (2002). URL `https://doi.org/10.1063/1.1499754`.

[35] Raussendorf, R. & Harrington, J. Fault-tolerant quantum computation with high threshold in two dimensions. *Physical Review Letters* **98**, 190504 (2007).

[36] Fowler, A. G., Mariantoni, M., Martinis, J. M. & Cleland, A. N. Surface codes: Towards practical large-scale quantum computation. *Physical Review A* **86**, 032324 (2012).

[37] Satzinger, K. *et al.* Realizing topologically ordered states on a quantum processor. *Science* **374**, 1237–1241 (2021).

[38] Horsman, C., Fowler, A. G., Devitt, S. & Meter, R. V. Surface code quantum computing by lattice surgery. *New Journal of Physics* **14**, 123011 (2012).

[39] Fowler, A. G. & Gidney, C. Low overhead quantum computation using lattice surgery. *arXiv preprint arXiv:1808.06709* (2018).

[40] Litinski, D. A game of surface codes: Large-scale quantum computing with lattice surgery. *Quantum* **3**, 128 (2019).

[41] Koch, J. *et al.* Charge-insensitive qubit design derived from the Cooper pair box. *Physical Review A* **76**, 042319 (2007).

[42] C. Neill. *A path towards quantum supremacy with superconducting qubits.* Ph.D. thesis, University of California Santa Barbara, Santa Barbara, CA (2017).

[43] Yan, F. *et al.* Tunable coupling scheme for implementing high-fidelity two-qubit gates. *Phys. Rev. Applied* **10**, 054062 (2018). URL `https://link.aps.org/doi/10.1103/PhysRevApplied.10.054062`.

[44] Chen, Z. *et al.* Exponential suppression of bit or phase errors with cyclic error correction. *Nature* **595**, 383–387 (2021).

[45] Kelly, J. *et al.* Scalable in situ qubit calibration during repetitive error detection. *Physical Review A* **94**, 032321 (2016).

[46] Wen, X.-G. Quantum orders in an exact soluble model. *Physical Review Letters* **90**, 016803 (2003).

[47] Bonilla Ataides, J. P., Tuckett, D. K., Bartlett, S. D., Flammia, S. T. & Brown, B. J. The XZZX surface code. *Nature Communications* **12**, 1–12 (2021).

[48] Aliferis, P. & Terhal, B. M. Fault-tolerant quantum computation for local leakage faults. *Quantum Info. Comput.* **7**, 139–156 (2007).

[49] Suchara, M., Cross, A. W. & Gambetta, J. M. Leakage suppression in the toric code. In *2015 IEEE International Symposium on Information Theory (ISIT)*, 1119–1123 (IEEE, 2015).

[50] McEwen, M. *et al.* Removing leakage-induced correlated errors in superconducting quantum error correction. *Nature Communications* **12**, 1–7 (2021).

[51] Spitz, S. T., Tarasinski, B., Beenakker, C. W. & O'Brien, T. E. Adaptive weight estimator for quantum error correction in a time-dependent environment. *Advanced Quantum Technologies* **1**, 1800012 (2018).

[52] Chen, E. H. *et al.* Calibrated decoders for experimental quantum error correction. *Physical Review Letters* **128**, 110504 (2022).

[53] Higgott, O., Bohdanowicz, T. C., Kubica, A., Flammia, S. T. & Campbell, E. T. Fragile boundaries of tailored surface codes and improved decoding of circuit-level noise. *arXiv preprint arXiv:2203.04948* (2022).

[54] Criger, B. & Ashraf, I. Multi-path summation for decoding 2D topological codes. *Quantum* **2**, 102 (2018).

[55] Fowler, A. G., Whiteside, A. C. & Hollenberg, L. C. Towards practical classical processing for the surface code. *Physical Review Letters* **108**, 180501 (2012).

[56] Bravyi, S., Suchara, M. & Vargo, A. Efficient algorithms for maximum likelihood decoding in the surface code. *Phys. Rev. A* **90**, 032326 (2014). URL `https://link.aps.org/doi/10.1103/PhysRevA.90.032326`.

[57] Chubb, C. T. & Flammia, S. T. Statistical mechanical models for quantum codes with correlated noise. *Annales de l'Institut Henri Poincaré D* **8**, 269–321 (2021).

[58] Pattison, C. A., Beverland, M. E., da Silva, M. P. & Delfosse, N. Improved quantum error correction using soft information. *arXiv preprint arXiv:2107.13589* (2021).

[59] McEwen, M. *et al.* Resolving catastrophic error bursts from cosmic rays in large arrays of superconducting qubits. *Nature Physics* **18**, 107–111 (2022).

[60] Stephens, A. M. Fault-tolerant thresholds for quantum error correction with the surface code. *Physical Review A* **89**, 022321 (2014).

[61] Gullion, T., Baker, D. B. & Conradi, M. S. New, compensated carr-purcell sequences. *Journal of Magnetic Resonance* **89**, 479–484 (1990).

[62] Klimov, P. V., Kelly, J., Martinis, J. M. & Neven, H. The snake optimizer for learning quantum processor control parameters. *arXiv:2006.04594* (2020). URL `https://arxiv.org/abs/2006.04594`.

[63] Klimov, P. V. *et al.* Fluctuations of energy-relaxation times in superconducting qubits. *Phys. Rev. Lett.* **121**, 090502 (2018). URL `https://link.aps.org/doi/10.1103/PhysRevLett.121.090502`.

[64] Mutus, J. Y. *et al.* Strong environmental coupling in a josephson parametric amplifier. *Applied Physics Letters* **104**, 263513 (2014). URL `https://doi.org/10.1063/1.4886408`. https://doi.org/10.1063/1.4886408.

[65] Sank, D. *et al.* Measurement-induced state transitions in a superconducting qubit: Beyond the rotating wave approximation. *Physical Review Letters* **117**, 190503 (2016).

[66] Vijay, R. *et al.* Stabilizing Rabi oscillations in a superconducting qubit using quantum feedback. *Nature* **490**, 77–80 (2012).

[67] Bultink, C. C. *et al.* General method for extracting the quantum efficiency of dispersive qubit readout in circuit QED. *Applied Physics Letters* **112**, 092601 (2018).

[68] Kelly, J. *et al.* State preservation by repetitive error detection in a superconducting quantum circuit. *Nature* **519**, 66–69 (2015).

[69] Gidney, C. Stim: a fast stabilizer circuit simulator. *Quantum* **5**, 497 (2021). URL `https://doi.org/10.22331/q-2021-07-06-497`.

[70] Fowler, A. G. Optimal complexity correction of correlated errors in the surface code. *arXiv preprint arXiv:1310.0863* (2013).

[71] Zhang, J. & Fossorier, M. Shuffled iterative decoding. *IEEE Transactions on Communications* **53**, 209–213 (2005).

[72] Panteleev, P. & Kalachev, G. Degenerate Quantum LDPC Codes With Good Finite Length Performance. *Quantum* **5**, 585 (2021). URL `https://doi.org/10.22331/q-2021-11-22-585`.

[73] Kuo, K.-Y. & Lai, C.-Y. Refined belief propagation decoding of sparse-graph quantum codes. *IEEE Journal on Selected Areas in Information Theory* **1**, 487–498 (2020).

[74] Poulin, D. & Chung, Y. On the iterative decoding of sparse quantum codes. *Quantum Info. Comput.* **8**, 987–1000 (2008).

[75] Higgott, O. & Breuckmann, N. P. Improved single-shot decoding of higher dimensional hypergraph product codes. *arXiv preprint arXiv:2206.03122* (2022).

[76] Chen, J., Dholakia, A., Eleftheriou, E., Fossorier, M. & Hu, X.-Y. Reduced-complexity decoding of LDPC codes. *IEEE Transactions on Communications* **53**, 1288–1299 (2005).

[77] Huang, S., Newman, M. & Brown, K. R. Fault-tolerant weighted union-find decoding on the toric code. *Phys. Rev. A* **102**, 012419 (2020). URL `https://link.aps.org/doi/10.1103/PhysRevA.102.012419`.

[78] Delfosse, N. & Nickerson, N. H. Almost-linear time decoding algorithm for topological codes. *Quantum* **5**, 595 (2021). URL `https://doi.org/10.22331/q-2021-12-02-595`.

[79] Gray, J. quimb: A python package for quantum information and many-body calculations. *Journal of Open Source Software* **3**, 819 (2018).

[80] Tomita, Y. & Svore, K. M. Low-distance surface codes under realistic quantum noise. *Physical Review A* **90**, 062320 (2014).

[81] Isakov, S. V. *et al.* Simulations of quantum circuits with approximate noise using qsim and cirq. *arXiv preprint arXiv:2111.02396* (2021).

[82] Geller, M. R. & Zhou, Z. Efficient error models for fault-tolerant architectures and the Pauli twirling approximation. *Physical Review A* **88**, 012314 (2013).

[83] Chen, Z. *et al.* Measuring and suppressing quantum state leakage in a superconducting qubit. *Physical review letters* **116**, 020501 (2016).

[84] Choi, M.-D. Completely positive linear maps on complex matrices. *Linear algebra and its applications* **10**, 285–290 (1975).

[85] Verstraete, F. & Verschelde, H. On quantum channels. *arXiv preprint quant-ph/0202124* (2002).

[86] Battistel, F., Varbanov, B. M. & Terhal, B. M. Hardware-efficient leakage-reduction scheme for quantum error correction with superconducting transmon qubits. *PRX Quantum* **2**, 030314 (2021).

[87] Sung, Y. *et al.* Realization of high-fidelity CZ and ZZ-free iSWAP gates with a tunable coupler. *Phys. Rev. X* **11**, 021058 (2021). URL `https://link.aps.org/doi/10.1103/PhysRevX.11.021058`.

[88] Gilchrist, A., Langford, N. K. & Nielsen, M. A. Distance measures to compare real and ideal quantum processes. *Physical Review A* **71**, 062310 (2005).

[89] Pedersen, L. H., Møller, N. M. & Mølmer, K. Fidelity of quantum operations. *Physics Letters A* **367**, 47–51 (2007).

[90] Quantum AI team & collaborators. qsim (2020). URL `https://doi.org/10.5281/zenodo.4023103`.

[91] Wilde, M. M. *Quantum information theory* (Cambridge University Press, 2013).

[92] O'Brien, T., Tarasinski, B. & DiCarlo, L. Density-matrix simulation of small surface codes under current and projected experimental noise. *npj Quantum Information* **3**, 39 (2017).

[93] Varbanov, B. M. *et al.* Leakage detection for a transmon-based surface code. *npj Quantum Information* **6**, 102 (2020).

[94] Gottesman, D. The Heisenberg representation of quantum computers. In Corney, S. P., Delbourgo, R. & Jarvis, P. D. (eds.) *Proceedings of the XXII Inter-*

national Colloquium on Group Theoretical Methods in Physics, 32–43 (International Press, Cambridge, MA, 1999).

[95] Steane, A. M. Overhead and noise threshold of fault-tolerant quantum error correction. *Phys. Rev. A* **68**, 042322 (2003). URL `http://dx.doi.org/10.1103/PhysRevA.68.042322`.

[96] Fowler, A. G., Hill, C. D. & Hollenberg, L. C. L. Quantum-error correction on linear-nearest-neighbor qubit arrays. *Phys. Rev. A* **69**, 042314 (2004). URL `http://link.aps.org/abstract/PRA/v69/e042314`.

[97] Raussendorf, R. & Harrington, J. Fault-tolerant quantum computation with high threshold in two dimensions. *Phys. Rev. Lett.* **98**, 190504 (2007). URL `http://link.aps.org/abstract/PRL/v98/e190504`.

[98] Wang, D. S., Fowler, A. G., Stephens, A. M. & Hollenberg, L. C. L. Threshold error rates for the toric and planar codes. *Quantum Inf. Comput.* **10**, 456–469 (2010).

[99] Wang, D. S., Fowler, A. G. & Hollenberg, L. C. L. Surface code quantum computing with error rates over 1%. *Phys. Rev. A* **83**, 020302 (2011). URL `http://link.aps.org/doi/10.1103/PhysRevA.83.020302`.

[100] Landahl, A. J., Anderson, J. T. & Rice, P. R. Fault-tolerant quantum computing with color codes (2011). Presented at QIP 2012, December 12 to December 16.

[101] Brown, B. J., Nickerson, N. H. & Browne, D. E. Fault-tolerant error correction with the gauge color code. *Nature Communications* **7**, 12302 (2016). URL `https://doi.org/10.1038/ncomms12302`.

[102] Tuckett, D. K., Bartlett, S. D. & Flammia, S. T. Ultrahigh error threshold for surface codes with biased noise. *Phys. Rev. Lett.* **120**, 050505 (2018). URL `https://link.aps.org/doi/10.1103/PhysRevLett.120.050505`.

[103] Gutiérrez, M., Svec, L., Vargo, A. & Brown, K. R. Approximation of realistic errors by Clifford channels and Pauli measurements. *Phys. Rev. A* **87**, 030302 (2013). URL `https://link.aps.org/doi/10.1103/PhysRevA.87.030302`.

[104] Bravyi, S. & Gosset, D. Improved classical simulation of quantum circuits dominated by Clifford gates. *Phys. Rev. Lett.* **116**, 250501 (2016). URL `https://link.aps.org/doi/10.1103/PhysRevLett.116.250501`.

[105] Bravyi, S. *et al.* Simulation of quantum circuits by low-rank stabilizer decompositions. *Quantum* **3**, 181 (2019).

[106] Aaronson, S. & Gottesman, D. Improved simulation of stabilizer circuits. *Physical Review A* **70**, 052328 (2004).

[107] Fowler, A. G. Coping with qubit leakage in topological codes. *Physical Review A* **88**, 042308 (2013).

[108] Cai, Z. & Benjamin, S. C. Constructing smaller Pauli twirling sets for arbitrary error channels. *Scientific reports* **9**, 1–11 (2019).

[109] Bravyi, S., Englbrecht, M., König, R. & Peard, N. Correcting coherent errors with surface codes. *NPJ Quantum Information* **4**, 55 (2018). URL `https://doi.org/10.1038/s41534-018-0106-y`.

[110] Katabarwa, A. & Geller, M. R. Logical error rate in the Pauli twirling approximation. *Scientific reports* **5**, 1–6 (2015).

[111] Zhao, P. *et al.* Quantum crosstalk analysis for simultaneous gate operations on superconducting qubits. *PRX Quantum* **3**, 020301 (2022). URL `https://link.aps.org/doi/10.1103/PRXQuantum.3.020301`.

[112] Cory, D. G. *et al.* Experimental quantum error correction. *Physical Review Letters* **81**, 2152 (1998).

[113] Knill, E., Laflamme, R., Martinez, R. & Negrevergne, C. Benchmarking quantum computers: the five-qubit error correcting code. *Physical Review Letters* **86**, 5811 (2001).

[114] Schindler, P. *et al.* Experimental repetitive quantum error correction. *Science* **332**, 1059–1061 (2011).

[115] Moussa, O., Baugh, J., Ryan, C. A. & Laflamme, R. Demonstration of sufficient control for two rounds of quantum error correction in a solid state ensemble quantum information processor. *Physical Review Letters* **107**, 160501 (2011).

[116] Zhang, J., Gangloff, D., Moussa, O. & Laflamme, R. Experimental quantum error correction with high fidelity. *Physical Review A* **84**, 034303 (2011).

[117] Reed, M. D. *et al.* Realization of three-qubit quantum error correction with superconducting circuits. *Nature* **482**, 382–385 (2012).

[118] Zhang, J., Laflamme, R. & Suter, D. Experimental implementation of encoded logical qubit operations in a perfect quantum error correcting code. *Physical Review Letters* **109**, 100503 (2012).

[119] Bell, B. *et al.* Experimental demonstration of a graph state quantum error-correction code. *Nature Communications* **5**, 1–10 (2014).

[120] Nigg, D. *et al.* Quantum computations on a topologically encoded qubit. *Science* **345**, 302–305 (2014).

[121] Waldherr, G. *et al.* Quantum error correction in a solid-state hybrid spin register. *Nature* **506**, 204–207 (2014).

[122] Riste, D. *et al.* Detecting bit-flip errors in a logical qubit using stabilizer measurements. *Nature Communications* **6**, 1–6 (2015).

[123] Córcoles, A. D. *et al.* Demonstration of a quantum error detection code using a square lattice of four superconducting qubits. *Nature Communications* **6**, 1–10 (2015).

[124] Cramer, J. *et al.* Repeated quantum error correction on a continuously encoded qubit by real-time feedback. *Nature Communications* **7**, 1–7 (2016).

[125] Takita, M., Cross, A. W., Córcoles, A., Chow, J. M. & Gambetta, J. M. Experimental demonstration of fault-tolerant state preparation with superconducting qubits. *Physical Review Letters* **119**, 180501 (2017).

[126] Linke, N. M. *et al.* Fault-tolerant quantum error detection. *Science Advances* **3**, e1701074 (2017).

[127] Wootton, J. R. & Loss, D. Repetition code of 15 qubits. *Physical Review A* **97**, 052313 (2018).

[128] Andersen, C. K. *et al.* Entanglement stabilization using ancilla-based parity detection and real-time feedback in superconducting circuits. *npj Quantum Information* **5**, 1–7 (2019).

[129] Gong, M. *et al.* Experimental exploration of five-qubit quantum error-correcting code with superconducting qubits. *National Science Review* **9** (2021). URL `https://doi.org/10.1093/nsr/nwab011`.

[130] Hu, L. *et al.* Quantum error correction and universal gate set operation on a binomial bosonic logical qubit. *Nature Physics* **15**, 503–508 (2019).

[131] Wootton, J. R. Benchmarking near-term devices with quantum error correction. *Quantum Science and Technology* **5**, 044004 (2020).

[132] Andersen, C. K. *et al.* Repeated quantum error detection in a surface code. *Nature Physics* **16**, 875–880 (2020).

[133] Bultink, C. *et al.* Protecting quantum entanglement from leakage and qubit errors via repetitive parity measurements. *Science Advances* **6** (2020).

[134] Luo, Y.-H. *et al.* Quantum teleportation of physical qubits into logical code spaces. *Proceedings of the National Academy of Sciences* **118** (2021).

[135] Marques, J. *et al.* Logical-qubit operations in an error-detecting surface code. *Nature Physics* **18**, 80–86 (2022).

[136] Bluvstein, D. *et al.* A quantum processor based on coherent transport of entangled atom arrays. *Nature* **604**, 451–456 (2022).
